# Offline generative network reconfiguration guides insight-like accelerated learning by assimilation into schema in rats

**DOI:** 10.1038/s41467-026-77318-1

**Published:** 2026-09-03

**Authors:** Baburam Bhattarai, George Dragoi

**Affiliations:** 1 Department of Psychiatry, Yale School of Medicine, New Haven, CT USA; 2 Department of Neuroscience and Wu Tsai Institute, Yale School of Medicine, New Haven, CT USA

**Keywords:** Consolidation, Neural circuits

## Abstract

Complex cross-modal de-novo associative learning generally requires numerous encoding exposures, but acquisition of an underlying mental schema of associative abstract rules enables insight-like accelerated new learning. Reports indicate that post-encoding sleep/rest offline epochs play an active role in insight learning, but the supporting neuronal ensemble mechanisms remained elusive. We developed a complex cross-modal learning task where six cue-place paired-associations (PAs) learned by male rats over weeks created a mental schema that enabled rapid within-day acquisition of 3-6 novel PAs. Simultaneous electrophysiological recording of hippocampus (HPC)-medial prefrontal (mPFC) ensembles across exploration-rest-sleep states indicated that accelerated learning of new PAs combined inferential activation of HPC map-based cue-place abstract associations and offline generative network reconfiguration in coordination with mPFC generalized outcome coding. HPC network reconfiguration during post-encoding sleep/rest predicted insight-like accelerated learning of 3-6 new PAs that consolidated and transferred rapidly via ripple-coordinated cell-assembly co-activation to mPFC. HPC ripple disruption during post-encoding sleep/rest prevented schema-based accelerated learning. Our findings reveal that map-based associative inference via offline predictive HPC-mPFC generative network reconfiguration supports insight-like accelerated learning by assimilation into schema and systems consolidation.

## Abstract (web summary)

Acquisition of a mental schema of associative abstract rules enables insight-like accelerated new related learning. The authors show that generative reconfiguration of hippocampus-prefrontal networks during sleep/rest in rats supports map-based accelerated learning and systems consolidation.

## Introduction

Rapid learning of new associations in a prior mental framework of known related acquired associations is thought to be achieved through the abstraction of the rules of association using prior knowledge of the task features, which are broadly termed mental schema^1,2^. An abstract associative learning framework based on mental schema, as opposed to short-term or episodic memory of specific prior experiences, is based on multiple independent yet related experiences, devoid of particularities related to any such independent experiences, and adaptable to rapidly and repeatedly incorporate new related information^3,4^. An existing associative framework conforming with these rules would (1) accelerate learning and consolidation of new, related associations well under the timeframe of the original de novo acquisition, (2) facilitate their stability over longer periods of time (e.g., days), and 3) update flexibly with incorporation of multiple and repeated new associations into the framework. Mechanistically, the medial prefrontal cortex (mPFC) is essential for both the activation^3,5^ of an existing associative framework and enabling accelerated new learning^6–9^, while the hippocampus (HPC) is essential for the de novo associative framework formation and accelerated new related learning^8–15^. In associative learning tasks, mPFC activity enables generalization across multiple conditions governed by the same rules, while HPC activity supports context-specific implementation of the rules^14,16^. Despite considerable progress, the key factors essential for the operation of a complex associative learning framework and the core HPC-mPFC neuronal ensemble mechanisms by which new, related associations are rapidly learned and consolidated have remained insufficiently understood.

The classic associative inference paradigms involve learning of two logical associations (e.g., A → B and B → C) separately, which then allows for successful association between the never experienced together elements (A → C) via serial associative inference. The HPC has been found to be critical in this inference process^10,11,17^. Previous studies on the role of HPC in schema-based accelerated learning^8,9^ and more broadly on abstract rapid learning have suggested that several possible mechanisms could be at play when a cue leading to an associated goal is rapidly replaced by a new cue-goal association. First, a serial associative inference by which the process of *direct cue feature binding and transfer* could rapidly associate the new cue with the original cue and, via original cue-goal location link, will associate the new cue to the goal location^18,19^. Second, a non-serial associative inference could alternatively occur by associating the reward outcome values to the experiences that led to the reward^20^ and building these representations over a relational cognitive map. This will create links between never-experienced associations over the map^21–23^, a process termed *map-based associative inference*. Third, a compositional inference that would serially regroup familiar items possibly via *generative offline* processes^24,25^ was suggested to underlie learning of logical sequences of items based on a previously acquired rule^26^. We designed a task that combined two paradigms: (1) the replacement of the original cue linked to a goal location with a new, different cue, which was previously not experienced together with the goal location and (2) simultaneous replacement of multiple paired associations (PAs) at multiple locations that could be arranged in a relational cognitive/spatial map. This task structure allowed us to ask which of the above mechanisms supported inferential rapid learning of multiple never experienced cues replacing the original ones via the simultaneously changed cross-modal associations with multiple unchanged goal locations.

Recently acquired memories re-activate offline in post-encoding slow-wave sleep (SWS) and waking rest periods in HPC within coordinated HPC-mPFC networks^27–31^. Furthermore, in humans, sleep/rest activity enhances memory and inspires problem-solving insight^32,33^ defined as a process of rapid novel association of previous conceptual nodes or elements, contributed by activation of the anterior temporal lobe and the hippocampus^34–36^, compared to a slower, deliberative way. However, besides a general replay of sequential memories, whether and how the neuronal ensemble temporal coding during offline post-encoding sleep/rest inspires insight and contributes to the rapid and stable changes across HPC-mPFC to promote accelerated associative learning and memory consolidation toward abstract problem solving has not been studied. Thus, to investigate our third hypothesis of compositional inference during sleep/rest, we studied whether the HPC network offline generative p/re-configuration competence^37,38^ uniquely re-purposes to support post-encoding HPC-mPFC network re-configuration as a form of continual offline learning. If true, this generative network re-configuration, but not simply the replay of previous sequential activity, would be predictive of (1) future post-sleep/rest insight-like increases in animal behavioral performance, (2) future neural correlates of an updated mental schema, and (3) rapid memory consolidation within the HPC-mPFC network. Altogether, we aimed at elucidating the mechanisms of how HPC and mPFC networks supporting an associative learning framework (e.g., cognitive schema) adapt and interact rapidly across brain states to improve animal performance while keeping the associative linking rules stable across repeated accelerated abstract learning.

We developed a complex associative schema framework task by subjecting our rats to learning 6 cue-place paired associations (PAs) in an 8-arm radial maze, which they acquired over 6–8 weeks of training. This enabled us to investigate whether and how this associative memory framework: (1) dynamically and repeatedly supported the accelerated within-day assimilation of 3–6 new PAs, (2) consolidated the newly assimilated associations rapidly and remained stable across several days, and (3) evolved flexibly during repeated assimilation of different sets of new PAs. We explored if alternative specific and non-specific frameworks could have led to accelerated learning by controlled-altering the context in which the original associative framework was formed. Therefore, we investigated whether activation of memories of the original PAs (i.e., direct cue feature binding and transfer) or, instead, the activation of abstract associative rules in a relational framework (i.e., map-based associative inference) would allow for the rapid mental replacement of the original PAs within the pre-existing framework.

Learning causes continual changes in the underlying neuronal networks, which reconfigure to support new learning^21,39–41^. However, how these continual reconfigurations supporting novel as well as unchanged (i.e., congruent) aspects of memories are organized offline in HPC-mPFC networks during post-encoding sleep/rest and the consolidation phases of the assimilation process have remained poorly understood. Our use of a 6 PA-paradigm allowed us to repeatedly change half (i.e., 3) to all (i.e., 6) PAs at a time to study the offline reconfiguration of neural representations of unchanged (congruent) and changed (novel) PAs across HPC and mPFC ensembles. Because HPC sharp wave ripples (SWRs) are critically linked to spatial learning^29,42,43^, we tested if ripple-related neuronal ensemble activity during post-encoding sleep/rest was critical for insight-like^32,34,36^ task-solving via rapid abstract learning of numerous associations simultaneously. For this, we artificially disrupted HPC activity during those ripple-associated epochs using closed-loop electrical stimulation of the ventral hippocampal commissure and tested the impact this targeted disruption had on our 6-PAs rapid learning and consolidation task by assessing animal behavioral performance.

## Results

### Schema-based rapid assimilation learning of paired associates

**Fig. 1:**
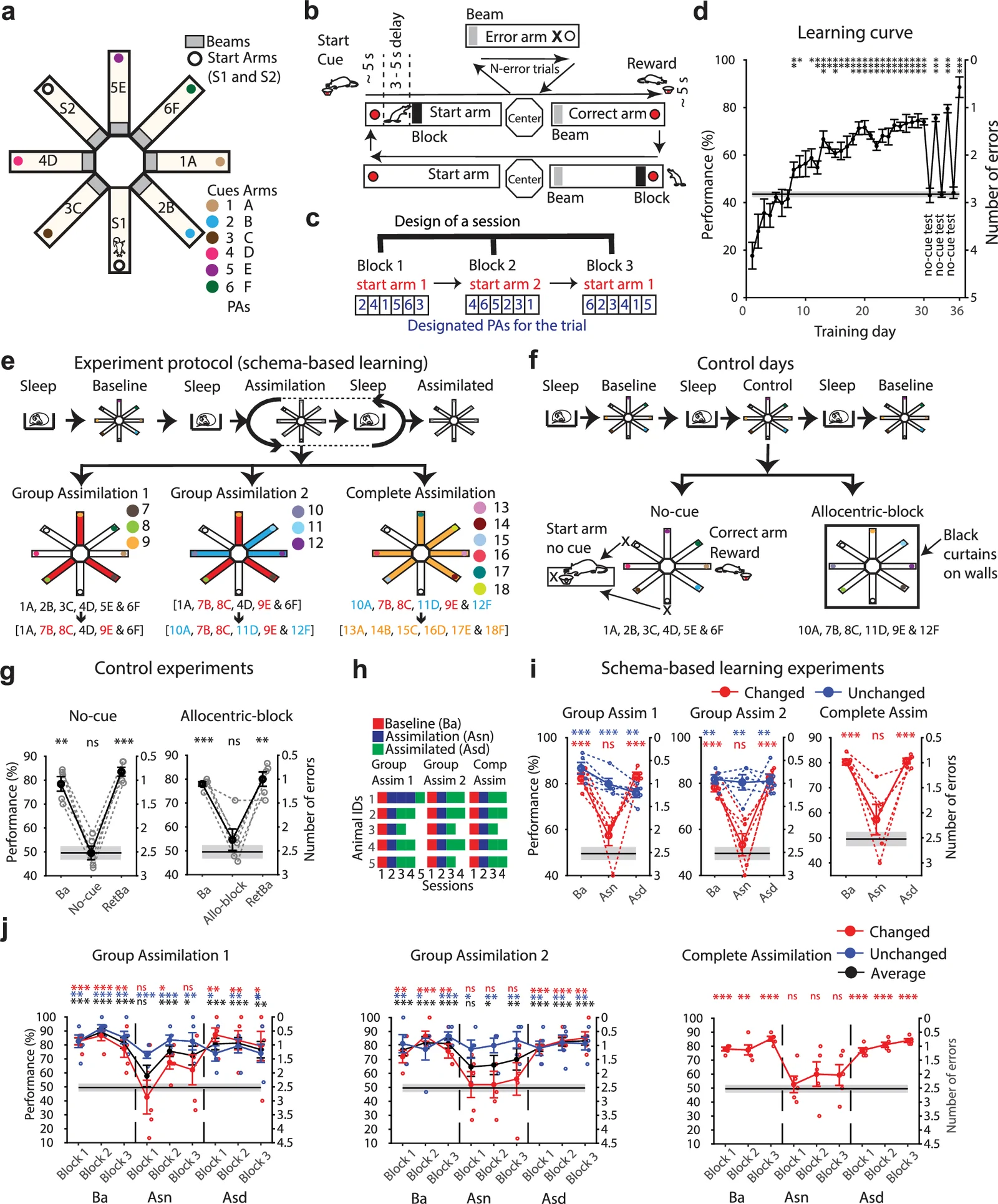
Training and performance of rats in the schema-based rapid learning task. **a** Eight-arm radial maze used for learning the 6 cue-place paired associates (PAs) task. Schematics of a trial (**b**) and a session (**c**). **d** Learning curve depicting increased performance (left ordinate) and decreased errors (right ordinate) across training days and no-cue tests compared against chance levels (Methods). **e** Protocol for schema-based rapid learning experiments. Changed PAs are indicated on corresponding maze-arms colored in red, blue, and orange for group assimilation 1, 2, and complete assimilation experiments, respectively. Protocol (**f**) and performance (**g**) on no-cue and allocentric-block control experiments (no PAs were changed). **h** Categorization of sessions into learning stages across animals and experiment days. **i** Average performance for changed and unchanged PAs across days and sessions during schema-based assimilation learning experiments compared against chance levels. **j** Performance across blocks for changed and unchanged PAs and their combined average during the 3 assimilation learning days (*N* = 5 rats). Respective blocks from categorized sessions (**h**) were included in the analyses of (**i**) and (**j**). Two-tailed paired t-tests in (**d**, **g**–**j**); *N* = 7 rats in (**d**) and 5 rats in (**g**–**j**). Detailed statistics are in Supplementary Data 1. PAs Paired associations, Ba Baseline, RetBa return-to-Baseline, Asn Assimilation, Asd Assimilated, Allo Allocentric. Data are shown as mean±s.e.m. Source data for (**d**, **g**, **i** and **j**) are provided as a Source Data file. ns=not significant, *P* > 0.05, ***P < 0.001, **P < 0.01, *P < 0.05.

We subjected our rats to a 30-day-long (spread across 6-8 weeks) de novo learning of an associative framework enabling parallel retrieval of 6 distinctly flavored food rewards from 6 associated remote goals at 6 track-ends of an 8-arm radial maze. The rats were cued at the remaining 2 track-end locations (called start arms) with the goal-specific cue (i.e., flavor reinforced by odor) of each trial (Fig. 1a, b). The proximal part of all the goal arms of the maze and of the other start arm were elevated using beams (10 cm high, 6 cm wide, and 20 cm long, with horizontal surface parallel to the remaining segment of the track), such that rats spent an extra energy cost of climbing up and down the beams to visit each arm end. Each daily session had 3 blocks of 6 trials/block (one trial for each of the 6 cues, presented in randomized order) with the 2 start arms alternating between consecutive blocks to prevent habitual rat behavior (Fig. 1c). The rats first improved their performance from 17.62 ± 5.6% (4.1 ± 0.3 errors/trial) on the 1st day to 56.34 ± 5.4% (2.2 ± 0.3 errors/trial) on the 10th day primarily by not visiting (1) the start arm not cued during the block, (2) the arms already visited during the trial, and (3) the arm rewarded in the previous trial of the block (Supplementary Fig. 1a-d). Learning these non-associative rules alone improved rats’ performance from 17.62 ± 5.6% (4.1 ± 0.3 errors/trial) on the 1st day to 56.34 ± 5.4% (2.2 ± 0.3 errors/trial) on the 10th day. At the end of training, rats performed 3 no-cue test control sessions in which no cues were provided at the start arms while the flavored food rewards continued to be delivered at their corresponding goal locations and all experimental procedures were kept constant (Fig. 1d). This transient decoupling of cue-place association rule from all the other non-associative rules enabled us to obtain the experimental chance-level performance for this associative task. Rats’ performance consistently exceeded the chance levels starting on the 11th day of training (Fig. 1d). Completion of training was marked by plateauing of the learning curve above 70% performance and below 1.5 average errors/trial for 3 consecutive days, which our rats achieved by use of associative memory in the task (Fig. 1d and Supplementary Fig. 1e–h).

The rats that successfully acquired the original 6 PAs-associative task were implanted with electrode arrays, and neural activity was recorded simultaneously from the CA1 area of the HPC^31,44^ and the prelimbic region of the mPFC during animal behavior. The implanted animals performed multiple behavioral sessions in the maze, each preceded by sleep/rest sessions in an off-maze sleep box across 3 distinct assimilation learning conditions and 2 control days (Fig. 1e, f), as detailed below. Following post-implantation retraining to criterion over 7 days, animals went through an 18-trial no-cue control session preceded and followed by original baseline 6-PAs sessions (baseline and return-to-baseline), all interleaved by sleep/rest sessions (i.e., no-cue control day, Fig. 1f, g and Supplementary Fig. 2). Subsequently, we tested rapid learning of 3 new PAs (#7-9 replacing original PAs #2, 3, 5) along with 3 unchanged PAs #1, 4 and 6 as a process of *group* assimilation into the pre-existing associative framework (Fig. 1e). We classified sessions on all assimilation experiments days into *baseline* (the first session), *assimilation* (sessions with <70% performance for the changed PAs), and *assimilated* (sessions with >70% performance for the changed PAs, Fig. 1h). On this group assimilation experiment, rats performed variably around chance level for the changed PAs (at chance in the first and last blocks, marginally above chance in the second), but significantly better than chance for the unchanged PAs in all blocks during assimilation sessions (Fig. 1i and Supplementary Fig. 2). Intriguingly, generally after only one assimilation session (3 exposures to each changed PAs across 3 blocks in 4 rats out of 5) followed by a sleep/rest session, their performance improved significantly in the following assimilated sessions for both changed and unchanged PAs (Fig. 1h–j and Supplementary Fig. 2). This showed that rats learned the new sets of 6 PAs (3 changed, 3 original PAs) very rapidly after only one exposure-encoding session and a post-encoding offline sleep/rest session in a sleep box. This rapid learning was likely accelerated by their use of a pre-existing associative framework of the task, given the de novo learning of the original 6 PAs spread over >5 weeks.

To uncover the possible mechanisms underlying this rapid associative encoding and learning, we first investigated whether the set of 3 original unchanged PAs were necessary, likely by acting as critical anchors in a relational framework of declarative facts enabling learning of the 3 new PAs. Thus, we explored whether the newly learned 3 PAs acting as part of an updated schema were sufficient to further support rapid assimilation of a new set of 3 PAs (#10-12) by replacing the 3 remaining original PAs (#1, 4 and 6) in the same rats, now in absence of all original 6 PAs (Fig. 1e). Interestingly, on this second group-assimilation experiment, rats’ levels of performance across sessions for changed (i.e., chance-level in all blocks) and unchanged (above chance-level in all blocks) PAs were similar to the first group assimilation, despite the absence of all original PAs (Fig. 1i, j and Supplementary Fig. 2). All rats achieved significantly better performance than chance for the changed and unchanged PAs after only one exposure-encoding session (3 trials for each new PA) followed by a post-encoding offline sleep/rest session in the sleep box (Fig. 1h, j).

To further validate whether the original PAs were dispensable and an updated configuration was sufficient for rapid assimilation learning, we conducted a third, *complete* assimilation learning experiment, when we changed all previously learned PAs with a complete set of 6 new PAs (#13-18; Fig. 1e). Surprisingly, the rats were able to learn the newly changed PAs as fast as they did on the previous days of group assimilation experiments, after one exposure-encoding session and a post-encoding offline sleep/rest session (Fig. 1h–j and Supplementary Fig. 2). This rapid complete assimilation (1) confirmed the associative framework didn’t necessarily rely on the specific relationship among the original or previously learned PAs but rather on an associative abstract rule, (2) cross-validated our earlier findings on rapid group-assimilation into the associative framework, and (3) suggested a key role for the unchanged allocentric spatial map in rapidly anchoring the novel PAs to the known goal locations. Therefore, in a second control experiment day, we blocked access to all allocentric room-wall visual cues with opaque black curtains during performance of a familiar set of 6 PAs, preceded and followed by baseline maze sessions with unchanged 6-PAs and full access to original allocentric cues (Allocentric block day, Fig. 1f). The allocentric cue blocking led to a significant drop in rats’ performance to chance levels, which recovered when the allocentric block was removed (Fig. 1g and Supplementary Fig. 2). This suggests the rats relied on a stable map-based allocentric localization of goals and PAs to solve the schema task, as opposed to a mental binding of the local maze cues with the egocentric goal locations.

Altogether, our rats performed at chance level for the newly changed PAs in all but one blocks during assimilation sessions across all 3 assimilation learning days, while they remembered well the unchanged PAs in all blocks (Fig. 1i, j and Supplementary Fig. 2). However, after encoding the new PAs and a post-encoding sleep/rest session, in the assimilated sessions of all 3 learning days, rats performed significantly better than chance for all the changed and unchanged PAs (Fig. 1i, j and Supplementary Fig. 2). Animals continued to remember the updated configuration of the PAs, 2 and 2-4 days after the first and the second group assimilation learning, respectively, indicating a long-term stability of an updated associative framework of the recently learned PAs (Fig. 1g, i, j and Supplementary Fig. 2). The new and the original-replaced cue flavors had similar distributions of relatedness (i.e., overlapping flavor ingredients^45^), and the cue relatedness level did not significantly affect the behavioral associative learning performance (Supplementary Fig. 3). Both first-presented (i.e., primary) and last-presented (i.e., recent) new PAs were learned similarly across blocks and sessions (Supplementary Fig. 4a). Schema dramatically (*P* = 1.55 × 10^−^^4^) accelerated the learning of new PAs, which required only 1.08 ± 0.08 sessions compared to 16.25 ± 2.20 sessions during the de novo training (Supplementary Fig. 4b). Intriguingly, performance for all new PAs worsened together in the assimilation session, but, given an interim offline sleep/rest session, displayed a concerted dramatic, insight-like, accelerated significant improvement in the first block of assimilated session, simultaneously for all 3-6 new PAs during learning days (Supplementary Fig. 4c–g). Therefore, the associative framework was (1) flexible and dynamic, enabling repeated assimilation of group and complete sets of new PAs across days enabling successful problem solving, (2) stable in absence of new learning over several days, (3) dependent on the allocentric-context in which it was originally established, (4) non-episodic and without serial position effect, and (5) not active when rats employed non-associative strategies. These features fulfill the stricter criteria commonly attributed to mental schemas^3,4^.

### Neural mechanisms of schema-based rapid assimilation learning

**Fig. 2:**
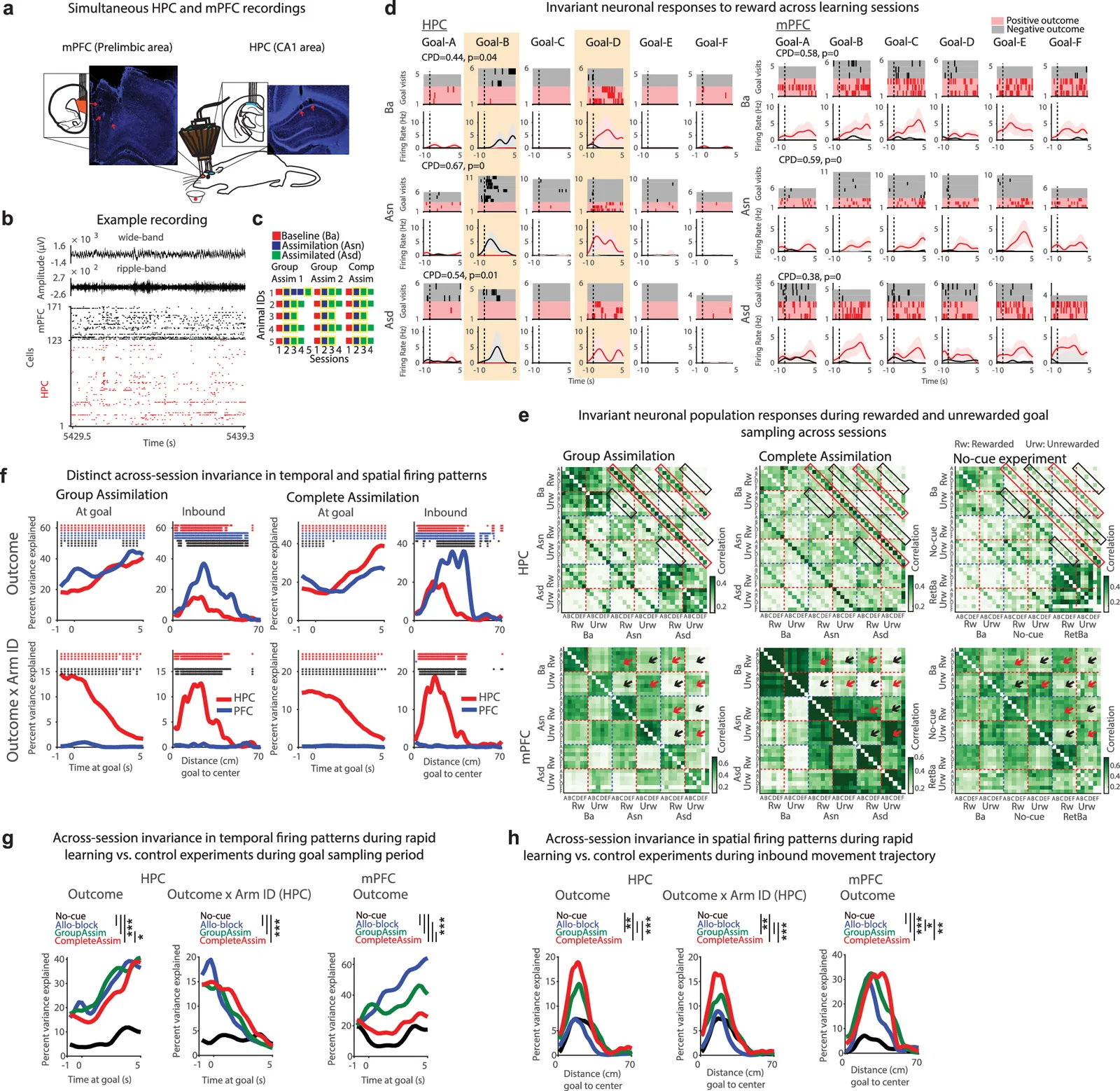
Outcome and goal-specific outcome invariant responses in mPFC and HPC during rapid learning of new PAs. Illustration of simultaneous electrophysiological recordings during schema-based learning: cartoons and histological sections showing electrode tracks and tips (red arrows) in mPFC (**a**, left) and HPC (**a**, right); example recording of wide-band and ripple-band HPC LFP (**b**, top and middle) and HPC-mPFC spikes (**b**, bottom). **c** First assimilation and assimilated (yellow borders) sessions were included in the neuronal analyses. **d** Example HPC cell (left) responding invariantly to absence of reward at goal-B and presence of reward at goal-D, and mPFC cell (right) responding invariantly to reward at all goals across learning sessions (rows). For each example, visit-by-visit peri-event rasters are shown at the top and the firing rates at bottom, at the time of port entry at 6 different reward ports (goals A-F shown in columns), separately for the presence and absence of reward. **e** Representational similarity matrices (RSMs) for neuronal ensemble activation at the 6 goals with two outcome types (demarcated by red dashed lines), each across three session types (demarcated by blue dashed lines). Note higher population vector correlations for the same (red rectangles) compared to the opposite outcome type (black rectangles) at specific goals in HPC (top), and the same (red arrows) compared to the opposite outcome type (black arrows) irrespective of goal identities in mPFC (bottom). **f** Percentage of variance explained by invariance of outcome types (same|different), and outcome types at specific arms (specific|unspecific; linear regression, see Methods) on the across-session portion of RSMs. Results were calculated in temporal (25 ms, left) and spatial (1 cm, right) bins and compared between HPC and mPFC population vectors. Significance: red for HPC, blue for mPFC comparing data vs. shuffles; black for difference in regions vs. difference in shuffles. Results obtained in (**f**) were compared among No-cue, Allocentric-block, Group, and Complete Assimilation experiments for the temporal representations at goal sampling (**g**) and spatial representations during inbound movement (**h**). Two-tailed Student’s *t*-tests, *N* = 25 at goal and *N* = 70 in the outbound trajectory. Neuronal ensembles composed of *N* = 412 (HPC) and 122 (mPFC) in Complete Assimilation, *N* = 758 (HPC) and 264 (mPFC) in Group Assimilation, *N* = 309 (HPC) and 83 (mPFC) in No-cue, *N* = 317 (HPC) and 116 (mPFC) in Allocentric block experiments, were analyzed in (**e**–**h**). Detailed statistics are in Supplementary Data 2. Ba Baseline, RetBa return-to-Baseline, Asn Assimilation, Asd Assimilated, Allo Allocentric. Data are shown as mean±s.e.m in (**d**). Source data for (**e**–**g**) are provided as a Source Data file. ***P < 0.001, **P < 0.01, *P < 0.05.

Our first hypothesis was that an inferential mental substitution of the original cue with the new cue, both associated with the same goal location, could occur via a neural mechanism of direct cue feature binding and transfer (i.e., cue-anchored). One of the ways this could work is via the activation of the representation of the original cue during the encoding of the new cue-goal association at the goal location, thus inferentially substituting the never-experienced-together old for the corresponding new cue^18,19^. We tested this hypothesis by studying the neuronal ensemble correlates of PAs, namely place- and cue-related activities in the HPC and mPFC across sessions. We recorded simultaneously using 128 recording sites evenly distributed across each side of the HPC and the right mPFC (Fig. 2a, b; Supplementary Table 1; see Methods) from a total of 2011 HPC and 762 mPFC neurons across the 3 distinct assimilation learning conditions and 2 control days (mean 101 ± 6 HPC and 37 ± 3 mPFC neurons/rat/day). First, we found no systematic difference in HPC place field remapping (Supplementary Fig. 5a–c) or proportion of cells maintaining place fields on the same arms (Supplementary Fig. 5d) across same-day learning sessions, when grouped by similarly elapsed time. Next, we asked whether rapid learning was aided by repurposing of the cue-responsive cells from the original to the corresponding new cue associated with the same goal, invariant to the introduced changes in PAs. We identified specific cue-responsive cells (Supplementary Fig. 5e) as the neurons that selectively and transiently increased or suppressed their firing rate in conjunction with presentation of a specific flavor cue regardless of its location on the start arm or the end of goal arms. Both HPC and mPFC ensembles of cells responsive to the unchanged cues maintained a significantly greater similarity in their cue-activation between baseline and assimilation sessions (correlation 0.29 ± 0.02 for HPC, 0.32 ± 0.03 for mPFC ensembles) compared to cells responsive to the changed cues (0.10 ± 0.03 for HPC, 0.21 ± 0.03 for mPFC ensembles; Supplementary Fig. 5f, g). Moreover, we found a reduced overlap in cue-responsive HPC cells (Supplementary Fig. 5h) that maintained corresponding cue responsiveness from baseline to other sessions for the changed (15.60 ± 3.4%) compared to unchanged cues (35.94 ± 4.6%) (*P* = 7.3 × 10^−^^3^; Supplementary Fig. 5i). Similarly, in complete assimilation day, the proportion of cue-responsive cells maintaining cue-responsiveness from baseline to other sessions (i.e., new, different cues) was smaller compared to the proportion that did so from assimilation and assimilated sessions to the other assimilation sessions in HPC (i.e., same cues), and from assimilated session to the other sessions in mPFC (18.03 ± 5.9, 50.05 ± 4.9 and 47.3 ± 6.8% of HPC cells and 31.09 ± 5.9, 55 ± 9.6 and 60.48 ± 5.5% of mPFC cells for baseline, assimilation, and assimilated sessions, respectively, Supplementary Fig. 5i). The cue responding neurons were originally quantified in comparison to the trial-ID shuffles. In the no-cue test session, the task demand was altered (non-associative), and the reward cues were not changed, while in the allocentric-block session cues were not changed, and the task demand was still the same. Interestingly, in the no-cue session (Supplementary Fig. 5i) we saw similar neuronal responses to no-cue (i.e., chance level) compared to the changed cues, and in the allocentric-block session we saw similar results to the unchanged cues compared to the learning sessions. This suggests that (1) the responses to the original vs. the changed cues are not more similar than chance (i.e., responses to cue vs. no cue presentation) in our schema-based learning experimental design, and (2) a neuronal cue response maintained at the level of an unchanged cue, as occurring in the allocentric block session, is not sufficient to warrant successful behavioral performance or new learning. We could not directly compare the overlap in the network representation of changed cues with the original baseline cues during schema-based learning to the ones in non-schema learning conditions due to the different focus of our experiment design. Therefore, when cue identities changed, network cue-responsiveness changed as well^46^ compared to the cue-responsiveness to the unchanged cues, suggesting that the inferential learning in this schema-based task might not have happened solely through the representational generalization of cue features, i.e., our first hypothesis of direct cue feature binding and transfer.

Our second hypothesis was that assimilation of the new PAs happened by activating and associating invariant neuronal representations linked to the task framework, namely a direct association of goal locations with the corresponding correct cues in this task, irrespective of the cue identities (i.e., original or new). We hypothesized that these invariant neuronal representations co-activated and updated the new cue-outcome contingencies (positive, rewarded as well as negative, not rewarded) at goal locations, which would link the rewarded goal locations with the correct/corresponding new cues. Once the newly encoded corresponding cue-goal location association begins consolidation via offline orderless neuronal co-activation it would gain symmetry, such that activation of the new cue at the start arms would become predictive of the corresponding goal location. We termed this neural mechanism of rapid learning map-based associative inference. We next tested the map-based associative inference hypothesis by first studying the HPC-mPFC neural correlates during arm visits (Supplementary Fig. 6). To test this hypothesis, we analyzed the data from the baseline, the first assimilation, and the first assimilated sessions (Fig. 2c). Before analyzing the across-session similarities in neural representations, we first studied the within-session analyses of variances in single-unit firing rates. We conducted linear regression analysis using visit-by-visit firing rates on all goal arms (goal sampling, inbound, and outbound movement trajectories) as the dependent variable, the reward, arm IDs, and their interaction as factors, and velocity, linear and lateral position of the rat, and theta power as covariates. HPC single units showed robust representation of arm IDs (Supplementary Fig. 6a), reward as well as reward associated with specific arm IDs when rats were at goals and when moving inbound from goals toward maze center (Supplementary Fig. 6d). In contrast, mPFC single units showed reward representation irrespective of arm IDs when rats were at goals in all learning and control experiment sessions (Supplementary Fig. 6d). Next, we studied the relationship of the basic properties of single neuronal units with these task-related variances. We found that mPFC single unit burst indices were positively correlated, and HPC single unit theta phase modulations were negatively correlated, with the task-related variances in single unit firing rates during learning (Supplementary Fig. 7). Furthermore, we observed that HPC single units that responded to reward at specific goal locations in the baseline session during learning consistently responded to the reward at the same goal location in assimilation and assimilated sessions irrespective of whether the PAs were changed or unchanged at the goal (Fig. 2d and Supplementary Fig. 6b). Similarly, mPFC single units that responded to the reward consistently did so in all sessions during learning (Fig. 2d and Supplementary Fig. 6c).

To quantify the invariance in neuronal representations across sessions, we conducted population vector (PV) correlations of neuronal activity stacked across all rats either in the spatial (outbound and inbound trajectories) or temporal domains (at goals). PV correlations were computed across the 2 possible outcomes and 3 session types for each of the 6 goals and displayed as representational similarity matrices (RSMs; Fig. 2e and Supplementary Fig. 8). The RSMs indicated that the invariant neuronal representations occurred for the same outcomes (positive or negative reward) across the learning sessions. These invariant activations of neuronal PVs were specific to the same goals in HPC (depicted by the red and black diagonal boxes in Fig. 2e and Supplementary Fig. 8) and were generalized irrespective of the goals in mPFC (red and black arrows in Fig. 2e and Supplementary Fig. 8). These outcome-specific invariant responses reduced and became more variable across sessions in the no-cue control experiment day (Fig. 2e and Supplementary Fig. 8). Despite the reduction in across-session outcome-invariant activations, the animal behavior and HPC theta power on both these outcome types were not different between the no-cue sessions and the assimilation sessions of the learning days (Supplementary Fig. 9a–c). This indicates that the increase in outcome-invariant responses across sessions during schema-based learning compared to the control day experiment was not due to differences in reward, behavioral, or theta oscillation state of the animals. Therefore, these results suggest that the cross-session goal-specific outcome-invariant responses likely linked the new cues to the goal-specific outcome, similar to the corresponding original cues, contributing to the rapid assimilation process.

We next studied the temporal dynamics of the invariant neural representations across sessions to directly compare them between the rapid learning and the control experiment days. We quantified the contribution of different factors to the invariance across sessions. For this, we first excluded within-session correlations and conducted linear regression on the cross-session PV correlations as the dependent variable. We used the similarity in the outcome (same/different), goal IDs (specific/non-specific), their interaction, and session IDs as different factors, and the similarities in the rats’ velocity, position and theta power as covariates (Supplementary Fig. 9d). Although the behavioral states during assimilation learning sessions were generally different between rewarded and unrewarded arm visits, they were similarly different during the control sessions and were not systematically invariant across learning sessions to account for the invariance of neural representations (Supplementary Fig. 9). Regression analyses conducted on bin-by-bin temporal (25 ms bins for goal periods) PV correlations revealed that a significant proportion of the invariances in representation across sessions was explained by similarity in outcome at specific goals in the HPC, and in outcomes irrespective of goals in mPFC (Fig. 2f). Similar analyses conducted on bin-by-bin spatial (1 cm bins for spatial firing patterns) HPC PV correlations, revealed that the outcome-associated invariant representations at specific goals activated across sessions in the spatial representations during inbound movement after goal sampling (Fig. 2f). These invariances were observed and explained by the outcome similarly during the complete assimilation days (Fig. 2f–h), but were impaired during the control days (Fig. 2g, h). During the no-cue day, the proportion of invariance in neural representation explained by similarity in outcome specific to the corresponding goal was reduced at goals and in the inbound movement trajectories (Fig. 2g, h). This reduction occurred despite the no-cue session being similarly rewarded and rats behaving similarly compared to the assimilation sessions (Supplementary Fig. 9a–c). This indicates the invariant neural representation during rapid learning depended on the outcome being associated with the corresponding cues rather than the reward value. The goal-specific invariance explained by outcome was also reduced in spatial representations during inbound movement on allocentric-block control days, suggesting its dependence on the stability of allocentric-context/map (Fig. 2h). The reproducible effects observed across the group and complete assimilation days suggest they shared similar HPC network mechanisms, independent of the presence/absence of familiar PAs (Fig. 2e–h) and the trial-specific episodic information. The across-session invariant activation of distinct outcome codes is consistent with the activation of pre-existing rules of the schema, i.e., that one cue leads to reward at one goal and other cues lead to no reward at the same goal (applied to multiple goal sites), enabling rapid inferential assimilation learning. The activation of a more general outcome-dependent invariant neural representation in mPFC, however, depended on the outcomes being associated with the cues as well as on the stability of the PAs. This is indicated by the reduced activation of general outcome contingencies at goals during the no-cue and complete assimilation days (Fig. 2g). We found a similarly robust activation of goal specific-associated outcome contingencies in the HPC and general outcome rule in mPFC when we restricted our analysis to only the first block of assimilation sessions (only correlations involving the assimilation sessions were included; Supplementary Fig. 10). Therefore, our findings demonstrating immediate activation (i.e., during first exposure) of map-based associated outcome contingencies in the HPC indicate that rapid map-based associative inference is the most likely HPC mechanism of rapid assimilation of novel PAs in our task.

### Predictive associative memory activation in the HPC

**Fig. 3:**
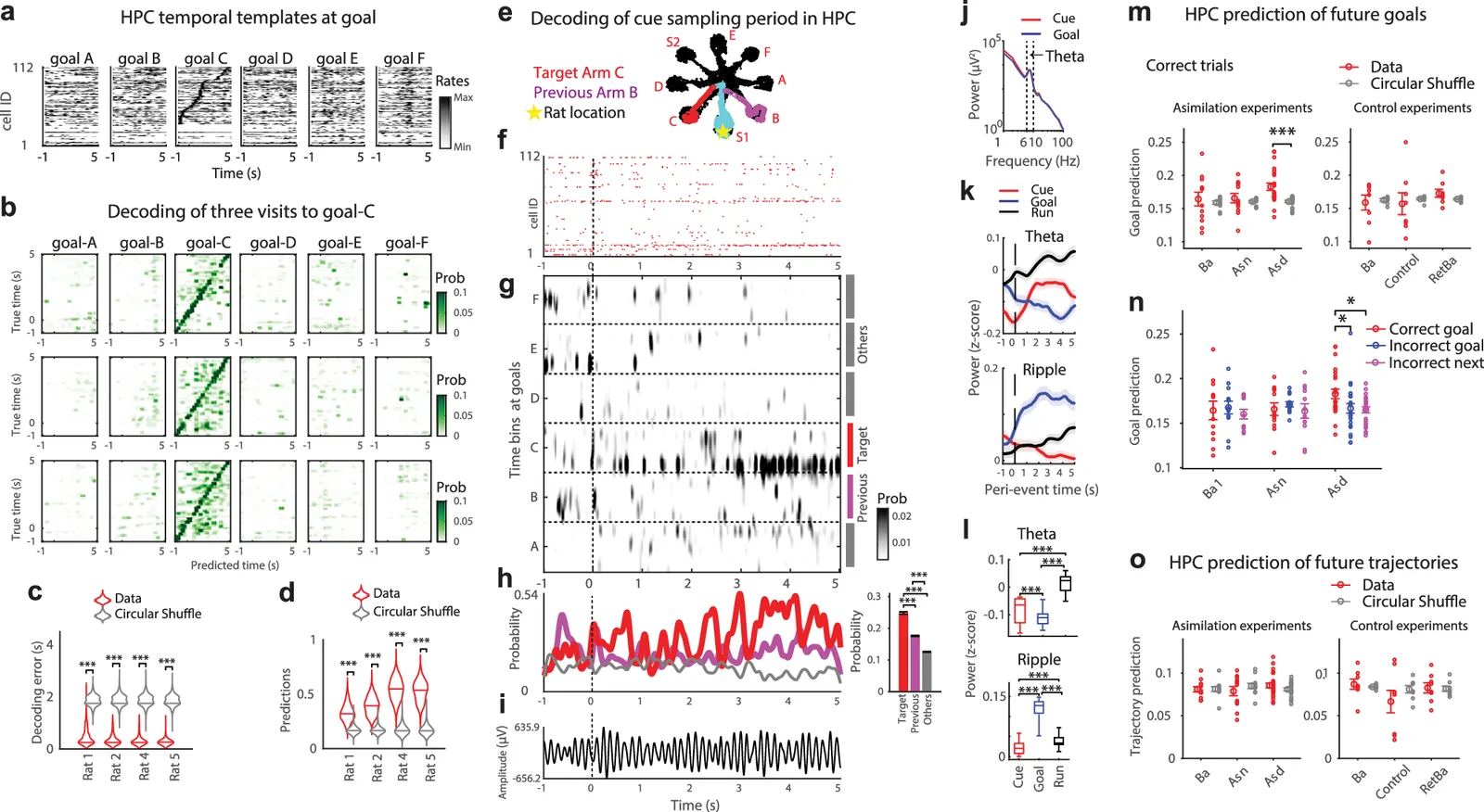
HPC ensembles at start-arm cue presentation became predictive of goal identities during rapid associative learning. **a** Example HPC temporal sequence template for goal-C compared to all other goals based on the order of time of peak average firing of neurons at goal-C. **b** Decoding of instantaneous HPC activity during 3 correct/rewarded goal visits to goal-C (rows) using concatenated templates of all A-F goals as prior. Heatmaps show probabilities of predicted time for all 6 goals (columns) against true time spent at goal-C. Average (median) instantaneous decoding errors (**c**) and predictions of goals (**d**) compared against shuffles (two-tailed rank-sum tests). **e** Example correct trial during the assimilated session (current rat location at start arm S1). **f** Spiking raster plot for HPC pyramidal neurons during peri-cue sampling epoch for the example trial in (**e**). **g** Decoding of HPC ensemble activity during peri-cue sampling epoch (x-axis, 50 ms bins) using the concatenated 6 templates at goal (250 ms time bins). Resulting posterior probabilities for 6 goals were stacked on the y-axis for the example trial in (**e**). **h** Sum (left) and mean sum±s.e.m. (right) of decoded probabilities for prediction of previous trial goal (magenta), future target goal (red), and other goals (gray) (two-tailed rank-sum tests, *N* = 601 for each) for the example trial in (**e**). **i** Theta oscillation amplitude during peri-cue sampling epoch for the example trial in (**e**). **j** HPC power spectrum during cue- and goal-sampling (6 s each). HPC theta (top) and ripple (bottom) peri-event power (**k**) during cue and goal sampling as well as post-cue sampling run periods (6 s each); these powers were compared in (**l**) (two-tailed rank-sum tests, *N* = 1200 for each). **m** Average posterior probabilities of future goal prediction for correct trials for each session type of assimilation learning and control days, and their corresponding shuffles (two-tailed signed-rank test; *N* = 24 for assimilated sessions). **n** Future goal prediction in correct trials compared against correct goal and incorrect next goal predictions in incorrect trials during assimilation days (two-tailed signed-rank test; *N* = 24 for assimilated sessions). **o** Average posterior probabilities of upcoming arm trajectory prediction for correct trials for each session type of assimilation learning and control days, and their corresponding shuffles (two-tailed Signed-rank test; *N* = 24 for assimilated sessions). Ba Baseline, RetBa, return-to-Baseline Asn Assimilation, Asd Assimilated. Data in (**h**, **k,** **m**–**o**) are shown as mean±s.e.m. Data in (**l**) are shown as boxes of 25th–75th percentiles with median in the center and whiskers covering twice the interquartile range. Detailed statistics are in Supplementary Data 3. Source data for (**c**, **h**–**o**) are provided as a Source Data file. ***P < 0.001, *P < 0.05.

We hypothesized that in the map-based associative inference process, the activation of correct goal representations during the cue presentation at the start arms might inferentially guide the new cue-goal associated behavior. In this scenario, we would expect to observe activation of the associated outcome-specific goal representations when the rats sampled the correct cues at the start arms. To test this hypothesis, we investigated whether specific cues evoked a predictive activation of representations of outcomes at the associated goals when sampled at trial initiation in the start arms during assimilation learning. We first validated a Bayesian decoding algorithm^31,44,47,48^ by successfully decoding the instantaneous hippocampal ensemble activity at goal periods during positive outcomes using the temporal ensemble activations at the six positive goal sampling periods as goal-template priors (Fig. 3a–d). Next, we decoded HPC neuronal ensemble activity during cue-sampling epochs on start arms across sessions using the same goal-template priors and found significantly higher predictions for the future correct goals in the correct trials (0-error trials) specifically during the assimilated sessions of assimilation learning days (Fig. 3e–m). Those cue sampling epochs at start arms had reduced ripple-band and increased theta-band power compared to the goal sampling epochs, despite rats being immobile during both epochs (Fig. 3j–l). During incorrect trials (>0 errors), this increased predictability was absent for either the correct goals (i.e., when not immediately visited) or the immediately visited goals (i.e., erroneous) when decoded at cue-sampling periods in assimilated sessions (Fig. 3n and Supplementary Fig. 11). Since the reward delivered at the correct goal contained the flavor of the corresponding cue, we asked whether the cue-induced predictive activation of goal representation in the start arm was confounded by the sensory cue re/presentation. If that were the case, the cue presentation epochs in the baseline and allocentric-block sessions should have been equally predictive of the correct future goals, but we did not observe that (Fig. 3m). To further investigate this predictive activation, we ran an additional internal control. We added epochs of ‘non-current’ corresponding-cue re/presentations (i.e., temporal firing rate templates) computed when rats sampled the same cue at start arms in blocks other than the current block in the same session (i.e., not in the current block and not at the goal location) to the epochs of goal-sampling templates/representations. This resulted in a combined new prior that we used to re-decode the local cue-sampling epochs during assimilated sessions. If sensory cue responses were the primary driver of predictive goal representations, we would expect to observe an increased goal prediction with the addition of sensory cue responses to the goal templates as new prior. In contrast, the addition of cue-responses to the goal templates did not improve the goal predictions during the cue-sampling epochs (Supplementary Fig. 12). Thus, we found no evidence that sensory responses to the cues were the primary driver of the goal predictions during the assimilated sessions. The lack of prediction in all the other sessions (and in the incorrect trials) except in the assimilated session implies an inferential activation of the latent causes associated with the positive action-toward-goal, resulting in the positive outcome at goals, which likely guided the correct behavior during rapid inferential learning. Additionally, there was a general suppression of HPC prediction for the previously rewarded goals when rats made correct predictions for the upcoming goals in assimilated session trials (Supplementary Fig. 11d). In contrast, we observed an interference (i.e., positive correlation) between the predictions for the upcoming goal and for the previously rewarded goal during the allocentric-block control session (Supplementary Fig. 11d), which could partly explain rats’ poor performance in that session. This indicates that an accurate neuronal predictive representation of the future goal with minimal interference from the past goal supported successful animal performance during inferential learning. Intriguingly, HPC neuronal ensembles did not predict the upcoming future trajectories toward goals during cue presentations in both correct (Fig. 3o) and incorrect assimilated session trials (Supplementary Fig. 11). The predictive activation of future correct goals was higher than shuffles in trial-wise comparison overall and within subjects (3 out of 4 animals, Supplementary Fig. 11e–g). This suggests that, in our task, HPC predictive activity at trial initiation was specific to goal representation. Predictions of future correct or incorrect next goals were generally not observed when we decoded activity of mPFC ensembles during the cue-sampling epochs (Supplementary Fig. 11h–k).

### Inference and rapid network reconfiguration during sleep

**Fig. 4:**
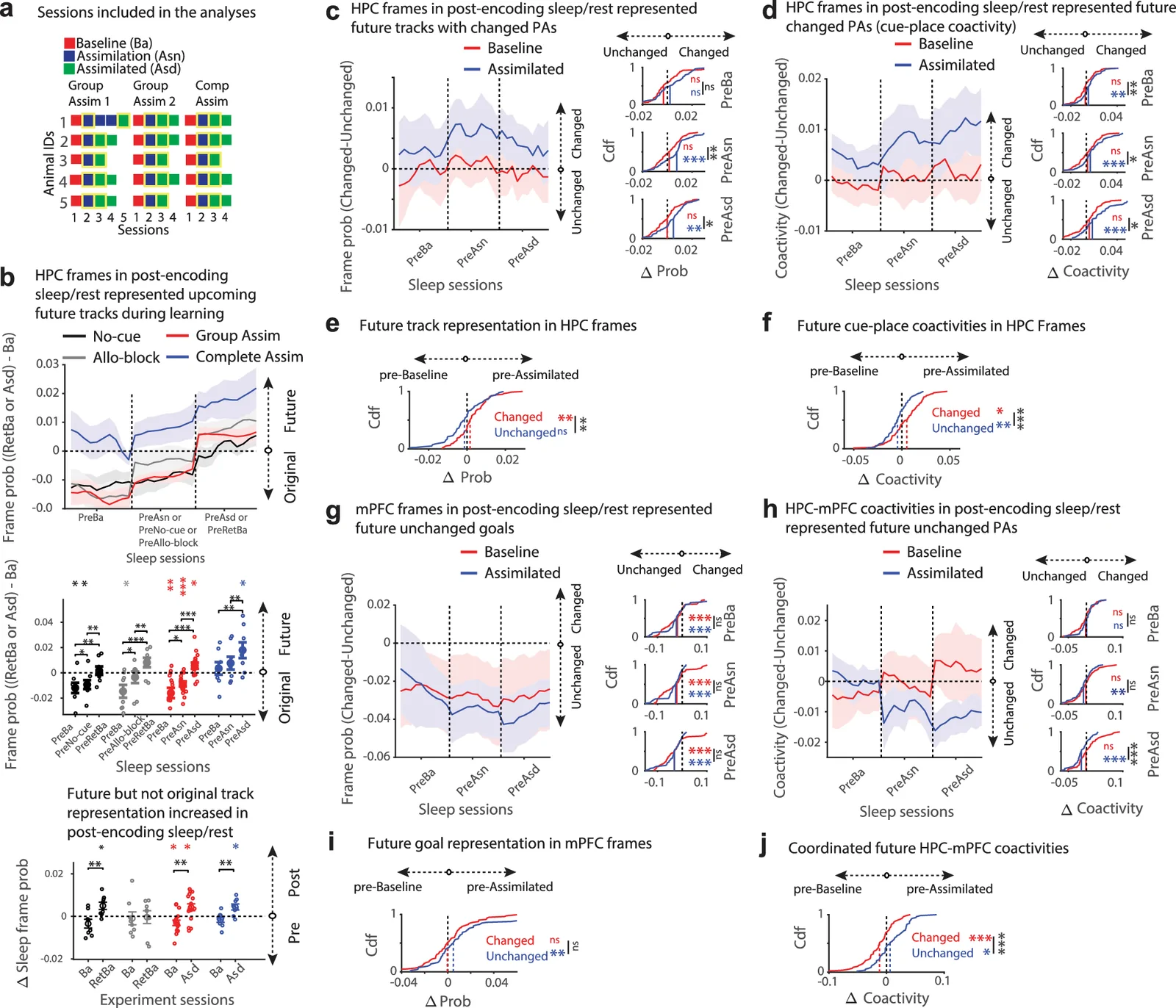
Post-encoding HPC-mPFC network reconfiguration during sleep/rest guided rapid associative learning. **a** First assimilation and assimilated sessions (shown in yellow borders) and immediately preceding sleep/rest sessions were included in the analyses. **b** Increased representation of upcoming assimilated-session trajectories during pre-assimilated sleep/rest in group and complete assimilation learning days compared to other sleep/rest sessions and control days. Biases toward assimilated or return-to-baseline sessions compared to baseline session trajectories were computed on their average decoding probability during HPC sleep/rest frames across all sleep/rest sessions (top, sleep/rest divided into 10 equal time-bins) and compared (middle) across all experimental days. Changes in frame probabilities from pre-assimilation to pre-assimilated sleep/rest were compared between assimilated and baseline session trajectories (bottom). Corresponding sessions were compared with the corresponding sleep/rest during control days (bottom). Two-tailed one-sample t-tests for data vs. 0 and two-tailed paired t-tests for comparison between groups, *N* = 8 for no-cue, allocentric-block, and complete assimilation, and *N* = 16 for group assimilation experiments. Bias toward tracks of changed PAs in decoding probability (**c**) and cofiring of cue-responsive and place cells (**d**) in HPC frames during different sleep/rest sessions are shown (left) and compared (cdfs, right) for baseline and assimilated session tracks in group assimilation days. Change in decoding probability of HPC sleep/rest frames for upcoming assimilated session tracks (**e**) and cofiring of PAs in the HPC frames (**f**) from pre-baseline to pre-assimilated sleep. Biases toward changed goals in decoding probability of mPFC sleep/rest frames (**g**) and cofiring of mPFC cue- and HPC cue-responsive cells (**h**) during different sleep/rest sessions are shown (left) and compared (cdfs, right) for the baseline and assimilated session goals in group assimilation days. Change in decoding probability of mPFC frames for assimilated session goals (**i**) and cofiring of HPC cue and mPFC cue-responsive cells (**j**) from pre-baseline to pre-assimilated sleep/rest. Two-tailed signed-rank tests for data vs. 0 and between groups (**c**–**j**). *N* = 80 (**c**–**g**, **i**), *N* = 60 (**h**, **j**). Data in (**b**-**d,** **g**-**h**) are shown as mean±s.e.m. Detailed statistics are in Supplementary Data 4. PAs Paired associations, Ba Baseline, RetBa return-to-Baseline, Asn Assimilation, Asd Assimilated, Allo Allocentric. Source data for (**b**–**j**) are provided as a Source Data file. ns, *P* > 0.05, ***P < 0.001, **P < 0.01, *P < 0.05.

While post-encoding offline sleep/rest supports memory consolidation^27,49–51^, inferential learning is also actively supported during sleep/rest and appears enhanced following sleep/rest^32,52–54^. In addition, post-encoding offline sleep/rest binds different contextual task features within a memory episode^32,33,55–59^. Coactivation of associated memories in the online^60,61^ and post-learning offline periods^62–64^ is causally linked to associative memory consolidation^65,66^. Therefore, we hypothesized that during accelerated learning, a process of compositional inference during post-encoding sleep/rest^24,31,37,38,44,67^ could bind the initially temporally-discontiguous cross-modal rule-associated task features (e.g., cue identity at start arm and associated goal-arm location) by temporal co-firing of their neuronal correlates within and across the HPC-mPFC network. This post-encoding offline temporal binding of rule-associated yet discontiguously experienced task features would be supported by a generative offline re-configuration of the HPC network that would facilitate and predict the rapid selection of the upcoming neural correlates of associative learning^24,37,38,40^. Furthermore, HPC-mPFC interaction^30,68,69^ could predictively activate the associative memory to facilitate upcoming rapid assimilation learning. Consequently, we first asked if activity in post-encoding non-REM sleep/rest frames (i.e., during transient increases in peri-ripple multiunit activity) predicted the shifts in neural correlates for the new PAs from baseline (i.e., past associative framework) to the future assimilated (i.e., rapidly reconfigured associative framework) sessions. We decoded the peri-ripple (±0.75 s around time of ripple onset) sleep/rest frames using stacked spatial templates of the 6 rewarded arms or the temporal templates of the 6 goals during baseline and assimilated (or return-to-baseline in control days) sessions combined. Only the first assimilated sessions and the immediately pre-assimilated offline sleep/rest sessions were included in the analyses (Fig. 4a). We calculated the difference in prediction for the corresponding trajectory (or goal) between future assimilated (or return-to-baseline in control days) and baseline sessions for the cumulative sleep/rest frame epochs. The frames were partitioned into 10 equally-sized bins during 3 different sleep/rest sessions: pre-baseline, pre-assimilation, and pre-assimilated (or corresponding sleep sessions in control days). We observed significantly stronger biases in representational difference toward future assimilated vs. baseline session tracks (Fig. 4b, top and middle) and goals (Supplementary Fig. 13a, top and middle) during pre-assimilated sleep/rest across the 3 assimilation learning days. These biases were not observed toward the return-to-baseline sessions in the corresponding sleep in control days (Fig. 4b and Supplementary Fig. 13). This indicates that HPC frames during post-encoding sleep/rest, but not the corresponding post-control session sleep/rest, participated in the re-configuration of the HPC network to represent the future tracks and goals in parallel with the insight-like rapid assimilation of the new PAs. Furthermore, the increased representation of the future tracks in the post-encoding sleep/rest was due to increases in representation of future assimilated *(*vs. baseline), but not baseline, session tracks (Fig. 4b, bottom), from pre-assimilation to pre-assimilated sleep/rest frames in all 3 assimilation learning days. This indicates that the emergence of a predictive HPC network activity during the post-encoding sleep/rest preceded and likely instructed the expression of rapid learning during the subsequent assimilated session. This predictive activity was specific to HPC, since we did not observe such predictive bias for assimilated session goals in the peri-ripple mPFC sleep/rest frames (Supplementary Fig. 13b).

The group assimilation experiment enabled us to track and compare corresponding correlates of novel/changed and congruent/unchanged PAs throughout the sleep/rest sessions. We decoded the HPC sleep/rest frames for each session separately using stacked spatial (for the 6 rewarded arms/tracks) or temporal (for goals) templates as priors in the session of interest. We calculated a difference index between the probabilities for the changed and unchanged tracks or goals. We observed an increased probability bias toward prediction of changed tracks of future assimilated sessions compared to the unchanged ones during both pre-assimilation and pre-assimilated HPC sleep/rest frames (Fig. 4c). Similarly, there was a bias toward increased co-activities of upcoming changed PAs (cue-responsive and place-responsive cell pairs) compared to unchanged PAs of future assimilated sessions in HPC frames during all sleep/rest sessions preceding the assimilated sessions (Fig. 4d). The increase was significant for the cue- and place-cell pairs specific to the same changed PAs compared to unspecific PAs (Supplementary Fig. 13e). Moreover, differential (changed minus unchanged) increase of upcoming, future assimilated session (Fig. 4c, d) in pre-assimilated (compared to pre-baseline) HPC sleep/rest frames were due to the increases in the representation of changed tracks (Fig. 4e) and co-activities of changed PAs (Fig. 4f). We observed that the changed-cue-coding and location coding cell pairs in the assimilation session elevated their coactivities during post-encoding sleep/rest consistent with their consolidation during post-encoding sleep/rest (Supplementary Fig. 13f, g) and previous reports^42,50,51^. In addition, we found that coactivities of the changed PAs in the assimilated session were not elevated during the assimilation session and only appeared elevated during the post-encoding sleep/rest frames followed by their strengthening in the assimilated session (Supplementary Fig. 13g). The increases in encoding-related coactivity reactivation during post-encoding sleep/rest were not greater than those related to the reconfiguration (Supplementary Fig. 13f, g). This suggests that there exists a predictive reconfiguration in addition to the consolidation of newly experienced cue--location coding cell pairs during post-encoding sleep/rest. Therefore, our results indicate that HPC sleep/rest activity elevated the representation of future goal locations and the novel cue-goal location neuronal co-activities (Supplementary Fig. 13g) as part of a *generative network reconfiguration*^24,38^, which could support, via neuroplasticity, the rapid learning and expression of all 3-6 new PAs in the subsequent, assimilated session. While the results are partly consistent with the proposal that in humans (using regional magnetoencephalography) replay compositionally re-structure sequential familiar object memories according to a familiar rule^26,52,70^ our findings have broader implications. First, offline HPC generative reconfiguration during post-encoding sleep/rest likely linked numerous (3–6) new associations across two modalities (flavor-place) simultaneously within a tens-of-milliseconds timeframe, according to a previously learned abstract rule via orderless neuronal co-activity. Second, this associative linking process updated the associative framework and enabled cue presentation to predict the associated goal location during the following assimilated session. Third, the absence of this predictive reconfiguration in the HPC during the sleep/rest following the control-day experiments is consistent with and further emphasizes that activity during these sleep/rest frames supports an insight-like rapid learning process.

The neural mechanisms of how stability in the cortical associative memory framework is maintained while flexibly integrating new memories into the framework during learning are still debated^40,71–74^. To investigate this, we conducted analyses on neuronal activity during peri-ripple sleep/rest mPFC frames and coordination of neuronal ensembles between HPC and mPFC. Our results showed that mPFC frames were significantly biased toward representing the unchanged goals of both baseline and the future assimilated sessions in all sleep/rest sessions (Fig. 4g). Meanwhile, there was no such bias for the baseline unchanged goals in the pre-baseline sleep/rest during the first group assimilation day. The inter-region coordination via co-activation of neural correlates across HPC-mPFC in pre-assimilated sleep/rest (i.e., HPC cue-responsive--mPFC cue-responsive cell pairs, Fig. 4h; HPC place cells--mPFC cue-responsive cell pairs, Supplementary Fig. 13c) was biased toward unchanged PAs of future assimilated sessions. We also observed significantly increased representation of unchanged goals of future assimilated sessions in pre-assimilated (compared to pre-baseline) mPFC sleep/rest frames (Fig. 4i). Similarly, coactivity of neurons responsive to unchanged PAs of assimilated sessions (i.e., HPC cue--mPFC cue-responsive cell pairs, Fig. 4j; HPC place--mPFC cue-responsive cell pairs, Supplementary Fig. 13d) increased during pre-assimilated (compared to pre-baseline) sleep/rest in combined HPC and mPFC ensembles. These results suggest that mPFC sleep/rest frames and mPFC-HPC ensemble co-activity predictively activated future unchanged PAs, which could represent a mechanism for maintaining associative framework stability. Taken together, HPC relayed the stable PAs via coactivation to mPFC, as also observed in isolated mPFC frame readouts, while the novel PAs were incorporated via generative network reconfiguration during HPC sleep/rest frames. Therefore, the HPC-mPFC network could functionally segregate continually reconfiguring memories to avoid interference between novel and congruent memories and enhance network stability during the rapid assimilation process. We hypothesize that this would be followed by a more gradual systems consolidation of novel PA-memories into the mPFC network.

### HPC-coordinated inferential mPFC schema reconfiguration and rapid memory consolidation during sleep

**Fig. 5:**
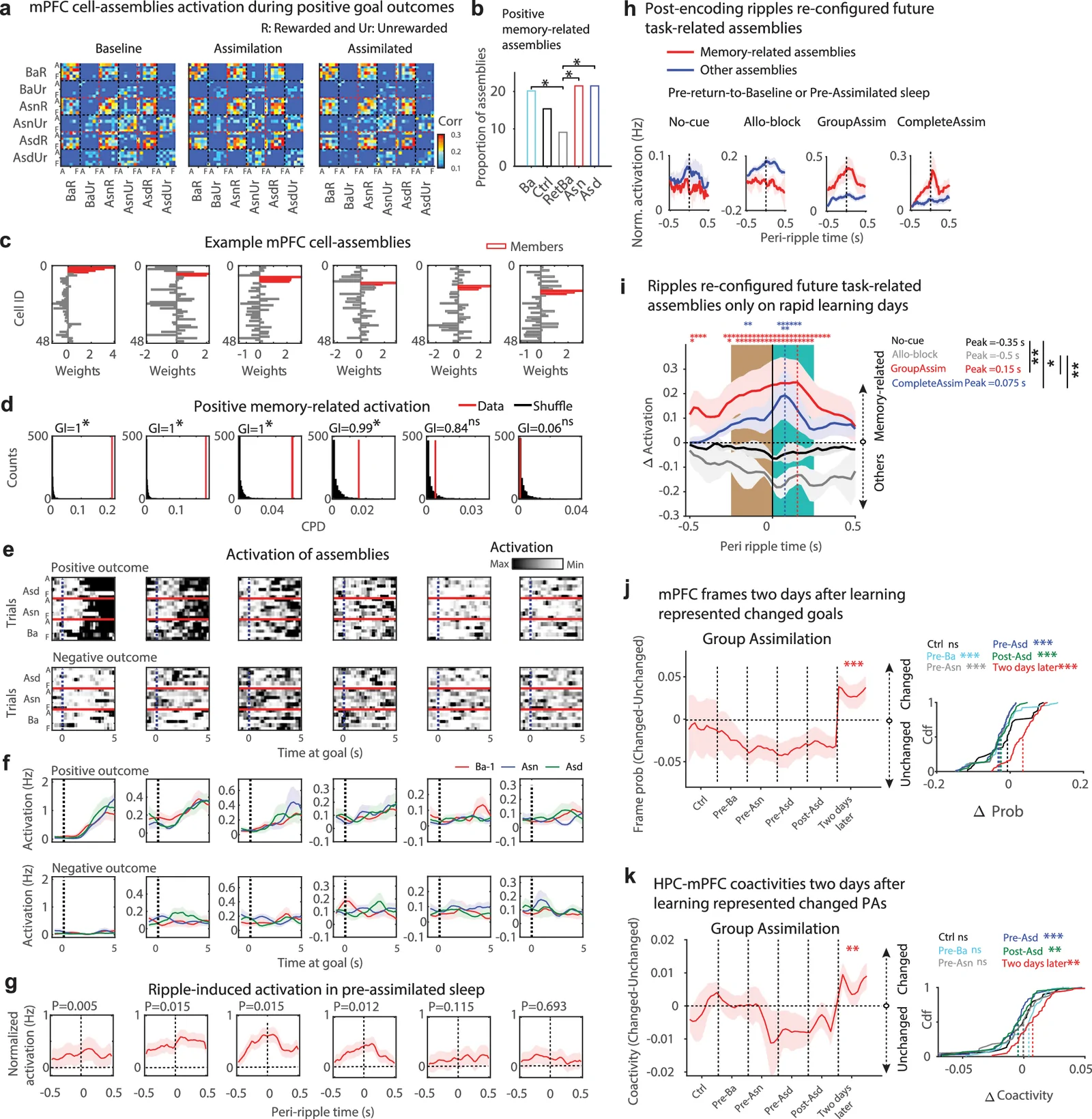
mPFC network predictive reorganization in coordination with HPC ripples during sleep/rest. **a** Example RSMs for activation of awake mPFC cell-assemblies during goal port sampling across sessions in a group assimilation experiment. **b** Comparison of proportions of cell-assemblies detected in different session types that generalize positive outcome-memory across sessions. **c** Example cell-assemblies with participant neurons (red, >1.5 z-score) detected in the assimilated session. **d** Coefficient of partial determination (CPD, red, see Methods) for each cell-assembly compared against shuffle CPDs in black (GI, positive outcome-memory generalization index). Heatmaps showing activation of corresponding mPFC cell-assemblies from (**c**) in different sessions (**e**, goals A-F in rows) and averaged activation (**f**) during positive (top) and negative outcome (bottom). **g** Normalized peri-ripple mPFC cell-assemblies from (**c**) activation during pre-assimilated sleep/rest (P-values on top; two-tailed Rank-sum tests for post- vs. pre-ripple onset, 250 ms shown in colored patches). **h** Peri-ripple activation of memory-related vs. other cell-assemblies, assimilated or return-to-baseline cell-assemblies shown in pre-assimilated or pre-return-to-baseline sleep/rest. **i** Ripple-induced difference in peri-ripple activation (memory-related minus other cell-assemblies) from (**h**). Peak activation times are indicated in legend and by vertical dashed lines. Bias in the decoding of mPFC frames (**j**) and the coactivity of mPFC cue- and HPC place-responsive cell pairs (**k**) towards the unchanged associations in the pre-assimilated sleep/rest was significantly reversed (bottom, cdf) in the pre-baseline sleep/rest two days later. Ctrl (top plots, x-axis label) shows non-significant bias for the baseline session in the first group assimilation experiment in the pre-baseline sleep/rest. **i** two-ta**i**led rank-sum tests (post- vs. pre-ripple onset, 250 ms shown in colored patches compared among experiment days); two-tailed signed-rank tests, *N* = 80 (**j**) and 60 (**k**) for all sleep sessions except *N* = 40 (**j**) and 30 (**k**) for ‘Ctrl’. PAs Paired associations, Ba Baseline, RetBa return-to-Baseline, Asn Assimilation, Asd Assimilated, Allo Allocentric. Errors indicate s.e.m. Detailed statistics are in Supplementary Data 5–7. Source data for (**b**, **i**–**k**) are provided as a Source Data file. ns, *P* > 0.05, ***P < 0.001, **P < 0.01, *P < 0.05.

We asked whether HPC-mPFC interaction during post-encoding pre-assimilated sleep/rest could reconfigure the coordinated cell-assembly^31,75,76^ activity supporting the future, assimilated associative framework to facilitate rapid assimilation learning. On average, mPFC cell-assemblies activated more similarly during a positive outcome at goals across sessions (Fig. 5a). We compared the similarity in the activation of these cell-assemblies during positive outcomes across sessions against the shuffled similarities to derive a positive outcome-memory generalization index (GI, see Methods). A higher proportion of mPFC cell-assemblies active in the assimilated session activated more similarly across baseline, assimilation and assimilated sessions when rats encountered positive outcomes at goals (GI > 0.95) compared to assemblies active in corresponding reversal-to-baseline sessions in control days, when rats did not learn new PAs (Fig. 5b). We displayed example activations of 4 positive memory-related (i.e., GI > 0.95; Figs. 5c) and 2 non-memory-related cell-assemblies (i.e., GI < 0.95; Fig. 5c) detected in the assimilated session during 6 epochs at goals in 3 session types during both positive- and negative-outcome goal visits (Fig. 5d–f and Supplementary Fig. 14).

We next asked whether positive memory-related assemblies in mPFC were coordinated with the HPC during the preceding sleep/rest. We found that the positive memory-related mPFC assemblies active in the assimilated session, but not the ones active in the corresponding return-to-baseline session in control days, had higher HPC ripple-associated activations during the preceding sleep/rest compared with the remaining cell assemblies (Fig. 5g, h, Supplementary Figs. 15a, b and 16a). Normalized post-ripple activation (post- minus pre-ripple, 250 ms epochs each referenced to the ripple onset time) of positive memory-related mPFC assemblies in the assimilated session was significantly higher in the pre-assimilated sleep/rest on all 3 assimilation learning days compared to corresponding assemblies on control days (Fig. 5i, Supplementary Figs. 15a, b and 16b). This suggests that mPFC cell-assemblies of the reconfigured framework that encoded outcome congruencies across sessions were regrouped in coordination with HPC ripples during post-encoding sleep/rest sessions. These assemblies were less numerous and less modulated by ripples in the pre-return-to-baseline sleep/rest in the control experiment days (Fig. 5b, h, i and Supplementary Figs. 15a, b), which indicated they were not simply driven by the receipt of reward or the flavor associated with the reward. This is also consistent with our earlier finding that the HPC ensembles relay continually reconfiguring correlates of congruent PAs through co-activities in the post-encoding sleep/rest. In addition, we found that a significant proportion of the members of positive-memory-related cell-assemblies in assimilated sessions also responded to cues during the assimilated sessions on assimilation days, but not in the corresponding sessions during the control days (Supplementary Fig. 16c). Like cell-assemblies, mPFC cue-responsive cells active in future assimilated sessions had higher ripple-related activations than the non-cue-responsive cells during the preceding pre-assimilated sleep/rest in all 3 assimilation days; this was not the case during the pre-return-to-baseline sleep/rest sessions in control days (Supplementary Figs. 15c and 16d–g). Together, these results suggest that HPC ripple-related activity shaped the post-encoding predictive reconfiguration of outcome congruency memory-representing assemblies and their future PAs correlates in mPFC, implying an HPC role in rapidly shaping congruent memories to produce a stable post-learning framework in mPFC.

The mPFC participates in a protracted process of memory consolidation and recall^77–79^ primarily driven by the slow HPC-mPFC interaction and ‘memory transfer’ over several weeks^80,81^. However, during rapid associative learning, the memory consolidation process was accelerated, and the assimilated, reconfigured associative framework could sustain new rapid learning in the absence of all the original PAs in our task. Moreover, earlier we hypothesized that gradual integration of novel memories into the mPFC network could take place following the rapid assimilation learning process. Indeed, we detected a rapid shift in mPFC activity from primarily representing pre-existing upcoming unchanged goals during pre-assimilated sleep/rest in the group assimilation days to significantly representing the latest changed goals during the pre-baseline sleep/rest, when tested 2 days (~36 h) later (Fig. 5j). Similarly, when we investigated the HPC-mPFC coordination, we detected a rapid shift from coactivation of neuronal ensemble correlates of pre-existing upcoming unchanged PAs in the pre-assimilated sleep/rest to those significantly representing the latest changed PAs in pre-baseline sleep two days after the assimilation experiment. This shift was observed for pairs of HPC place- and mPFC cue-responsive cells (Fig. 5k) as well as HPC and mPFC cue-responsive cells (Supplementary Fig. 16h). Together, these results are consistent with a rapid systems consolidation^82^ of group assimilation learning of new PAs, which was retained in the reconfigured associative framework when tested 2 days after, but not the same day as, encoding. This accelerated consolidation was likely supported by the rapid increase in coordination between reconfigured cell-pair activities of new PAs between mPFC and HPC, which were initially expressed only in HPC during post-encoding sleep/rest session.

### Closed-loop ripple disruption during post-encoding sleep/rest interfered with accelerated learning

**Fig. 6:**
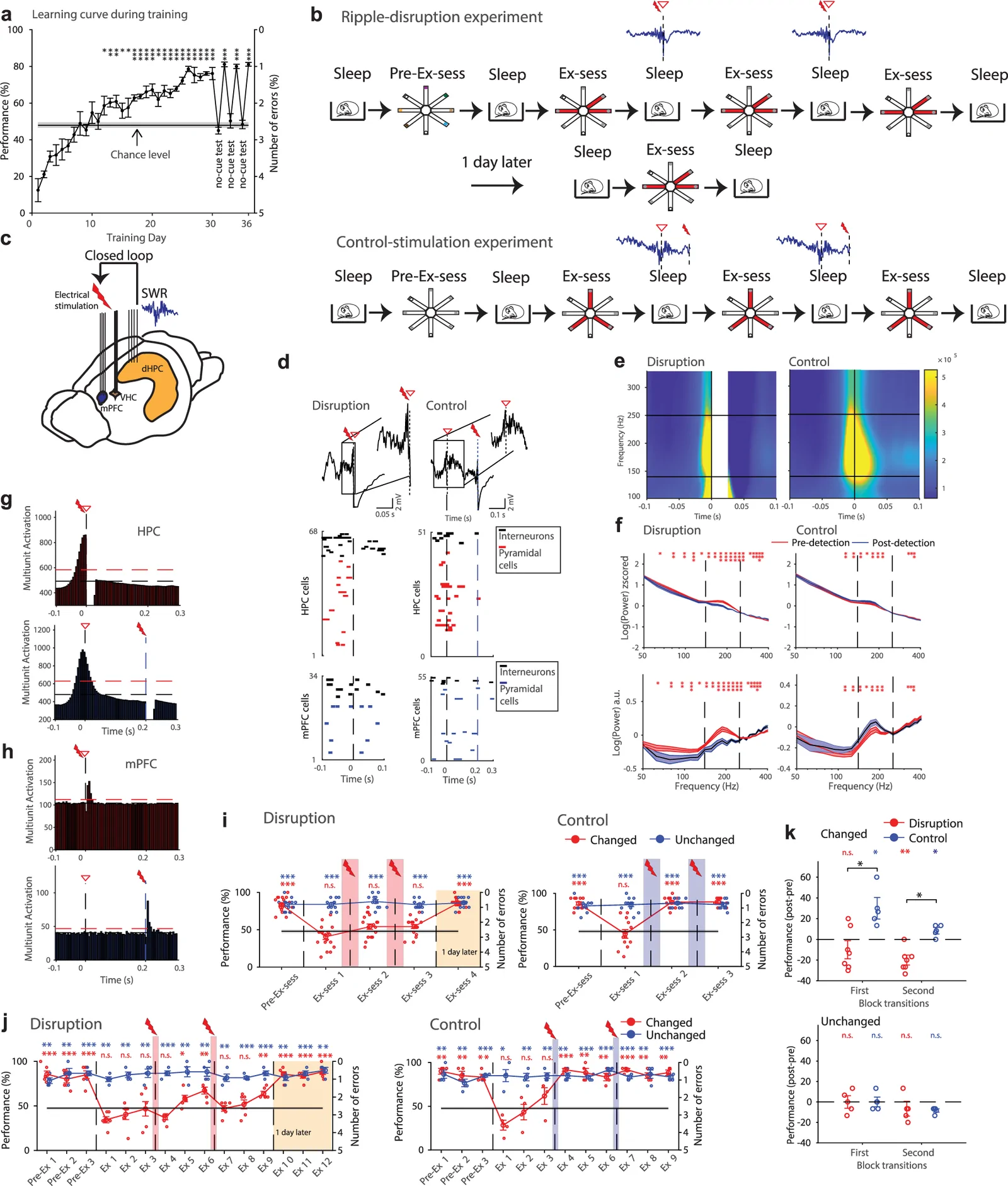
Closed-loop ripple disruption during post-encoding sleep/rest interfered with rapid learning. **a** Learning curve depicting performance (left ordinate) and errors (right ordinate) across training days and no-cue tests for an additional group of rats (*N* = 4). **b** Task protocol for ripple-disruption and control-stimulation experiments. We changed different sets of 3 PAs or all 6 PAs during exposure/encoding sessions as variations of the protocol (see Methods). **c** Closed-loop ripple disruption and recording set-up. **d** Example ripple-disruption (left) and control-stimulation (right, 200 ms delayed) stimulation epochs. Top shows wide-band (1–500 Hz) LFP traces; middle and bottom show HPC and mPFC single-unit spikes, respectively, from pyramidal cells (red/blue) and interneurons (black). Triangle and shock symbol show the time point of ripple detection and stimulation, respectively. **e** Peri-detection wavelet spectrogram during ripple-disruption and control-stimulation epochs. Post-stimulation electrical artifacts obscured measurements for 25 ms during stimulation epochs. **f** Z-scored (top) and residual (bottom) power spectrum measured during pre- vs. post-detection periods (100 ms period/epoch in *N* = 12 and *N* = 8 sleep sessions for disruption and control epochs, respectively). Peri-detection multiunit activation (Hz) for HPC (**g**) and mPFC (**h**). Post-stimulation electrical artifacts obscured spike detection for 20 ms during stimulation epochs. **i** Performance for changed and unchanged PAs during various pre-exposure and exposure sessions (each session had 3 exposures to each PAs) during ripple-disruption (left) and control-stimulation (right) experiments (disruption, *N* = 18 and *N* = 12 for changed and unchanged, respectively; control, *N* = 12 for both changed and unchanged, respectively). **j** Performance for changed and unchanged PAs at single-exposure resolution throughout pre-exposure and exposure sessions during ripple-disruption (left) and control-stimulation (right) experiments. **k** Performance change (post minus pre stimulation exposure block) caused by disruption vs. control stimulations. **j**, **k** disruption, *N* = 6 and *N* = 4 for changed and unchanged, respectively; control, *N* = 4 for both changed and unchanged, respectively. Two-tailed paired t-tests in (**a**, **i**, **j**), two-tailed signed-rank test in (**f**) and one-tailed one-sample t-tests against 0 in (**k**). Ex Exposure. Data are shown as mean±s.e.m.in (**f**–**k**). Source data for (**f**, **i**–**k**) are provided as a Source Data file. ns *P* > 0.05, ***P < 0.001, **P < 0.01, *P < 0.05.

To test a causal link between post-encoding HPC sleep/rest ripple-related neuronal activity and accelerated learning of new PAs in this task, we designed a post-encoding closed-loop ripple disruption paradigm. We trained a new batch of rats (*n* = 4 rats) using the same de novo 6 PAs learning task. These rats showed similar learning curves and similar chance-level performance on the three alternative no-cue test sessions compared to the previous batches of rats (Fig. 6a). After the rats acquired the 6PAs task, we implanted them with the same recording electrode drive over the HPC and mPFC and a bipolar stimulation electrode placed in the right ventral hippocampal commissure (VHC). Following re-training to pre-implantation criteria, we exposed these animals to our 3-6 PAs rapid learning protocol (Fig. 1) and conducted sets of post-encoding sleep/rest ripple disruption and control-stimulation experiments (Fig. 6b) while recording from HPC CA1 and mPFC prelimbic areas. Closed-loop online ripple disruption was achieved through real-time detection of ripple onset, which triggered a single transient (0.3 ms) bipolar stimulation (100-250 µA, optimized for each rat/session ahead of experimental sessions) through a bipolar stimulation electrode placed in VHC (Fig. 6c, d and Supplementary Fig. 17). Animals performed multiple behavioral sessions in the maze, interleaved by sleep/rest sessions in the off-maze sleep box (Fig. 6b) across 3 distinct ripple-disruption and 2 control-stimulation days, as below. We tested rapid learning of new PAs (replacing either sets of 3 or all 6 PAs; see Methods) into the pre-existing associative framework (Fig. 6b). In each experimental day, we conducted three encoding sessions of exposure to the same new PAs (i.e., assimilation-like) that were preceded by a baseline 6-PAs session, all interleaved by sleep/rest sessions. We conducted ripple disruption on the first two post-encoding sleep/rest sessions, each following the exposure sessions to new PAs. We also conducted control-stimulation experiments using the same exposure to the new PAs protocol, where the same ripple detection and stimulation criteria were used, but a delayed VHC stimulation was applied 200 ms post-ripple detection during post-encoding sleep/rest (Fig. 6b–d). Ripple-time stimulation produced the suppression of ripple band power and multiunit activity in HPC (i.e., ripple disruption), and a transient increase in mPFC multiunit activation (Fig. 6d–h). Following the ripple disruption sessions after the first assimilation encoding session, our rats failed to learn the 3–6 new/changed PAs even over two additional assimilation sessions that were each preceded by a ripple disruption during the sleep/rest sessions. However, in parallel, they remembered and performed well on the unchanged PAs in all 3 assimilation sessions (Fig. 6i and Supplementary Fig. 17). In the control-stimulation experiment, which spared sleep/rest ripples and associated neuronal activities, rats learned all PAs rapidly after a single assimilation exposure session (3 trials for each new PA), consistent with an insight-like process. They also performed significantly better than chance on both subsequent exposure sessions (i.e., assimilated-like), each preceded by a control-stimulation sleep/rest session (Fig. 6i and Supplementary Fig. 17). Further dissection of performance at the single block level revealed that ripple disruption prevented the insight-like rapid improvement in performance consistently observed after the first post-encoding sleep/rest during control-stimulation and no-disruption conditions (Figs. 6j and 1j). During the second exposure (i.e., assimilation) session following the first ripple disruption sleep/rest session, rats showed moderate within-session gain in performance for the changed PAs, which was wiped out by the ripple disruption in the following sleep/rest session (Fig. 6j). Both sessions of post-encoding sleep/rest ripple disruption blocked performance improvement (post- minus pre-stimulation trial performance) specifically for the changed PAs. In contrast, our rats showed significant performance gains after the control-stimulation sleep/rest, while performance for the unchanged PAs remained high in all stimulation groups (Fig. 6k). Neither sleep quality nor ripple-detection periods were affected by the ripple disruption stimulation (Supplementary Fig. 17f, g). The effects produced by ripple disruption primarily blocked the accelerated insight-like learning, but did not prevent the slower new learning, since our ripple-disrupted rats showed high performance for both the changed and unchanged PAs the next day after an unstimulated overnight sleep/rest (Fig. 6i, j and Supplementary Fig. 17).

## Discussion

We revealed that accelerated learning via assimilation of multiple novel cross-modal associations into a pre-existing associative mental schema framework was primarily achieved via rapid map-based associative inference and generative reconfiguration within the HPC network, together with conservation of general task rules within the mPFC network. Rapid binding of temporally-discontiguous novel flavor cue-coding and place-coding neurons into temporally-coordinated cell assemblies in the HPC and across HPC-mPFC during offline post-encoding sleep/rest via a generative network reconfiguration process supported subsequent task learning and performance in the maze. The generative network reconfiguration was demonstrably achieved during the sleep/rest epochs immediately preceding increased animal performance in the maze, and blocking ripples in the post-encoding sleep/rest blocked this rapid learning. Therefore, we propose that generative reconfiguration during post-encoding sleep/rest had an active and predictive instrumental role in guiding rapid new associative learning. This offline generative predictive reconfiguration resembles the phenomenon of *generative preplay*^24,37,38,40,67^, here depicting, via temporal binding of the rule-associated temporally-discontiguous new flavor and place coding cells into contiguous co-firing cell-assemblies, the correct new flavor-place associations driving future increases in animal performance in assimilated sessions. We propose that this generative network reconfiguration, matching future expressions of new correct associations (i.e., in assimilated sessions) is critical for guiding insight-like rapid inferential learning, decision-making, and problem-solving, which were all affected following ripple disruption in the preceding sleep/rest session. Importantly, this generative preplay-like reconfiguration is distinct from the classic reactivation/replay phenomena depicting *past* contiguous maze or associative experiences (i.e., of baseline and assimilation sessions), which are critical for memory consolidation. We first demonstrated that our rats acquired de novo the complex 6-flavor cue-place association task over 30 training days/6-8 weeks. Second, the resulting abstract associative framework accelerated simultaneous learning of 3–6 new PAs after only 3 exposures to each PA within a session followed by a sleep/rest session, despite the absence of half to all the original PAs. Since our rats’ behavioral performance for all new PAs was at chance during the first encoding-assimilation session but, given a post-encoding sleep/rest session, improved simultaneously for all new PAs in the subsequent session on the maze (Supplementary Fig. 4), we posit that this accelerated learning has the features of insight-like learning^32,34,35^. In the context of our experimental paradigm, insight-like learning refers to the rapid discovery and application of an existing abstract rule to rapidly form novel, task-related associations between existing elements toward solving the 3–6 distinct new paired-associations task at once. Rapid task-solving via insight has advantages compared to a slower incremental successive learning/solving of the new paired-associations task in a more deliberate, analytic manner.

Our cross-modal 6-PA associative schema rules, which required 6–8 weeks of training to acquire and later accelerated learning of multiple new similar associations, are distinct from memory retrieval of single, innate, episodic-like learning and natural behaviors such as food-caching in chickadee birds^83^ or context-specific associative fear conditioning in rodents^84^. We believe that one key distinction in our paradigm compared to one-shot memories formed and retrieved in birds and in some innate contextual-fear conditioning tasks in rodents is that, aside from being innate behaviors, these tasks are all based on *episodic* experiences (i.e., what happened where and when). The salience of these individual memories, either innately through their wiring in the brain or through the strong fearful nature of emotional responses these produce, makes them peculiarly memorable and retrievable. In addition, most of these rapid learning processes are tested using single associations, not numerous distinct experiences in the same learning session. However, in our paradigm, the complex and concurrent learning of 6 PAs took many weeks, resulting in the formation of a schema of these semantic associations. Learning of the new PAs was then accelerated by using the rules based on this schema. Additionally, the persistent memory of the newly acquired sets of PAs observed during the following 2–4 days’ baseline sessions was consistent with a rapid systems consolidation into long-term memory of the 3-6 new PAs. The original associative framework changed dynamically to recurrently accelerate the rapid assimilation of 3 different sets of 3–6 new PAs across 3 different experiments spanning 7 days. Together, the rapid assimilation learning, accelerated systems consolidation, and the neural framework flexibility demonstrated here fulfill a stricter definition of a mental schema supporting adaptable, non-episodic, and continual accelerated long-term memory formation and retrieval^3,4^.

### Map-based associative inference in the HPC

Our findings suggest that in our task, inferential associative learning occurred not by simply repurposing the neurons encoding the original cues toward encoding the same location-corresponding new cues. Instead, an inferential activation of a spatial schema of associative rules at the common third element (i.e., goal location) played a critical role in rapid learning. Specifically, invariant HPC ensemble activation during new cue-flavored reward delivery at the correct goal location (positive outcome) associatively linked the new cue to the location. The orderless, symmetric nature of this association via co-firing reactivation during post-encoding sleep/rest, together with the generative network reconfiguration for the new flavor-place association, underlies the inferential linking process by which subsequent cue presentation in the start arm activated and predicted the future goal. Therefore, our results support a map-based associative inference model^22,23^, while not detecting evidence for a direct cue feature binding and transfer^18,19^, in guiding the insight-like accelerated learning of 3-6 new PAs simultaneously after one encoding and one offline sleep/rest session. Interestingly, this mechanism remained robust regardless of whether half or all PAs were changed simultaneously. A significantly impaired activation of the associative rule during the no-cue day indicated that the inferential mechanism was driven by outcome association to cues and not the outcome alone. During assimilation learning, the presentation of the new cue at trial initiation activated the associated goal predictive representation (i.e., neural representation of a positive outcome at goal). This predictive process revealed a novel phenomenon of newly learned cross-modal (i.e., flavor-place) associative memory activation in the HPC, which is distinct from spatial trajectory representation. Interestingly, familiar cue presentation at the start arm or at the goal location did not associate with prospective representation of outbound or inbound trajectories to/from the goal location, respectively (Fig. 3o). Therefore, our results have demonstrated how the HPC organized the goal-associated invariant representations across learning and rapidly reconfigured novel associations in the post-encoding sleep/rest. The reconfigured associations were critically activated to guide goal-directed behavior based on inference, devoid of episodic details (e.g., cue and trial identities, overlap in PAs). These results also describe how schema-based learning^8^ and reconsolidation of original memories^85,86^ likely rely on the HPC. Thus, abstraction in the HPC, such as evidence accumulation^87^ and reward values^88,89^, not only support declarative memory and keep the cognitive map updated, but also activate semantic frameworks of prior knowledge during schema-based rapid learning.

### Post-encoding generative reconfiguration in the HPC

Consistent with the sudden improvement in animal performance from assimilation to assimilated sessions, we found that post-encoding pre-assimilated HPC sleep/rest frames (1) generatively reconfigured the future neural correlates of changed PAs and (2) relayed reconfigured neural correlates of unchanged PAs to mPFC. This sleep/rest related generative network reconfiguration in HPC preceding the improved behavioral performance is more likely to support an active role of sleep/rest in insight-like learning and abstract problem-solving compared to a traditional replay of previously experienced serial patterns of activity^27,38,64^. In our design, a cross-modality relational inference with symmetry features supported by an offline generative neuronal ensemble reconfiguration in the HPC during sleep/rest underlies accelerated learning and integration of multiple new associations into a pre-existing associative framework. We propose this rapid learning in our task relied on 2 rules embedded in the associative mental framework: (1) Preservation of the 2 modalities for association (i.e., flavored food reward and spatial maze location) -- i.e., if one new flavor/cue was given, it will be associated with exactly one (goal) location on the maze; (2) If one location was associated with one new flavor/cue, then that one flavor/cue will also be associated with that one location (symmetry inference^11^). These associative inference and generative network reconfiguration processes are critically distinct from other continual learning paradigms, particularly compositional serial inference^26,52,70^ studied using modeling and correlative behavioral timescale regional MEG in humans during waking states and within a single modality. In our paradigm, the HPC CA1 neuronal ensembles underwent abstract associative rule-guided flavor-place-specific generative reconfiguration via cross-modality neuronal binding through millisecond-timescale co-firing that associated previously temporally-discontiguous events during sleep/rest, consistent with insight-like problem solving.

### Post-encoding reconfiguration in the HPC-mPFC network

Recent reports suggest that the mPFC cell-assemblies immediately activate following new learning^30,76,90,91^ and their disruption prevents the new learning^6,9^. Consistently, our results indicate that mPFC cell-assemblies/coactivations related to congruent memories coordinated with HPC sleep/rest ripples as well as neuronal ensembles, and were activated during post-encoding sleep/rest. However, these HPC-coordinated mPFC cell-assembly coactivations were future-oriented reconfigurations, consistent with a recent report of pre-configured cell-assemblies in mPFC that activate before new learning^90^. In contrast, HPC sleep/rest frames activated future-oriented reconfiguration of novel memories during post-encoding session sleep/rest, consistent with the idea that pre-configured ensembles and sequences in HPC support future learning expressed during subsequent post-sleep, awake exploratory sessions^24,37,44^. Together, these results provide a likely mechanism of how mPFC could coordinate the segregation and reconfiguration of novel and congruent memories to avoid interference during HPC-dependent encoding of new memories^92^. Additionally, this mechanism could support the stability in reconfiguring cortical associative frameworks^73,74^ when new associations^72,93^ are flexibly incorporated into it, a form of HPC-neocortical generative reconfiguration. Our findings during the presumed systems memory consolidation phase imply that HPC relayed the novel memories to mPFC, which appeared as an elevated ensemble activity for the novel, recently learned information in the mPFC, consistent with the standard memory consolidation theory^9,65,66,94^. Therefore, we propose that coordinated generative reconfigurations between mPFC and HPC networks accelerate learning by segregating the congruent and novel aspects of learning, followed by standard systems consolidation of novel memories.

Our results demonstrated that the post-encoding reconfiguration of congruent memories in mPFC (i.e., of unchanged PAs) and novel information in HPC (i.e., of changed PAs) were associated with HPC sleep/rest ripples. The absence of ripple-synchronized predictive reconfiguration of the mPFC network during the control experiment days implies that these mechanisms were specific and critical to post-encoding sleep/rest toward learning. This is consistent with the proposed role of ripples in HPC-neocortical encoding of memories during new learning^29,42,43^. However, reports regarding post-encoding HPC ripple disruption vary from relative impairment in spatial learning^42,43^ to the complete disruption of recently acquired spatial memory^95,96^. Our data demonstrated the impact of ripple disruption on an insight-like rapid learning process. In our paradigm, the ripple disruption during the post-encoding phase only impaired the insight-like rapid expression of new learning but spared the slower (i.e., by next day) learning. This was likely because ripple disruption only affected the opportunity for offline predictive reconfiguration of the network, disrupting the rapid expression of post-assimilation reconfigured schema. However, encoding new information during assimilation in post-ripple disruption exploratory sessions and the slower systems consolidation processes appeared relatively unaffected by the ripple disruption.

These results clarify the mechanisms of brain state-dependent network dynamics during rapid schema-based inferential learning within and between HPC-mPFC networks. Our findings underscored a critical functional role for the generatively reconfigured HPC neural structures during sleep/rest^24,25,37,38^ in the predictive HPC and mPFC network reconfiguration that enabled post-encoding rapid memory consolidation^97–100^. The novel memory activation in mPFC and in coordinated mPFC-HPC ensembles during the accelerated systems consolidation suggests that sleep/rest and continuous mPFC-HPC dialog dynamically and actively reorganized the cortical schema by continuously integrating novel associative memories^101,102^. Future studies will investigate how these neuronal processes are affected by ripple disruption during post-encoding sleep/rest, the computations in sleep/rest necessary for the accelerated learning and problem-solving insight, and whether the generative reconfigurations in sleep/rest support other forms of flexible and adaptive learning.

## Methods

### Experimental model and subject details

Eighteen Long-Evans adult male rats with body weight ∼350 g were purchased from Charles River Laboratories USA and used for this study. Animal handling and experimental procedures were approved by the Office of Animal Research Support at Yale University and were performed in agreement with the Animal Welfare Assurance on file with the Office of Laboratory Animal Welfare and the NIH guidelines for ethical treatment of animals.

### Methods details

#### Subjects

Eighteen Long Evans male rats (10-12 weeks old at the beginning of the experiment) were handled and habituated in the maze for 5 days. Twelve of these rats were selected for further training due to their improved behavior during the maze habituation phase. Eleven of them were successfully trained over 6-8 weeks and acquired a complex mental schema of a 6-flavor-place association task. One of the twelve rats dropped out in the middle of the training due to lack of significant improvement in behavioral performance. Of these 11 rats, 7 were implanted with a 32-tetrode drive and were further tested on a set of schema-based rapid assimilation learning paradigms while recording neuronal activity from the HPC and mPFC. Five of these rats participated in the main 5-day neural recording experiments and 2 in the assimilation learning and neural recording with ripple disruption experiments. All rats were housed separately under 12 hours of day/light cycles. Behavioral training started with 7 days of pretraining, during which animals were handled extensively and exposed to an eight-arm radial maze daily in gradually increasing durations to habituate to the maze. After pretraining, rats were kept on a food restriction paradigm, and their body weights were maintained at 90% of free-feeding weight (350 to 400 g). Experiments were carried out over several weeks in the 8-arm radial maze.

#### Maze

The 8-arm radial maze was composed of a regular octagonal center 40 cm wide, connected with eight 90 cm arms, each with a proximal 20 cm long, 10 cm high, 6 cm wide *beam* leading into 70 cm long, 6 cm wide arms placed at equal angles radiating around the center. Two of the arms were designated ‘start arms’ while the other 6 were ‘choice’ rewarded arms. During trials initiated in one start arm, the proximal part of all the choice arms of the maze and of the other start arm were elevated using the beams, such that rats endured an extra energy cost of climbing up on the beams and jumping down to visit each choice arm end where food rewards were placed at correct arm visits.

#### Cue-place association task

A trial began at one start arm with the presentation of a 20 mg food flavored with one cue in a cup placed at the end of the start arm. Approximately 2 to 3 s after the rats consumed the flavored cue, they were manually presented with the vial of the same flavor, which they smelled (odor) and licked (taste) for approximately 5 s. There was an imposed delay period of 3 to 5 s at the start arms before rats were allowed to move to the center of the maze. At the center, rats were allowed to choose and visit as many arms (7 arm options) as they wanted, reaching the arm ends, until they chose the correct flavor-associated arm, where they were rewarded with a 200 mg food pellet of the same flavor as the start-arm cue at the arm end (reinforced with odor from the same-flavor vial). The cues and rewards for each trial were prepared by mixing the unflavored food pellets with the natural extracts of the designated flavors (Bakto flavors) and presented in the cups attached to the start arms and the end of the corresponding arms, respectively. Each training session consisted of 18 such trials divided into 3 blocks. Before the session started, we generated sets of 6 random sequences (one for each trial using random number generator in MATLAB) of non-repeating PAs for each block. If the first trial of the next block had the same PA as the last trial of the previous block, another sequence of the 6 PAs was generated and used. At the end of each block, the maze was sanitized with 70% alcohol spray followed by wiping. The start arms were kept constant for the trials of a block, but were switched every consecutive block to discourage animals from using a habit-based strategy for solving the task. Chocolate, blueberry, coffee, strawberry, cherry, and hazelnut (cues 1 to 6) were used as correct cues to associate with the corresponding reward arms (A to E) throughout the whole duration of the schema-learning training. The 5 rats implanted with recording electrodes were selected from 12 pretrained (habituated) rats, of which the 8 best-performing rats were trained on the cue-place association task of schema acquisition for 30 training days, with 7 rats completing the training successfully. Three no-cue test sessions were conducted on 31st, 33rd and 35th training days, intermittently followed by standard training sessions on 32nd, 34th and 36th days. To compare the overlap in flavor ingredients among the different flavors used in the task we used *flabordb2* database^45^.

#### No-cue sessions

The No-cue sessions, which were designed to test for the reliance of rats on the flavored cues to solve the task, were conducted in the same way as the standard training, except that no flavored cues were presented to the animals at the start arms. The flavors were not present in the cue (i.e., only unflavored 20 mg pellets were used) nor in the vial at the start arms. In no-cue sessions, rats were rewarded in the ascribed correct arm with a flavored food reward, as in the standard session. The correct sequence of 18 PAs for the 3 blocks of the no-cue session was also generated randomly earlier in the day, as for the standard cued sessions. The errors made by chance during the no-cue test sessions were used as the chance level for assessing the learning curves and were not significantly different from the theoretical chance level, which assumed a perfect working memory that avoided entering the already visited arms in the trial: 50% of 5 possible errors/trial, i.e., a mean of 2.5 errors.

#### Assessment of strategy

We quantified the different strategies that rats could learn to use in the training period, given the rules of cue-place association and non-repeating PAs within a block included in the task design. We quantified the inferred strategy based on association memory as the improvement in performance from the experimental chance level observed in no-cue session (which was experimentally identical on all aspects except no cues were provided at start arms), and the one based on memory of rewarded arms (reward-history memory) as the improvement (i.e., reduction in number of errors) in the last 2 trials compared to the first 2 trials in a block. Additionally, we correlated the trial numbers and the performance in those trials within a block to test the improvement within block as an alternative measure of reward-history memory (Supplementary Fig.1).

#### Surgery and post-surgery training

Rats that made less than 1.5 errors per trial on three consecutive days during their training period of 30 days (7 out of 8 rats for the main recording experiment) were selected for surgical implantation of the electrode arrays. Irrespective of when they achieved the criteria, they were trained for a minimum of 30 days before going through *no-cue* sessions on alternate days. Recording experiments were conducted on 5 of these rats that completed successful recovery and post-surgery training. Bilateral craniotomies above the hippocampus (HPC) area CA1 centered at 2 mm lateral and –3.5 mm posterior to the bregma, and a single craniotomy above the right medial prefrontal cortex (mPFC) centered at 0.5 mm lateral and 3 mm anterior to the bregma were created for electrode implantation. Surgeries were conducted under general anesthesia using 1-1.5% Isoflurane and analgesia was provided using subcutaneous Meloxicam administered during surgery. Four rats were implanted with 22 (11 + 11) tetrodes at a depth of 1.5 mm above bilateral CA1 and 10 tetrodes at a depth of 2.0 mm above the prelimbic cortex. One rat was implanted with 32 tetrodes 1.5 mm above bilateral CA1 and a 64-channel, 6-shank silicon ‘decatrode’ probe (Neuronexus, Inc.) into the prelimbic cortex.

An additional 4 rats were trained in the task as above. Of these 4 rats, 2 participated in the closed-loop ripple disruption experiment, in which the recording experiments were conducted after the rats achieved the learning criteria explained earlier. These rats were also implanted with 22 (11 + 11) tetrodes at the depth of 1.5 mm above bilateral CA1 and 10 tetrodes at the depth of 2.0 mm above the prelimbic cortex, like the first batch of rats. Additionally, a pair of bipolar stimulation electrodes was implanted 3.8 mm deep, 1 mm lateral and 1.3 mm posterior to the bregma, targeting the ventral hippocampal commissure (VHC). Two screws implanted through the skull posterior to lambda over the cerebellum were used as reference and ground. The implanted tetrodes were independently movable by an array of microdrives, part of a 3D-printed hyperdrive. Subcutaneous Meloxicam was administered twice daily for 3 days after the surgery. Post-surgery training started 7 days after the surgery for electrode implantation using the standard cue-place association task (PAs: 1A-6F). Tetrodes were lowered 50-100 micrometers/day until they reached the desired recording locations during post-surgical training. Rats graduated to the recording experiments once they made fewer than 1.5 errors in 3 consecutive post-surgical training sessions.

#### Electrophysiology and data acquisition

A Neuralynx digital data acquisition system was used for recording raw signals at 30 kHz followed by digitization of the signals filtered between 1 Hz to 6 kHz. Spikes were detected after high-pass filtering the recorded signals above 0.6 kHz when their amplitude exceeded a 50 µV threshold. Fine adjustments in tetrode positions were made up to the day prior to the recording day to maximize the number of single units. The rat’s position was recorded by an overhead camera using two LEDs attached to the headstage. Manual clustering package XClust3 was used to cluster and isolate single units offline^44^. Only units with clear refractory periods based on autocorrelation and stability over time were selected. Spike cluster quality L-ratio and Isolation distance values were calculated to ensure the clusters were clearly separable (Supplementary Table 1). Putative interneurons were identified^44^ based on firing rates (>5 Hz), auto-correlograms and/or spike width and excluded from these analyses. One out of 5 rats was excluded from further single-unit and ensemble data analysis of either HPC or mPFC due to low counts of single units (see Supplementary Table 1). After completion of all experiments, the rats were perfused transcardially under general anesthesia with 0.9% saline followed by 4% paraformaldehyde, and their brains were harvested, sectioned, and Nissl-stained for confirming electrodes locations.

#### Recording neural activity during behavioral experiments

Following schema acquisition, rats underwent 5 days of multi-session control and assimilation into schema experiments, where sessions of behavior in the maze alternated with ~1h-long sleep/rest sessions in a sleep box in the same room. All recording days began with a sleep/rest session followed by a baseline session (standard PA task). On Day1, one no-cue session was conducted, as on days 31, 33, and 35 of schema training, preceded and followed by baseline sessions alternating with sleep/rest sessions. On Day 2, after a baseline and a sleep/rest session, in the group assimilation session, 3 of the original flavors were substituted by 3 novel flavors: blueberry by rose, coffee by caramel, and cherry by almond, followed by a sleep/rest session. An additional session with the new PAs was conducted in 4 out of 5 animals after the criteria were achieved. On *Days 2, 3, and 5*, the assimilation session and following sleep/rest session were repeated in the day until the rats demonstrated >70% *performance for the new PAs*. On Day 3, after a baseline session, the 3 remaining original flavors were substituted with 3 novel flavors: chocolate by vanilla, strawberry by coconut, and hazelnut by banana, followed by a sleep/rest session. An additional session with the new PAs was conducted in 3 out of 5 animals after the criteria were achieved. On Day 4, after a baseline session of 6 PAs (3 introduced on Day 2 and 3 on Day 3), the allocentric visual cues on the walls surrounding the maze were completely blocked using opaque black curtains, followed by a baseline session with allocentric cues uncovered; all sessions on the maze alternated with sessions of sleep/rest in the sleep box. On Day 5, after a baseline session, all 6 flavors were substituted by a set of 6 new flavors: passionfruit, pomegranate, plum, apple, pistachio, and cardamom. Followed by an hour-long sleep/rest session, an additional session with the new PAs was conducted in 4 out of 5 animals after the criterion was achieved.

#### Linearization and firing rates

For spatial data analysis, animal trajectory data were linearized for each arm by projecting the 2D animal position and occupancy onto an average linear trajectory on the arm. This was done for each recording day separately. Spatial analyses were confined to the periods when animals were moving (velocity >4 cm/s). Firing rates and occupancy in 1 cm bins were smoothed with a Gaussian kernel of 3 SD. Place maps were generated by dividing binned firing rates by the binned occupancy, separately for each direction of movement. Time points for lap start, finish, and the cue presentation events were manually extracted from videos using a custom script written in MATLAB. Spikes from 1 s before to 5 s after the events (cue port entry and goal port entry) were binned at 250 ms to calculate the event-related neuronal firing rates.

#### Cue-, place-, and outcome-responsive neurons

To identify cue-responsive neurons in each session, for each neuron, we conducted linear regression using the visit-by-visit event-related mean firing rate as the dependent variable, and the presence or absence of the cue, the location of the event (i.e., arm IDs), and their interaction as different factors. The neurons that showed significant main effects (*P* < 0.05) for the presence of a cue were identified as cue-responsive neurons for the session. An additional independent factor, session identity, was included in the analysis to identify neurons that were modulated by a cue consistently in the sessions in which the corresponding cue was presented. Analyses were performed separately for each of the cues used in the session. Similarly, to identify place responsive neurons, for each arm in each direction, we calculated the neuronal firing rates in 5 different spatial bins (14 cm each). We conducted linear regression analysis using the visit-by-visit firing rate (in periods with velocity exceeding 4 cm/s) for each bin as the dependent variable, the place (bin id), the presence or absence of reward, their interaction as different factors, and the movement velocity as a covariate. The neurons that showed significant main effects (*P* < 0.05) for place were identified as place-responsive neurons for the arm. To quantify reward responsiveness in neurons at goal ports, for each neuron, we conducted linear regression using the visit-by-visit averaged peri-event firing rate at goals as the dependent variable, and the positive or negative reward, the location of the event (i.e., arm IDs) and their interaction as different factors, velocity, position (linear and lateral) as well as theta power as covariates. To quantify the single-unit responsiveness for the session, coefficients of partial determination (CPDs) for each factor were determined by comparing the reduced model against the full model. Furthermore, to determine the significance of the ensemble responsiveness, we generated 500 shuffled CPDs by conducting similar linear regression by shuffling the firing rates across visits while keeping the factors the same. Similar analyses were conducted on the spatial firing rates in the inbound and outbound periods of the arm visits to analyze single-unit responsiveness during these periods to the outcome.

#### Analyses of invariance across sessions

The analyses of across-session representational similarities were conducted on two domains, spatial (outbound and inbound movement trajectories) and temporal (goal port sampling durations). In these analyses, only the first assimilation and assimilated sessions were included in the *assimilation* and *assimilated* sessions of each day and each rat. For spatial and temporal cases, visit-by-visit firing rates were averaged for each spatial bin (1 cm) and temporal bin (250 ms), respectively, during positive and negative outcome visits (outcome types) to each arm separately. For every bin, a matrix of firing rates organized by neuron x trial types (2 outcome types for each arm, resulting in 12 trial types in a session) was created by stacking firing rates from all putative pyramidal neurons from all rats during similar trial types in the corresponding sessions. To quantify the results, for each bin, representational similarity matrices (RSMs) were obtained by correlating (Pearson’s) population vectors within and across the trial types among different session types. In the display of the RSM heatmaps, average correlation values across all spatial or time bins were shown. To quantify the variance across session types explained by the similarity in trial types, we first removed the diagonals and within-session correlation values. For each bin (temporal or spatial), we conducted linear regression by using the across-session correlation values as the dependent variables and outcome types (same vs. different), arm IDs (specific or unspecific), and their interactions as different factors, and the session ID, correlation in velocity, position (lateral and/or linear), and theta power in those bins as covariates. Coefficients of partial determination (CPDs) were obtained by comparing the full model against the reduced models for each factor. The proportion of CPD values obtained from the 500 shuffles (correlation values shuffled across trial types while keeping the factors the same) that were greater than the actual CPD value was taken as the P-value. To determine the significance of the difference among brain areas (HPC vs. mPFC), the actual difference in CPD value was compared against the distribution of the difference in the CPDs obtained from the shuffles of the respective brain areas. To compare the strength of different factors in explaining invariant neuronal representations across days, because we obtained RSMs by stacking the neural population vectors across all animals, we directly tested the difference in the distribution of CPD values (one CPD value per bin per factor for each experiment day) obtained throughout the temporal or spatial bins. Block-wise RSM analyses were conducted in a similar manner, by only including the trials from the block of interest in the manipulation sessions (*No-cue*/*Allo-block* session on control days and *Assimilation* sessions on assimilation experiment days) in the analyses. Only correlation values in the RSM involving *Asn/No-cue/Allo-block* sessions were included from the across-session portions of the RSMs in the block-wise representational similarity analyses.

#### Bayesian decoding of neural activity

Population activity from all putative pyramidal neurons was calculated in the periods of immobility (<1 cm/s velocity) during sleep/rest using 5 ms time bins. Frames of multiunit activity were detected as periods of instantaneous population activity exceeding 2 standard deviations (SD), lasting between 30 ms to 800 ms and containing at least 5 distinct neurons for HPC frames and 3 distinct neurons for mPFC frames. Only frames occurring within close proximity (<750 ms) to HPC ripple onsets (crossing 1 SD to detect onset and 3 SD to detect peak above mean in the ripple band, i.e., 140 to 250 Hz) were included. To identify the periods of NREM sleep and awake rest, we calculated a reconstructed EMG by calculating averaged correlations of high-frequency (300 to 600 Hz) LFP power among the signals obtained from 5 distinct electrodes, as well as theta/delta ratios. The sleep/rest periods when both theta/delta ratio and EMG were below their respective means were classified as NREM. Similarly, the periods when the theta/delta ratio was below mean and EMG was above the mean were classified as awake rest. In summary, frames that occurred in proximity to ripples during both NREM sleep and awake rest sleep periods were included in the frame analyses. Bayesian decoding was conducted either using goal templates created using peri-goal sampling firing rates (250 ms time bins, -1 to 5 s periods) or spatial firing templates in each direction of movement created using 1 cm spatial bins. Briefly, memoryless Bayesian algorithm^44,47,48^ was used to obtain posterior probabilities for each time bin in the frames as:

1 $${Prob}\left({pos}/{time},{spike}\right)=\left({\prod }_{i=1}^{n}{f}_{i}{\left({pos}/{time}\right)}^{{{spike}}_{i}}\right){e}^{-\tau {\sum }_{i=1}^{n}{f}_{i}\left({pos}/{time}\right)}$$

where ${f}_{i}\left({pos}/{time}\right)$ is the firing rate of the i^th^ cell at position ${pos}$ or time ${time}$ in the template, and ${{spike}}_{i}$ refers to the number of spikes from i^th^ cell in the τ time window of 20 ms in each frame. We normalized the posterior probabilities ${Prob}\left({pos}/{time|spike}\right)$ using:

2 $${Prob}\left({pos}/{time|spike}\right)=\frac{{{\rm{Prob}}}\left({pos}/{time}|{spike}\right)}{{\sum }_{i=1}^{{p}_{n}}{Prob}\left({{pos}}_{i}/{{time}}_{i}{|spike}\right)}$$

where ${p}_{n}$ refers to the number of spatial or temporal bins being decoded in a time bin within a frame. The decoded bin with the maximum posterior probability ${Prob}$ was considered the decoded position or time of the animal in the respective time bin in the frame. The decoding errors were calculated by finding the distance between the decoded location/time from the bin of maximum posterior probability and the actual location/time the rat was at in that bin, by decoding the instantaneous firing rates of the neuronal ensembles during the trial events. A similar approach was used to decode the awake state peri-cue sampling epochs (50 ms bins) using the stacked templates created from the temporally binned ensemble firing rates (250 ms bins) during the 6 goal periods, and the predictions for upcoming goals were compared against the shuffles. The decoding error algorithm was validated prior to the analysis by comparing the instantaneous prediction error against the prediction errors (distances of predicted bin from the actual bin) in the shuffles produced by circularly shifting the posterior probabilities among the spatial/time bins 500 times. Similarly, the decoding of HPC place cell ensemble activities during the peri-cue sampling epoch used concatenated spatial trajectories of the 6 arms (1 cm bins, by direction outbound/inbound), and predictions for the upcoming arm were compared against the shuffles. Decoded probability of a goal (or spatial trajectory) during peri-cue sampling was tested against 500 circularly shuffled probabilities among 6 concurrently decoded goals (or spatial trajectories).

#### Awake state LFP analyses

Local field potential (LFP) signals were extracted from one HPC and one mPFC channel and down-sampled to 2 KHz. HPC LFP power spectral density was calculated using the Welch method in MATLAB using the “pwelch”. To compare the HPC brain state of rats during the cue-sampling periods, the HPC LFP signal was band-pass filtered between 6–10 Hz for theta and 140–250 Hz for ripple frequency ranges, and its amplitude was squared to obtain the theta and ripple band power during the 6 s peri-event periods at cue sampling, goal sampling, and in run periods (the latter starting at 10 s after cue-sampling time).

#### Neuronal co-activity analysis

To calculate neuronal co-activity in the frames in HPC and mPFC cortex (*N* = 4 rats for each area), the spike counts of n cells in the pairs were binned into 20 ms time bins for each frame. The time-binned spikes from all frames were then stacked and z-scored (total m time bins from all frames) to obtain an n x m matrix. To calculate the co-activity between HPC and mPFC cell pairs in 3 rats, the sleep/rest activities during immobility (<1 cm/s velocity) were binned into 200 ms time bins, and only time bins with at least 2 distinct neurons from each area were stacked and z-scored to obtain the n x m matrix. Only periods of NREM sleep and awake rest (described above) were used for these calculations. The obtained n x m matrix was then multiplied by the transposed matrix to obtain an n x n matrix, and the resultant matrix was divided by the number of cells (n) to obtain the correlation matrix for all cell pairs. The correlation value for a cell pair was then obtained from the correlation matrix as their co-activity.

#### Sleep/rest analyses

To test whether the ripple-related multiunit frames during sleep/rest predicted the shifts from the neural correlates of the *baseline* (original/preceding schema) to the *assimilated* (reconfigured schema) sessions, we decoded the frames using two different types of neural templates. In all sleep/rest data analyses, only the first assimilated session was included as the assimilated session, and the sleep/rest session immediately preceding the first assimilated session was considered pre-assimilated sleep/rest. First, we decoded the frames using the templates created by stacking the average firing rates in the spatial bins for the 6 rewarded arms during the baseline session together with the assimilated session separately by the 2 directions. Second, we decoded the frames using the templates created by stacking average firing rates in the temporal bins at the 6 goal ports in the baseline and the assimilated sessions together. We calculated the difference in the predictions for the corresponding trajectory (or the goal) between the assimilated and the baseline session for every sleep/rest frame in 3 different types of sleep sessions. To quantify whether the changes in the correlates in the post-encoding sleep/rest frames were due to changes in representation of assimilated or baseline sessions tracks (or goals), we repeated the similar analyses but calculated the increases in the decoding probabilities for assimilated and baseline sessions tracks (or goals) from the pre-assimilation to the pre-assimilated sleep/rest sessions, and normalized the results by the changes for the corresponding tracks (or goals) from pre-baseline to pre-assimilation sleep/rest sessions. For comparison, we conducted the same procedure on the control days, replacing the sleep/rest and the awake sessions with the corresponding control sessions of the control days. Next, we quantified the bias in the hippocampal frames toward the upcoming changed vs. the unchanged PAs-associated tracks and goals. In the group assimilation experiment, we decoded the sleep/rest frames for each session separately by using the templates created by stacking the average firing rates in the spatial bins (or the temporal bins for goal decoding) for the 6 rewarded arms (or goals) in the session of interest. Then, we calculated the difference by subtracting the probabilities for the unchanged tracks (or goals) from the probabilities of the changed ones for each session of interest. To quantify the changes in the correlates in the post-encoding sleep/rest frames, we repeated the similar analyses but calculated the increases in the decoding probabilities for changed and unchanged tracks (or goals) of assimilated session from the pre-baseline sleep/rest to the pre-assimilated sleep/rest, and normalized the results by the changes in the decoding probabilities for the corresponding baseline session tracks (or goals). For comparison, values were averaged for each of the 10 equally sized bins in the sleep/rest. Similar methods were used for comparing the co-activation of different neural correlates, by calculating co-activation (described above) of cell pairs of interest and getting coactivity measure in 10 equally sized time bins in the sleep/rest sessions of interest. In the calculation of coactivity index difference between correlates of changed and unchanged PAs, pairs containing overlapping cells were excluded.

#### Cell assemblies and their activation

To calculate cell assemblies from n number of cells, we first obtained the correlation matrix n x n from the awake state time periods by binning the spikes into an n x m 25 ms time binned matrix, z-scoring, and multiplying it with the transpose of the same n x m matrix. The cell assemblies were calculated using a PCA-based method^31,76^. Briefly, eigenvalues were obtained for the correlation matrix, and eigenvectors were calculated using PCA. The eigenvectors lying within the maximum permitted eigenvalues were considered significant. Each significant eigenvector was then considered a cell assembly, with the eigenvector values as the weight of each cell in the cell assembly. To calculate the strength of cell assembly activations, we computed a projection matrix Q by taking the outer product of the cell assembly weight vector. The diagonal in the Q matrix was replaced with zeros. Activation was then computed as the quadratic multiplication of the Q matrix with the instantaneous normalized firing rates.

$${A\left(\tau \right)={Ifr}\left(\tau \right)}^{T}*Q*{Ifr}(\tau )$$

where $A\left(\tau \right)$ is the activation in each time bin τ, ${Ifr}\left(\tau \right)$ is the normalized instantaneous firing rate vector at time bin τ and Q is the projection matrix. Only activations crossing a threshold of 3 (arbitrary units) were considered significant activations.

#### mPFC cell-assemblies

To study the ensemble-level organization of mPFC neurons, we calculated the cell-assemblies in the awake experimental sessions in 4 rats. To isolate the patterns in the similarities of the cell-assembly activations across sessions, during goal port visits, we conducted the representational similarity analysis by correlating the cell-assemblies’ activation in the 6 different goal sampling durations (6 s at 6 goals divided into 25 ms time bins) for both the positive and negative outcome visits across the sessions. Next, we quantified the proportion of the cell assemblies that activated more similarly in the positive outcome goal visits across sessions. For this, we conducted linear regression by using the across-session correlation values as the dependent variables and outcome types (positive types vs. all other types), arm IDs, and their interactions as different factors. CPDs were obtained by comparing the full model against the reduced models for each factor. The proportion of CPD values obtained from 1000 shuffles (correlation values shuffled across trial types while keeping the factors the same) smaller than the actual CPD value was taken as the positive outcome-memory generalization index (GI), and assemblies with GI > 0.95 were considered significant. The participation of cue-responsive cells as members (>1.5 z-score) was compared against 300 cell ID shuffles to determine the significance of participation in the mPFC assemblies.

#### Ripple-related activation

To compare the co-ordination of the significant (positive memory-related) mPFC assemblies (or cue-responsive cells) with HPC ripples between different experiment days, we first obtained Δ activations by obtaining shuffle-normalized (1000 shuffle sleeps with randomized ripple times) activations of significant assemblies (or z-scored firing rates of significant cue-responsive cells) and subtracting the average shuffle-normalized activation (or z-scored firing rates) of the non-significant ones. We then compared the ripple-related difference in the peri-ripple activations (post minus pre ripple, 250 ms durations) between experiment days.

#### Recording and stimulation in closed-loop ripple disruption experiments

Real-time LFP amplitudes on one channel located in CA1 of HPC were band-pass filtered (100–250 Hz). The amplitude in the ripple range was automatically monitored, and every event crossing a set threshold for 10 ms triggered a single pulse biphasic stimulation of 0.3 ms through the bipolar implanted stimulating electrodes in VHC. The set threshold was predetermined during the monitoring phase before the pre-baseline sleep/rest session. The stimulation current (100 – 250 µA) was predetermined for each rat to produce roughly 100 ms of silencing in the HPC multiunit activation. Every detection was followed by 250 ms of latent period in which no further detection was made, capping the maximum stimulation to 4 Hz. The same protocol was applied in the control-stimulation condition, in which stimulation was triggered after 200 ms of post-ripple initiation delay, to match the trigger rate but not to influence the ripples. Online detection rate was calculated in the control-stimulation condition as the number of online detected events divided by the number of offline detected events.

#### Ripple-disruption and control-stimulation experiments protocols

Following schema acquisition, 2 rats underwent 3 days of multi-session ripple disruption and 2 days of multi-session control stimulation experiment days while performing rapid-assimilation learning, where sessions of behavior in the maze alternated with 1h-long sleep/rest sessions in a sleep box in the same room. All days of recording began with a sleep/rest session followed by a baseline session (standard PA task). On ripple disruption experiment day, after a baseline and a sleep/rest session, in the exposure session, 3 of the original flavors were substituted with 3 novel flavors, followed by a ripple disruption sleep/rest session. Animals then went through one more exposure session followed by another ripple disruption sleep/rest session. Next, animals performed the last exposure session with a sleep session without the ripple disruption. After an overnight sleep, we conducted another sleep-exposure-sleep session on the next day. For each animal, there were three such experiments: two with the group assimilation paradigm, in which we changed 3 original PAs with the new PAs, and one experiment with the complete assimilation paradigm in which we changed all 6 PAs. For every experiment, the PAs that were successfully assimilated in the previous experiment were used as the standard set of PAs in the baseline task. The control stimulation experiment proceeded in a similar paradigm to the disruption experiment, except that in the corresponding sleep/rest sessions we used a control (delayed) stimulation protocol following the detection of each ripple. Since all our rats assimilated the PAs within 3 normal exposure sessions, we did not conduct additional sessions on the next day. Two such control stimulation experiments were conducted in the group assimilation paradigm, in which we changed different sets of 3 PAs with new PAs. Therefore, we conducted a total of 6 multi-session ripple-disruption experiments and 4 multi-session control-stimulation experiments. Because of the salience factor, the first exposure to the paradigm of changing PAs could be more difficult for the rats than the repeated sessions following experiments with the same paradigm. To counterbalance the effect of salience, we started our experiments with the ripple-disruption experiment paradigm in the first rat and with the control stimulation experiment paradigm in the second rat. This way, both experimental paradigms were interspersed in the experimental sequence, to randomize the effects of any of these two paradigms on the subsequent experiment.

#### LFP Analysis in ripple disruption experiments

Local field potential (LFP) signals were extracted from one HPC channel and down-sampled to 2 KHz. Peri-detection wavelet spectrograms in ripple disruption experiments were calculated using the continuous wavelet transform method in MATLAB. The measurements were obscured for 25 ms following the stimulation epoch due to electrical artifacts. Peri-detection power spectral density was calculated using the Welch method (*pwelch*) in MATLAB. First, PSDs were log-transformed and z-scored for comparison between pre and post-detection periods (100 ms each). To account for the trend in the power spectrum, PSDs were log-transformed, and a linear regression was fit to the log-log spectrum. The residuals after subtracting the fit were then compared.

#### Statistical tests

The statistical tests used in the specific analyses are mentioned in the figure captions and Supplementary Data. The statistics were produced using custom and inbuilt codes written in MATLAB (MathWorks). Data are presented as mean ± SEM (standard error of the mean) for averages, unless otherwise noted, and the data distributions are shown in most of the analyses. Normality of the data distributions in cases where we used parametric tests was assumed, not formally tested. Comparisons between pairwise measurements of same sample sizes were conducted using two-tailed paired tests such as paired *t-*tests or signed rank tests, while differences in data distributions were tested using two-tailed tests such as Student’s *t-tests* or Rank-sum tests unless otherwise noted.

#### Materials availability

This study did not generate new unique reagents.

### Reporting summary

Further information on research design is available in the Nature Portfolio Reporting Summary linked to this article.

## Supplementary information

Supplementary Information

Description of Additional Supplementary Files

Supplementary Data 1–10

Reporting Summary

Transparent Peer Review file

## Source data

Source Data

## Data Availability

All data needed to evaluate or extend the conclusions in the article are presented in the main article, Supplementary Figs., and tables. Source data are provided with this paper. All data generated in this study have been deposited into the file servers of the Yale University Medical School. The very large size of raw data prohibits their archiving on public servers. The data are available under restricted access behind a firewall, and access can be obtained from the corresponding author of the study. No clinical datasets or genetic data have been generated or used in this study. Source data are provided with this paper.

## Code availability

The custom codes specific to this study that are needed to interpret, verify, and extend the research in the article are uploaded and made available at: https://github.com/GDYlab/GDYlabcode.
