# Supplementary material for: Offline generative network reconfiguration guides insight-like accelerated learning by assimilation into schema in rats
Source: Nat Commun. 2026 Sep 3;17:9694. doi: 10.1038/s41467-026-77318-1 (PMC13562695; doi:10.1038/s41467-026-77318-1)
Supplement: Supplementary file 1 [file 41467_2026_77318_MOESM1_ESM.pdf]

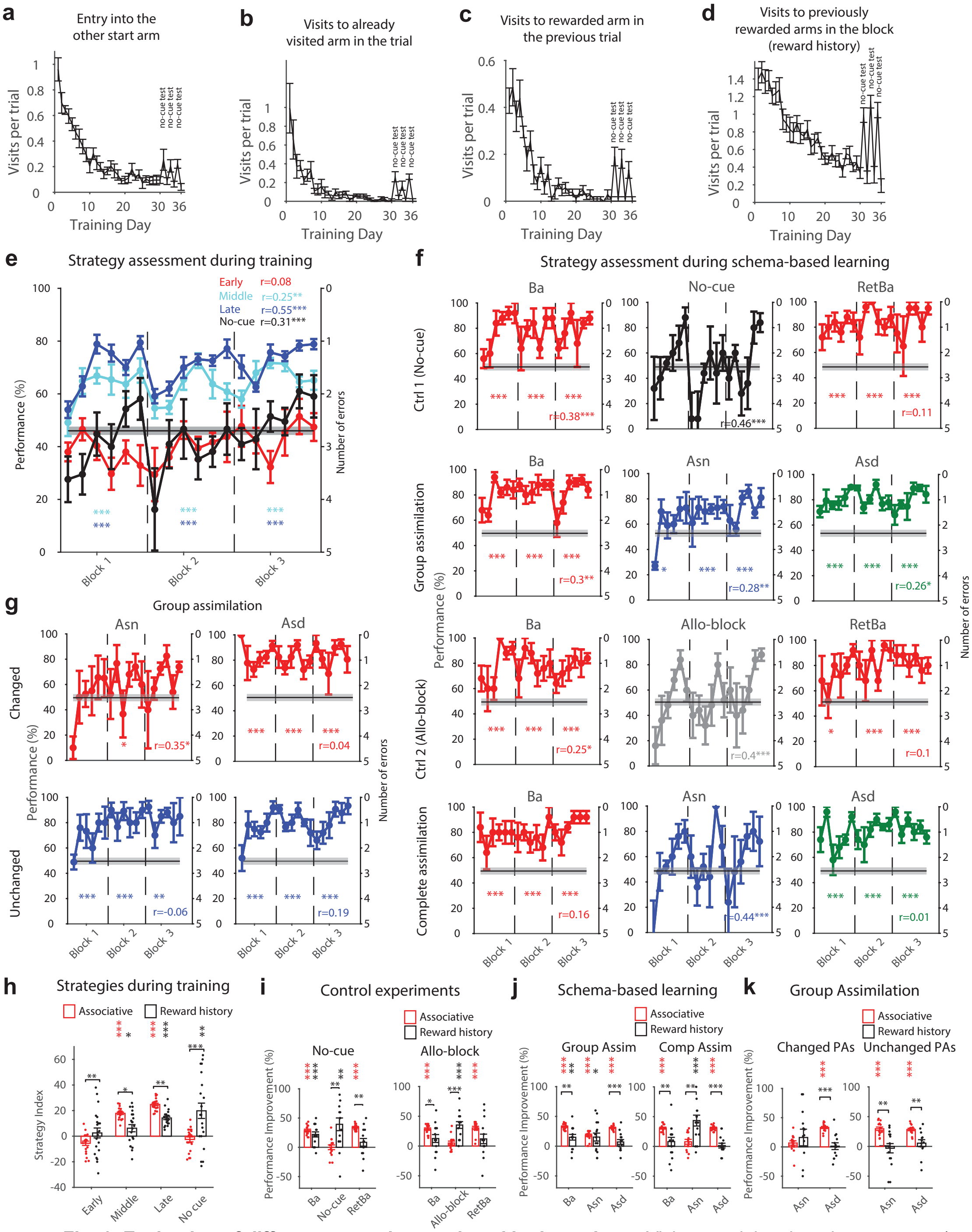

Supplementary Fig. 1: Evaluation of different strategies employed in the task. a-c Visits per trial to the other start arm (**a**, neither cued nor rewarded), the already visited arm in the trial (**b**, within-trial working memory), and the arm rewarded in the previous trial of the block (**c**, previous-trial working memory) during the course of training and in the no-cue sessions. The 3 non-associative task rules (**a-c**) were learned steeply until 10-13th day of training. **d** Visits per trial to previously rewarded arms in a block (reward history memory) depicting the gradual learning of this rule during the course of training and the no-cue sessions. **e** Performance (left ordinate) and errors count (right ordinate) in each trial of 3 blocks during training period, divided into early, middle and late phases (10 sessions/phase each) and no-cue sessions (3 sessions each). In early training period rats did not use any of the two (reward history or associative) strategies. In middle and late training phases, rats used both association memory (indicated by the significantly better performance than chance in all blocks) and reward history memory (indicated by the significant correlation of performance with the trial number in the block and the typical performance reset seen at block transitions). In no-cue test sessions rats only employed reward history memory strategy. **f** Similar analyses as in (**e**) but shown for experiment and control sessions. Rats employed both reward history memory and association memory strategy in baseline sessions (first column) of all experiment days, except complete assimilation in which they used only association memory strategy. In both control sessions (No-cue and Allocentric-block) as well as in assimilation session of complete assimilation day, rats used only reward history memory to solve the task. In assimilation and assimilated sessions of group assimilation days, rats used both strategies. In assimilated session of complete assimilation day and return-to-baseline sessions of both control experiment days rats only used association memory strategy. (**g**) Similar analyses as in (**f**), but shown for changed and unchanged PAs in group assimilation day. In assimilation session of group assimilation experiment, rats used reward history to solve changed PAs, while only used association memory to solve unchanged PAs. In assimilated session they used association memory to solve both changed and unchanged PAs. Two-tailed one sample t-test for testing against chance level and Spearman's correlation for testing relationship between performance and trial numbers in the block in (**e** to **g**). **h** Comparison of learning strategies employed across early, middle and late training phases (10 sessions each; N=21 blocks) and no-cue sessions (3 sessions). (**i-k**) Relative contribution of associative-learning and reward-history strategies to performance improvement in control experiments (**i**) and schema-based learning experiments in combined (**j**) and separate trials with changed and unchanged PAs (**k**) (N=15 blocks). Two-tailed one sample t-tests for data vs. chance levels and two-tailed paired t-tests for comparison between strategies. Data are shown as mean±s.e.m. Detailed statistics are in Supplementary Data 8. Source data are provided as a Source Data file. Abbreviations, Ba: Baseline, RetBa: return-to-Baseline, Asn: Assimilation, Asd: Assimilated, Allo: Allocentric. ***P<0.001, **P<0.01, *P <0.05.

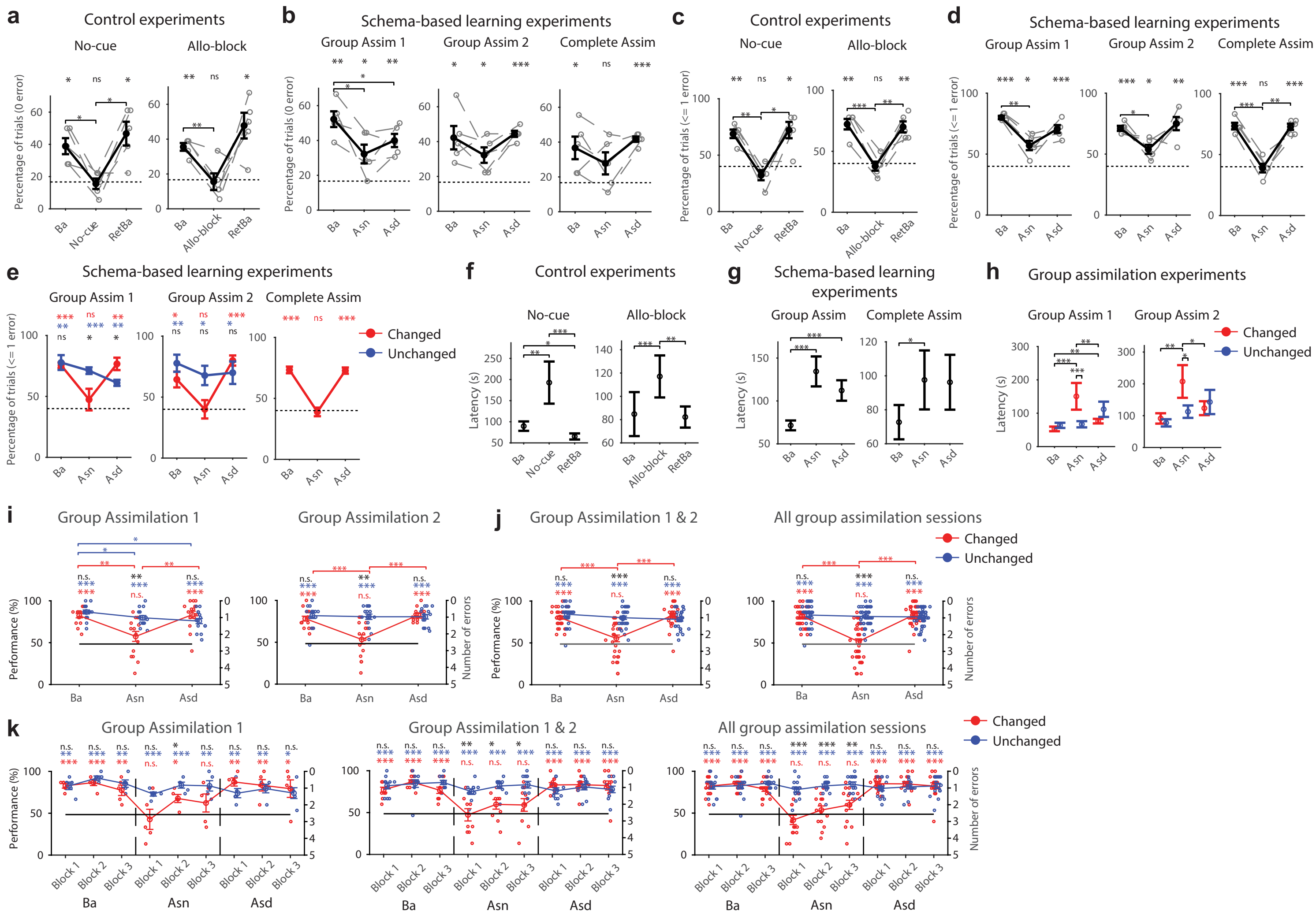

Supplementary Fig. 2: Alternative measures of performance during schema-based rapid learning. a-b Percentage of trials with 0 error for different sessions in control experiments (**a**) and schema-based learning experiments (**b**). **c-d** Percentage of trials with ≤1 error for different sessions in control experiments (**c**) and schema-based learning experiments (**d**). **e** Percentage of trials with ≤1 error for trials with changed and unchanged PAs for different sessions in schema-based learning experiments. **f-g** Latency per trial (seconds, s) from cue sampling to reward port sampling time for different sessions in control experiments (**f**) and schema-based learning experiments (**g**). **h** Latency per trial (s) in trials with changed and unchanged PAs for different sessions in group assimilation experiments. **i-j** Performance across sessions for changed and unchanged PAs during the assimilation learning days compared between changed and unchanged (black), against zero (red/blue) and across learning sessions for both changed (red) and unchanged (blue). These are shown for group assimilation 1 (**i**, left) and 2 (**i**, right) experiment days as well as for combined group assimilation experiment sessions (**j**, left) and combined group assimilation experiment sessions with control stimulation experiment sessions (**j**, right). **k** Performance across blocks for changed and unchanged PAs during the assimilation learning days compared between changed and unchanged (black) and against chance (red/blue). These are shown for group assimilation 1 (**k**, left), combined group assimilation experiment sessions (**k**, middle) and combined group assimilation experiment sessions with control stimulation experiment sessions (**k**, right). The significant effect observed in assimilation session block 2 of group assimilation 1 experiment for the changed PAs (**k**, left) was not observed when we increased the sample size by combining all group assimilation experiments (**k**, middle and right). Two-tailed paired t-tests for comparison between groups and two-tailed one sample t-test against the chance levels of 16.67 in (**a-b**) and 40 in (**c-e**), N=5 in (**a-e**); Two-tailed Rank-sum tests in (**f-h**). Two-tailed paired t-tests in (**i-k**). Source data are provided as a Source Data file. Abbreviations, Ba: Baseline, RetBa: return-to-Baseline, Asn: Assimilation, Asd: Assimilated, Allo: Allocentric.Data are shown as mean±s.e.m. ns, P>0.05, ***P<0.001, **P<0.01, *P<0.05.

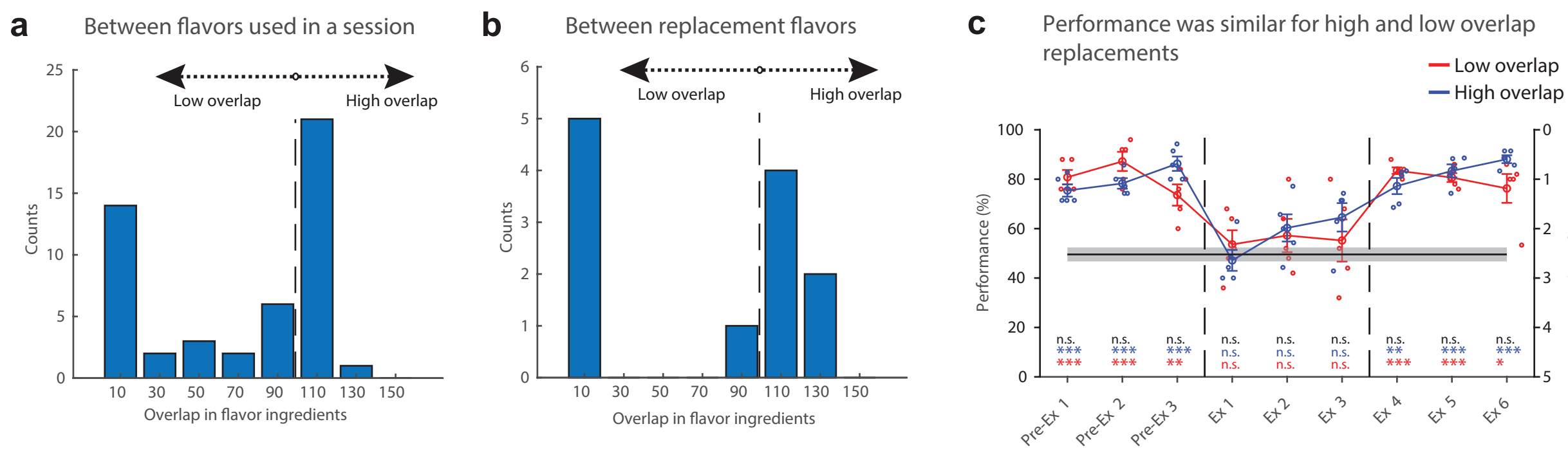

Supplementary Fig. 3: Variability in flavor-cue relatedness between the PAs did not affect the learning performance. a Distribution of the numbers of overlapping flavor ingredients (relatedness) among PAs used within a session. To study the effect of relatedness among flavors on the performance of rats, we divided the flavor-pairs into high and low overlap flavors (median, vertical dashed line). **b** Distribution of the relatedness in between replacement and the original PAs used throughout three experiment days. The replacements were divided into high and low overlap replacement-PAs based on the distribution of relatedness among PA-pairs used within a session (**a**). **c** Learning performance (left ordinate) and errors (right ordinate) did not differ in between high and low overlap replacement-PAs across exposure blocks during experiment sessions. N=9 PA-pairs in each category, Two-tailed paired t-tests in (**c**). Source data are provided as a Source Data file. Abbreviation, Ex: Exposure. Data are shown as mean±s.e.m. in (**c**). ns $P>0.05$, ***$P<0.001$, **$P<0.01$, *$P<0.05$.

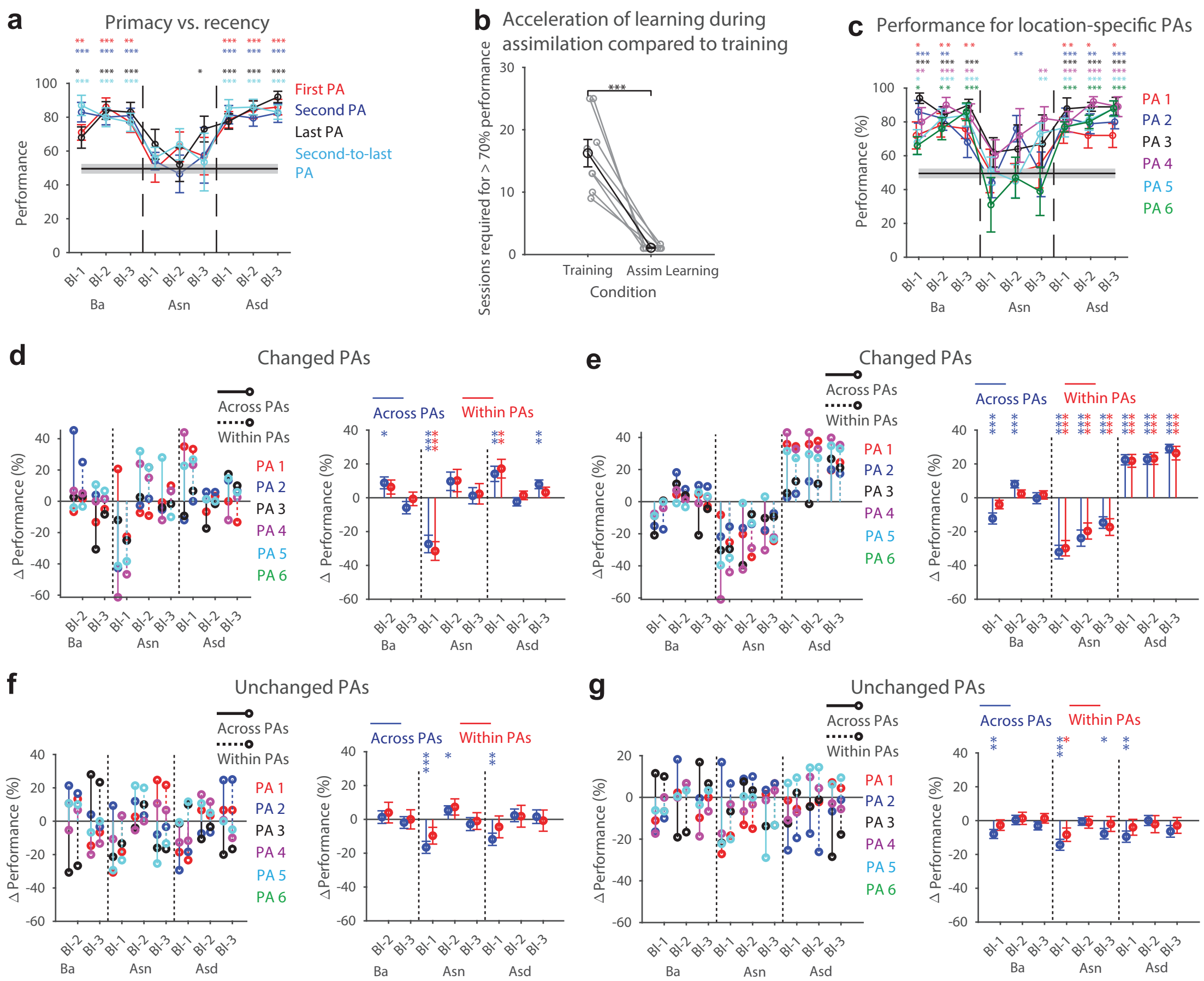

Supplementary Fig. 4: Parallel learning of the changed PAs during assimilation. a Effects of primacy and recency on the performance. Block-wise performance for the PAs introduced first (red) and second (blue) in the first block as well as the PAs introduced last (black) and second-to-last (magenta) on the third block of the assimilation session. Neither primacy nor recency of new PAs biased the performance in the assimilated session. **b** Comparison of sessions required for >70% performance for the new PAs during training vs. assimilation learning. **c** Block-wise performance for location-specific new PAs across learning sessions. Performances evolved together for all new PAs in assimilated session, without an observable bias for any location-specific PA in the maze. **d** Performance change for location-specific new PAs (left) and all PAs (right) in subsequent blocks compared to the performance for the corresponding PAs (dashed stems in the left and red color in the right plots) and other PAs (solid stems in left and blue color in right plots) in the immediate previous blocks. Performances for PAs got worse in the first block of assimilation session and improved in the first block of the assimilated session compared to immediate previous block both when compared to the corresponding PAs and the other PAs. This indicates PAs were not learned in any preferential order, but together. **e** Similar comparisons as in (**d**), but the change was calculated against the previous session (baseline blocks were compared against the average performance in the baseline session). Performances within and across PAs became worse in assimilation session and improved in the assimilated session. This also indicates PAs were not learned in any preferential order, but together. **f-g** Similar plots as in (**d-e**), respectively, but shown for unchanged PAs. N=10 in (**a, c**), N=8 in (**b**), N=9 in (**d-e**) and N=6 in (**f-g**). Two-tailed one-sample t-tests against different chance levels are shown in different colors in (**a**, **c-g**). Two-tailed paired t-test in (**b**). Data are shown as mean±s.e.m. Source data are provided as a Source Data file. Abbreviations, PAs: Paired associations, Ba: Baseline, Asn: Assimilation, Asd: Assimilated, Bl: Block. ***P<0.001, **P<0.01, *P<0.05.

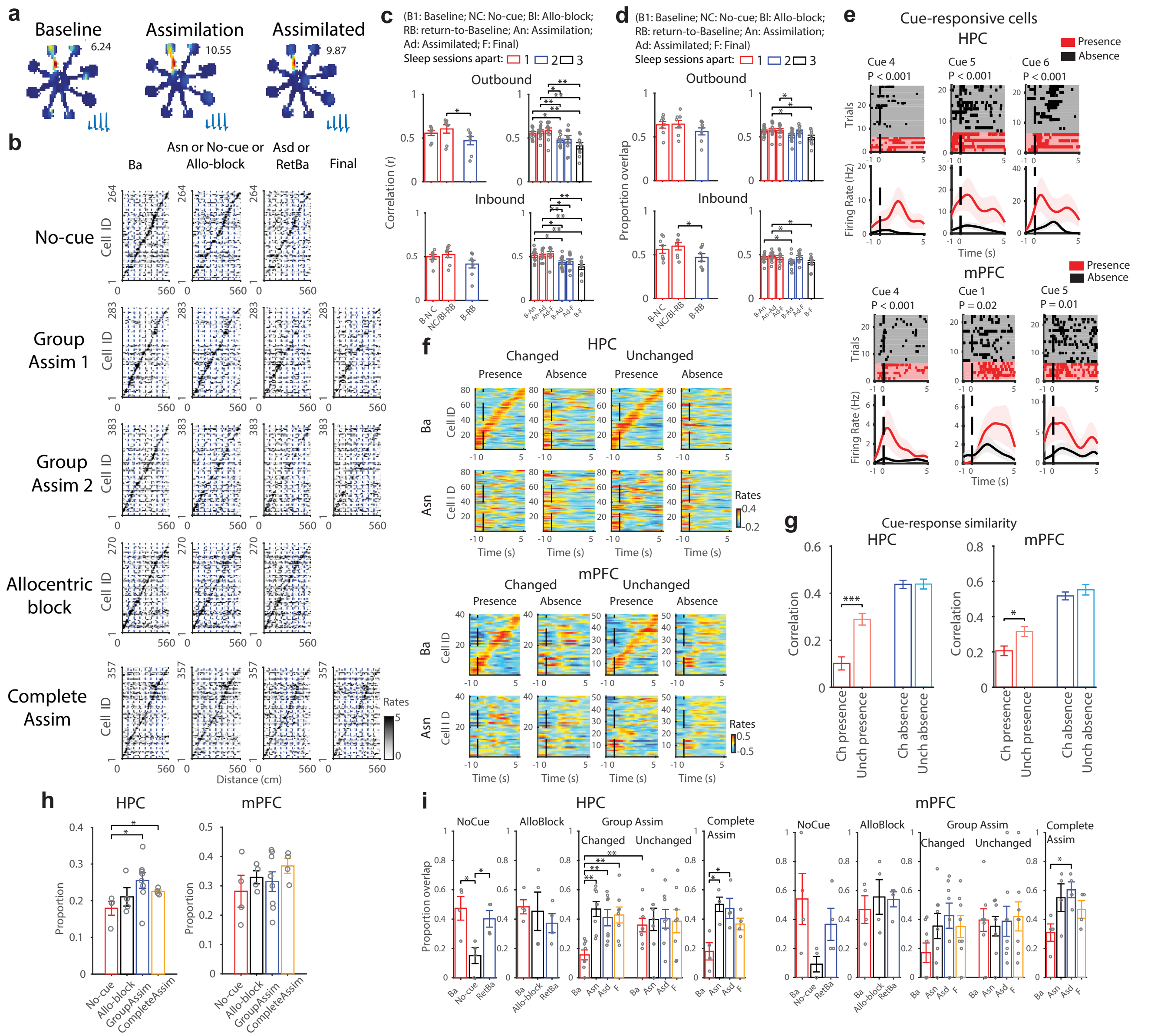

Supplementary Fig. 5: Neither changes in place cells nor in cue responsive cells reflected the substrate of schema during rapid learning. a An example place cell firing rate map in baseline, assimilation and assimilated sessions of group assimilation experiment. Numbers show the peak firing rate, and bottom right shows the amplitude recorded in each electrode of the tetrode, which indicates the stability of the recorded signal across the sessions. **b** HPC linearized spatial firing rates concatenated for all outbound movements (maze center towards the goals) in all arms. HPC pyramidal cells that activated in baseline sessions of each experiment day (first column) are shown and sequentially arranged (on y-axis) according to the peak firing location along spatial trajectory in the baseline sessions. For the other types of sessions (shown in the subsequent columns), spatial firing rates for the same cells are plotted and arranged according to the peak firing location in the baseline sessions. There were no observable distinct effects in the changes in spatial firing patterns of place cells across the recording experiments. **c** HPC single cell spatial correlations grouped by the number of sleep/rest sessions between the sessions of interest. Correlations were compared within and between the groups of session pairs. No differences between the session types were observed within session pairs separated by equal number of sleep/rest sessions, while there was a significant reduction in correlation between session pairs separated by a greater number of sleep/rest sessions. **d** Proportion of HPC cells that maintained their place fields in same arm across session of interest were grouped and compared similarly as in (**c**). No significant difference in proportion overlap was observed within session pairs separated by equal number of sleep/rest sessions irrespective of experiment days, indicative of session types having no distinct effects on the global remapping of place fields. **e** Three examples each of cue responsive cells in HPC and mPFC. Upper panel of each example shows trial by trial spikes aligned to port sampling time divided into trials when a specific cue was present (red) vs. absent (black). On top of upper panel, cue identity and significance are shown. Lower panel shows peri-cue sampling firing rates in presence and absence of the cue. **f** Activation (z-scored) of cue responsive cells (HPC and mPFC) in group assimilation experiment arranged sequentially according to their peak firing time in the presence of the cue in baseline session. The activation is plotted separately for the presence vs. absence of the cue as well as changed vs. unchanged PAs in baseline session (top) and assimilation session (bottom), while keeping sequential arrangement from the baseline session. Note more across-session difference in the firing patterns in the presence of changed cues compared to unchanged ones in both HPC and mPFC. **g** Population vector correlation in firing rates between baseline and assimilation sessions, when cues were changed (Ch) and unchanged (Unch) for cue responsive cells shown in (**f**). **h** Proportion of cells in both HPC and mPFC that were responsive to cues (calculated by combining all sessions; see Methods). **i** Proportion overlap in cue responsive cells, calculated by dividing the number of cells that responded to a cue in the session of interest and continued to respond to the corresponding changed or unchanged cue in the sessions other than the sessions of interest by the number of cells that responded to the cue in the session of interest. Relatively lower proportion of cue responsive cells in baseline session continued to code for changed cues in both HPC (left) and mPFC (right). Two-tailed Rank-sum tests in (**c**, **d**, **g**, **h**, and **i**). Data in (**c-e**, **g-i**) are shown as mean±s.e.m. Detailed statistics are in Supplementary Data 9-10. Source data are provided as a Source Data file. Abbreviations, Ba: Baseline, RetBa: return-to-Baseline, Asn: Assimilation, Asd: Assimilated, F: Final, Ch: Changed, Unch: Unchanged, Allo: Allocentric, . ***P<0.001, **P<0.01, *P <0.05.

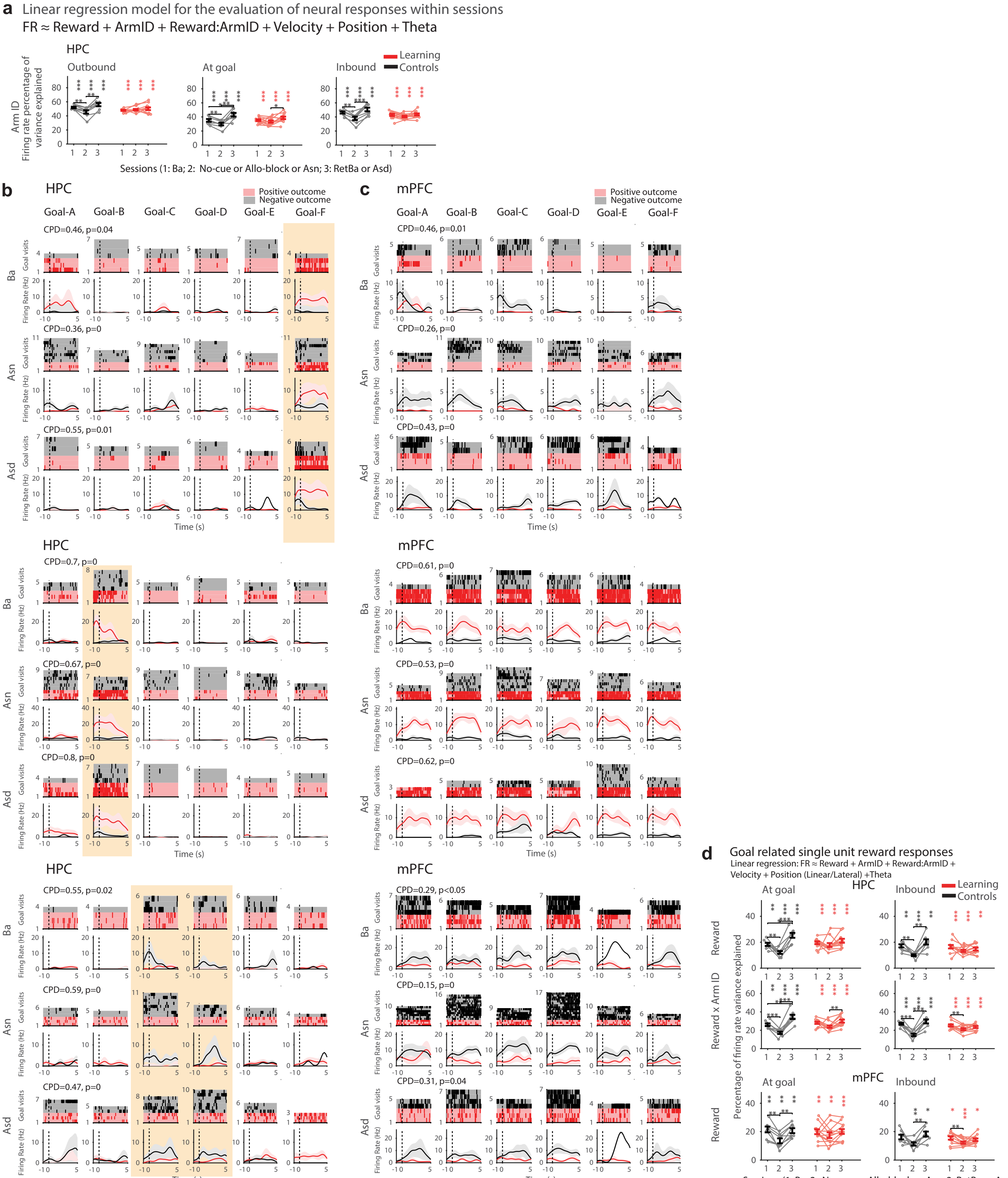

Supplementary Fig. 6: Invariant outcome-responding and outcome at specific goal-responding cells in mPFC and HPC. a Within-session variance in single unit neural firing explained by arm ids during outbound (from center to goal), at goal and inbound (from goal to center). The linear regression model on top was used to quantify the percentage of variance explained by the variables within each sessions of learning and control experiment days. **b-c** Three example HPC cells (**b**) responding invariantly to absence or presence of reward at specific goals (highlighted in orange) and three example mPFC cells (**c**) responding invariantly to reward at all goals across learning sessions (rows). For each example, shown are the visit by visit peri-event rasters at top and the firing rates at bottom, at the time of port entry at 6 different reward ports (goals A-F shown in columns), separately shown for presence and absence of reward. On top of each example, coefficient of partial determination (CPD) shows the percentage of variance explained and P-values (obtained from 1,000 shuffle CPDs generated by randomizing trial-IDs) for outcome responses at specific goals for HPC and outcome responses irrespective of the goals for mPFC.**d** Within-session variance in single unit firing explained by reward in HPC (top) and mPFC (bottom) as well as reward at specific Arms in HPC (middle). Two-tailed paired t-tests were used for both data vs. shuffles and between groups, N=8 and N=12 for control and learning experiments, respectively in (**a**, **d**). Data are shown as mean±s.e.m. Source data are provided as a Source Data file. Abbreviations, Ba: Baseline, RetBa: return-to-Baseline, Asn: Assimilation, Asd: Assimilated, Allo: Allocentric.***P<0.001, **P<0.01, *P <0.05.

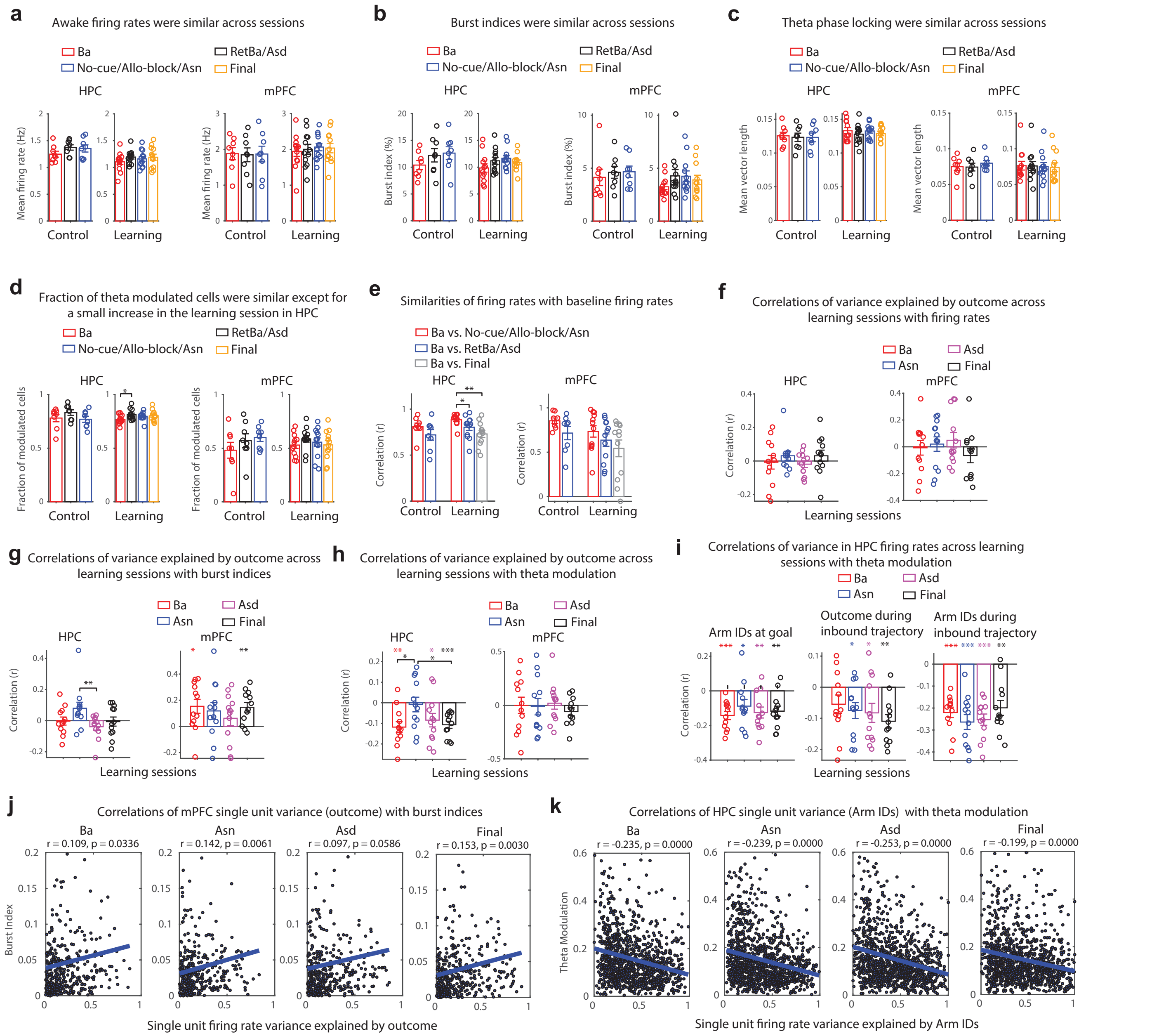

Supplementary Fig. 7: Relationship between basic properties of single units and their firing rate variances during rapid learning. a-c Mean firing rates (**a**), burst indices (**b**) and mean vector lengths (**c**) of HPC (left) and mPFC (right) single units across sessions in the control and learning experiments. Burst indices were calculated as percentage of spikes with ISI <6 ms for HPC and ≤10 ms in mPFC single units. Mean vector length is a measure of theta modulation stength which is a magnitude of the circular mean spike phases occurring in theta cycles. **d** Fraction of theta modulated cells across sessions in the control and learning experiments. The significance was tested for each cell using Rayleigh test for non-uniformity in the spike phase distributions. **e** Correlations of firing rates across sessions with the baseline firing rates. **f-h** Correlations of variance explained by outcome at specific goal in HPC and outcome in mPFC with the mean firing rates (**f**), burst indices (**g**) and theta modulation strength (**h**). There were significant positive correlations of burst indices and significant negative correlations of theta modulation (phase-locking) strengths with the firing rate variances related to outcome in HPC and mPFC, respectively. **i** Correlations of variance explained by arm IDs at goal (left), outcome (middle) and arm IDs (right) during the inbound movement trajectory in HPC firing rates with the theta modulation strengths. There was significant negative relationship between single unit firing rate variances in multiple session types with the theta modulation. **j** Same analyses as (**h**), but shown for all mPFC single units in the respective sessions. **k** Same analyses as in the right panel in (**i**), but shown for all HPC single units in the respective sessions. N=8 and 12 for control and learning sessions, respectively in (**a-i**). Data are shown as mean±s.e.m in (**a-i**).Two-tailed Signed-rank tests for significance within group and two-tailed rank-sum tests for significance across groups. Person's correlations were used to measure similarities. Source data are provided as a Source Data file. Abbreviations, Ba: Baseline, RetBa: return-to-Baseline, Asn: Assimilation, Asd: Assimilated, Allo: Allocentric.***P<0.001, **P<0.01, *P<0.05.

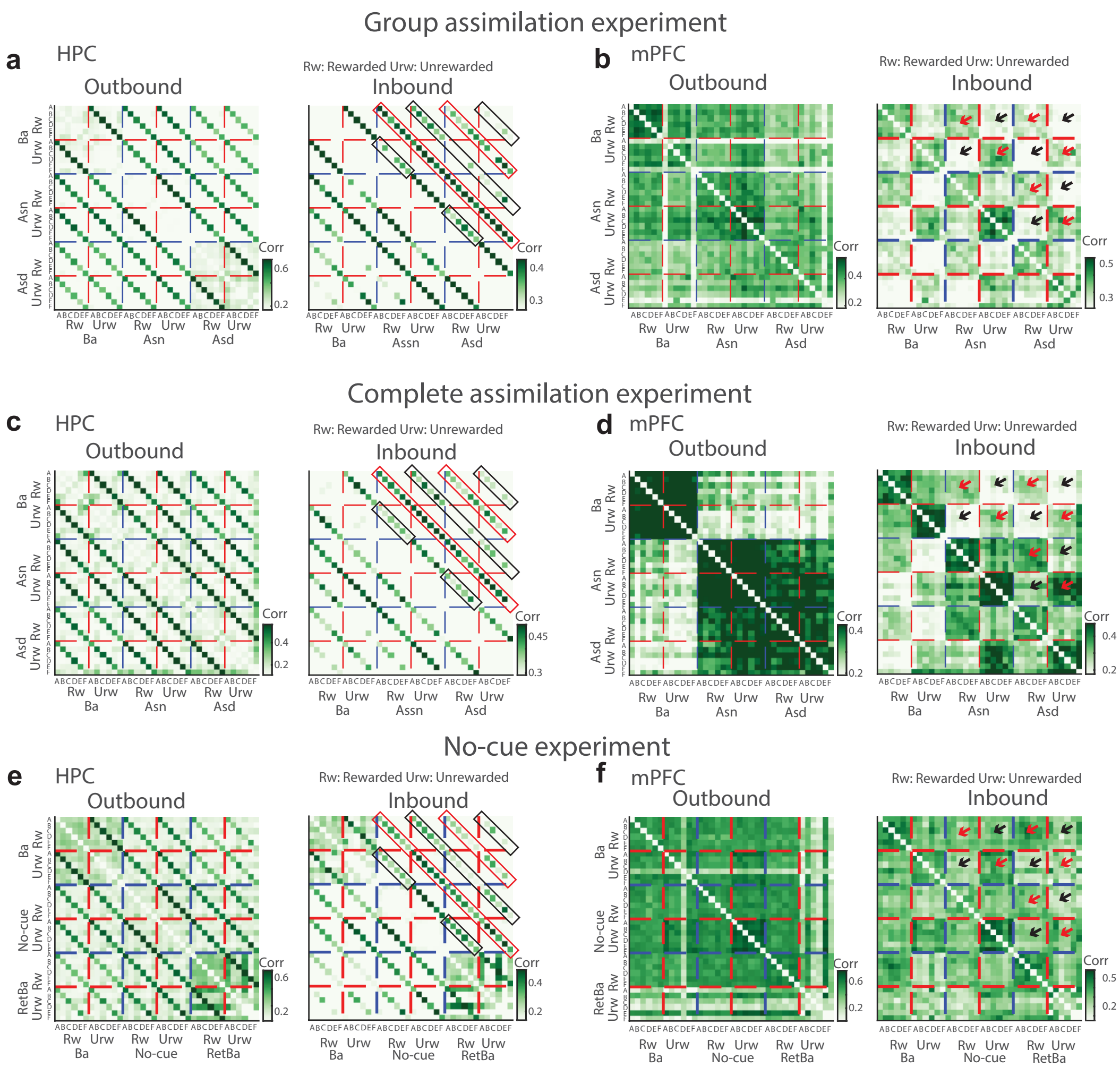

Supplementary Fig. 8: Spatial RSM plots. a-b Representational similarity matrices (RSM) for neuronal ensemble activation at the 6 goal-arms with two outcome types (demarcated by red dashed lines), each across three session types (demarcated by blue dashed lines), when rats were outbound (left), and inbound (right) for HPC (**a**) and mPFC (**b**) in the group assimilation experiment. **c-d** RSMs are shown in the same format as (**a-b**) but for the complete assimilation experiment. Higher population vector correlations for same (red rectangles) compared to opposite outcome type (black rectangles) at specific goals in HPC (**a**, **c**, right), and same (red arrows) compared to opposite outcome type (black arrows) irrespective of goal identities in mPFC (**b**, **d**, right), depicted the similar invariant outcome responses across sessions in the spatial firing after goal sampling in both group and complete rapid learning days. **e-f** RSMs are shown in the same format as (**a-b**), but for No-cue experiment. Note the level of invariant outcome responses on No-cue day (**e-f**) are not as prominent as on rapid learning days. N=758 (HPC) and 264 (mPFC) in (**a-b**), N=412 (HPC) and 122 (mPFC) in (**c-d**), N= 309 (HPC) and 83 (mPFC) in (**e-f**). Source data are provided as a Source Data file. Abbreviations, Ba: Baseline, RetBa: return-to-Baseline, Asn: Assimilation, Asd: Assimilated.

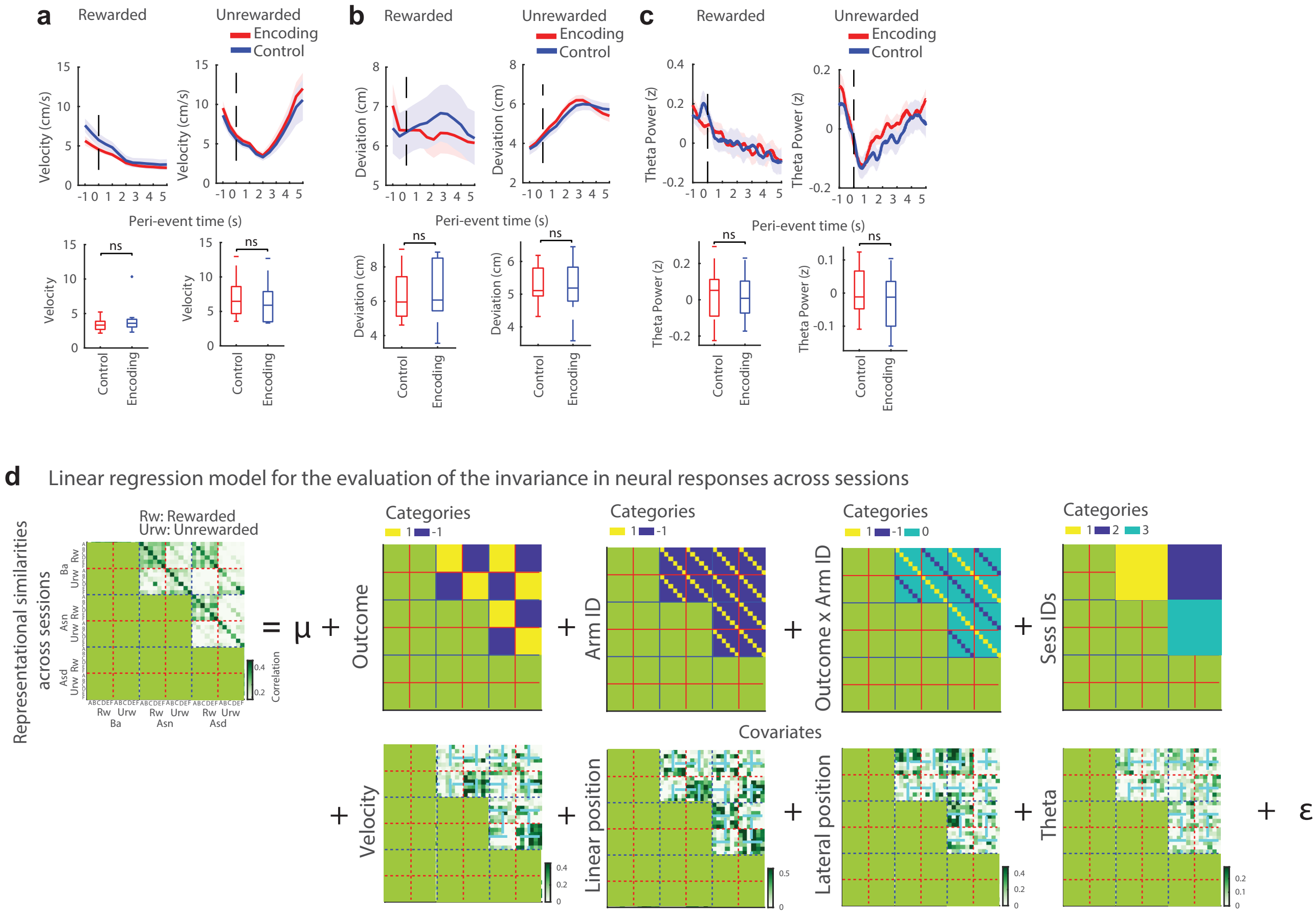

Supplementary Fig. 9: Consideration of behavioral variables in rewarded and unrewarded arm visits. a-c Comparison of velocity (**a**), deviation from the center of the track (**b**) and theta power (**c**) in between encoding (assimilation) and control (no-cue) sessions during goal port sampling periods in rewarded (left) and unrewarded (right) arm visits. There were no significant differences of variables in response to presence or absence of reward between learning and control sessions. Data in top panels are shown as mean±s.e.m. Data in bottom panels are shown as boxes of 25th-75th percentiles with median as center and whiskers covering twice the interquartile range. Two tailed Rank-sum tests are used in (**a-c**). **d** Linear regression model used to quantify the effects of different factors on the invariance of neural representations across sessions. Note the absence of systematic similarities in covariates across trial types across session. μ represents the grand mean and ε represents the error. Source data are provided as a Source Data file. Abbreviations, Ba: Baseline, Asn: Assimilation, Asd: Assimilated. ns, P>0.05.

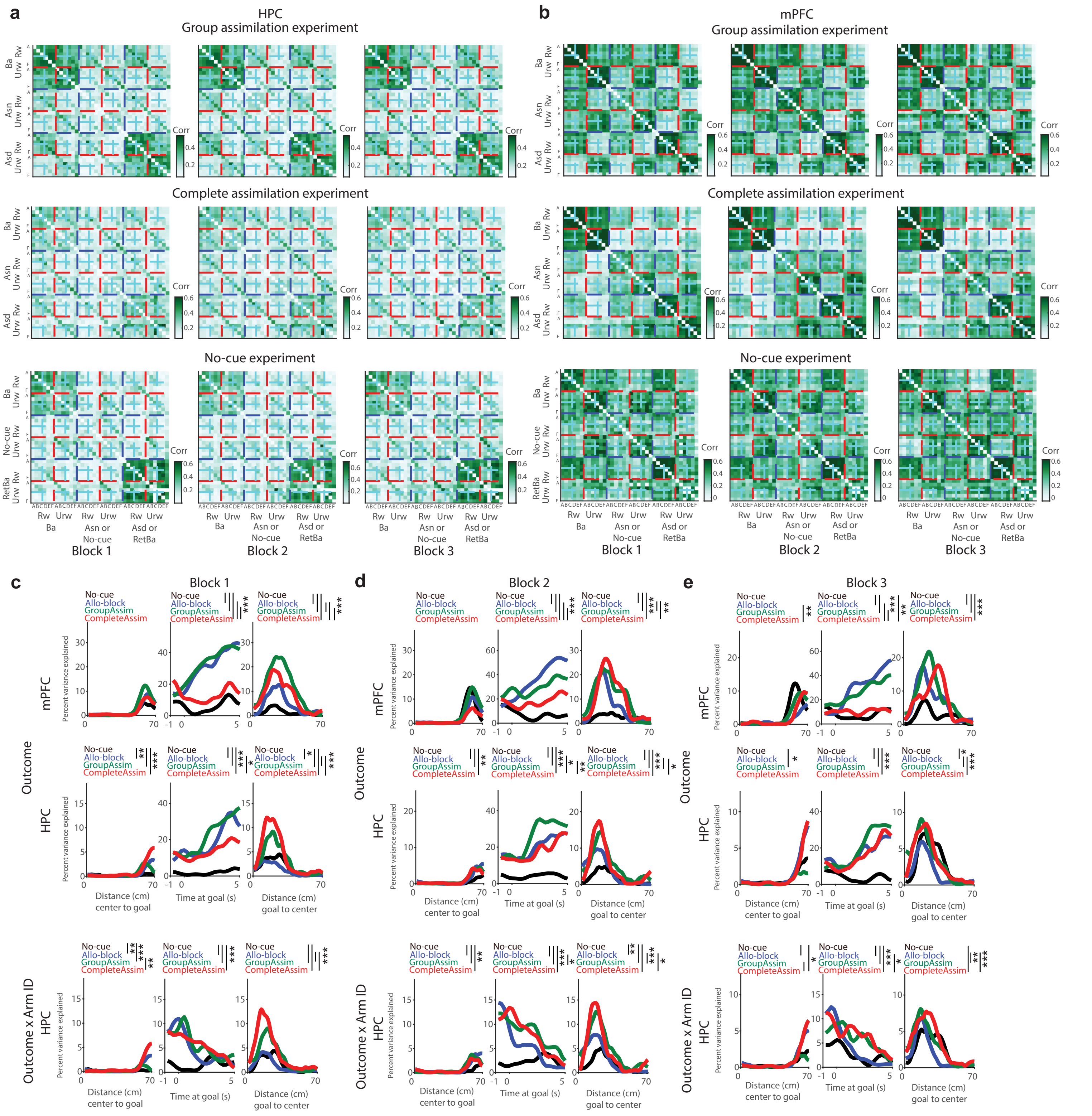

Supplementary Fig. 10: Invariant responses to specific goal outcome in HPC and general outcome in mPFC emerged on the first exposure in assimilation session. a-b RSMs for neuronal ensemble activation at the 6 goals with two outcome types, each across three session types, but with only single block (blocks 1, 2 and 3 shown in left, middle, and right, respectively) of assimilation, no-cue or allocentric-block session data included in the analysis. Higher population vector correlations were observed for the same outcome type at each goal in the HPC neural ensembles (**a**) and outcome type irrespective of the goal identities in the mPFC neural ensembles (**b**) while rats were at goal in all three blocks (columnar panels) in group (top row) and complete (middle row) assimilation experiments, but not in no-cue experiment (bottom row). **c-e** Percentage of neuronal activity variance explained by similarity in outcome types (**c-e**, top and middle row) for HPC and mPFC neural ensembles and outcome types at specific arms (**c-e**, bottom row) for HPC neural ensemble across sessions were compared among no-cue, allo-block, group and complete assimilation experiments when rats were outbound (left), at goal (middle), and inbound (right). The analysis was conducted across sessions (only across session portions of RSMs involving assimilation/no-cue/allo-block sessions were included in the calculation) by including one block at a time (blocks 1, 2 and 3 shown in **c**, **d** and **e**, respectively). At goal, hippocampal invariance to outcome at specific arms (**c**, bottom) was significantly reduced on no-cue compared to allo-block and group assimilation experiments. While rats were inbound, significantly weaker invariance to outcome and the outcome at specific arms were observed in HPC in both no-cue and allo-block experiments. However, deficits in mPFC invariance to outcome were evident only on no-cue compared to all the other experiment conditions at goal and while rats were inbound. These distinct invariances in the HPC and mPFC neural ensembles during first exposure to new PAs i.e., in block 1 (**c**) remained mostly similar in block 2 (**d**), but were diminished on block 3 (**e**) of the assimilation/no-cue/allo-block sessions. Two-tailed Student's t-tests for testing between groups in (**c-e**); N=758 and 264 in top, N=412 and 122 in middle, N=309 and 83 in bottom, (**a-b**), respectively. Source data are provided as a Source Data file. Abbreviations, Ba: Baseline, RetBa: return-to-Baseline, Asn: Assimilation, Asd: Assimilated, Allo: Allocentric.***P<0.001, **P<0.01, *P<0.05.

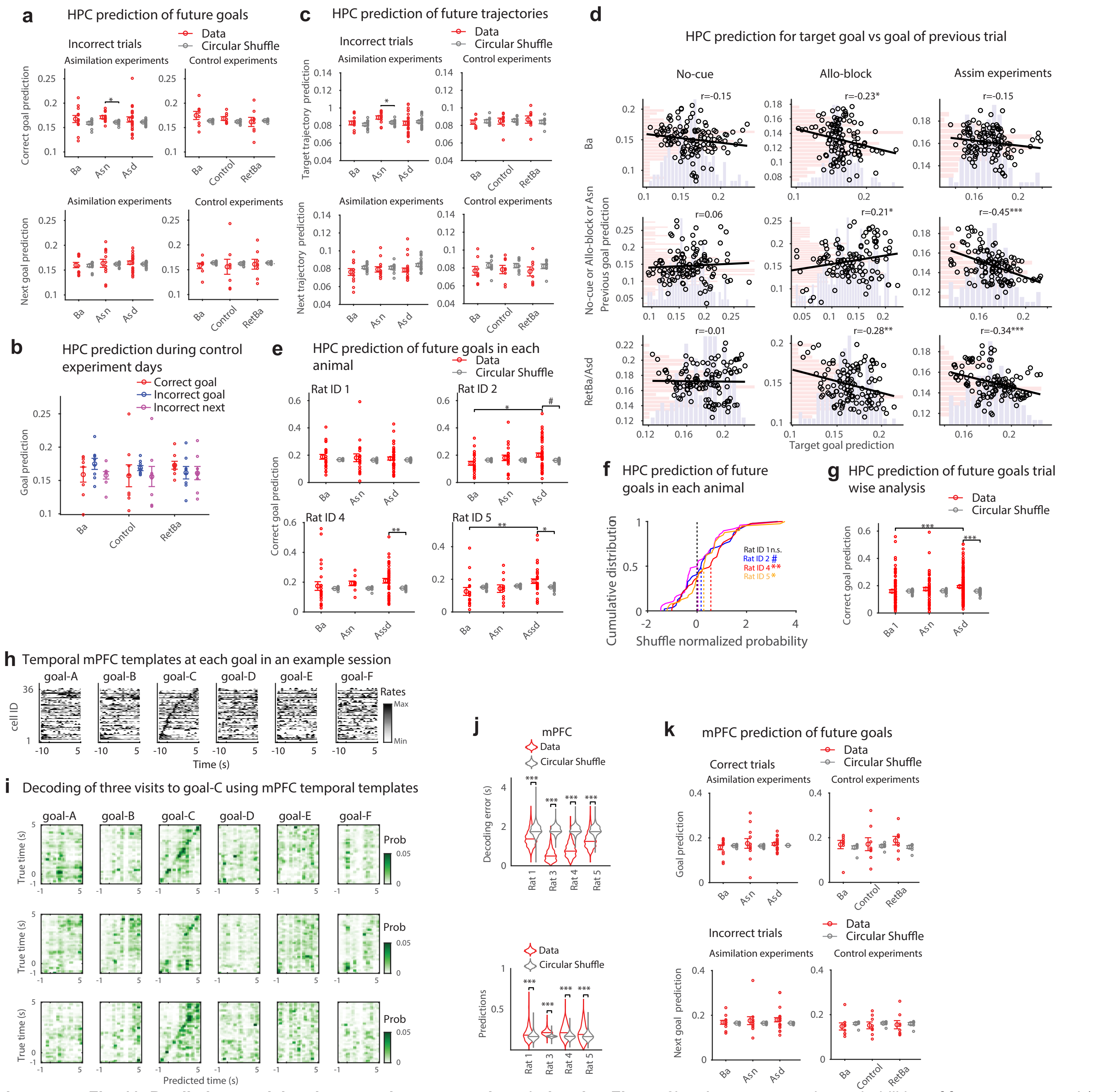

Supplementary Fig. 11: Predictive spatial and temporal representations (related to Figure 3). **a** Average posterior probabilities of future correct goal (top) and incorrect next goal (bottom) prediction for incorrect trials for each session type of assimilation learning and control days, and their corresponding shuffles (Two-tailed Signed-rank test; N=24 for Assimilated sessions). **b** Future goal prediction in correct trials compared against target correct goal and incorrect next goal predictions in incorrect trials during control experiment days (Two-tailed Signed-rank tests; N=8). **c** Average posterior probabilities of target correct arm (top) and upcoming arm (bottom) trajectory prediction for incorrect trials for each session type of assimilation learning and control days, and their corresponding shuffles. Decoding of HPC place cell ensemble activity during peri-cue sampling epoch (50 ms time bins) using concatenated spatial trajectories of the 6 arms (1 cm bins, by direction outbound/inbound) and predictions for upcoming arm were compared against the shuffles (Signed-rank test; N=24 for assimilated session). **d** For each trial in different session types, goal predictions (vertical blue histogram) are plotted against predictions for previous goal in the previous trial (horizontal red histogram). Note the positive correlation in allo-block test during allo-block experiment day, indicating both previous and future goals were predicted similarly at cue presentation. Also, note the negative correlation in assimilated session with a shift of future goal prediction (in x axis) histogram toward greater values, indicating that greater predictions of future goal in assimilated session co-occurred with lesser interference from memory of previously visited goal. **e** Average posterior probabilities of future correct goal in each trial shown for different subjects, and their corresponding shuffles (Two-tailed Signed-rank test for comparison against zero and two-tailed ranksum tests between groups). **f** Normalized posterior probabilities of future correct goal (Data-shuffle/shuffle) in each trial shown for different subjects (Two-tailed Signed-rank test). **g** Average posterior probabilities of future correct goal in each trial shown for all subjects, and their corresponding shuffles (Two-tailed Signed-rank test for comparison against zero and two-tailed ranksum tests between groups). **h** Example mPFC temporal sequence template for goal-C compared to all other goals based on the order of time of peak average firing of neurons at goal-C. **i** Decoding of instantaneous mPFC activity during 3 correct/rewarded goal visits to goal C (rows) using concatenated templates of all A-F goals as prior. Heatmaps show probabilities of predicted time for all 6 goals (columns) against true time spent at goal C. **j** Average instantaneous decoding errors (top) and predictions of goals (bottom) compared against shuffles (Two-tailed Rank-sum tests). **k** Average posterior probabilities of future goal prediction for correct (top) and error (bottom) trials for each session type of assimilation learning and control days, and their corresponding shuffles (Two-tailed Signed-rank test; N=24 for assimilated sessions). Neither significant predictions for correct goal (top) nor next goal (bottom) were found in any session on rapid learning days. Two-tailed Rank-sum tests for comparison between groups and for comparison against shuffles in (**j**). Data in (**a-c**, **e**, **g** and **k**) are shown as mean±s.e.m. Detailed statistics in Supplementary Data 3. Source data are provided as a Source Data file. Abbreviations, Ba: Baseline, RetBa: return-to-Baseline, Asn: Assimilation, Asd: Assimilated. ***P<0.001, **P<0.01, *P<0.05, #P<0.10.

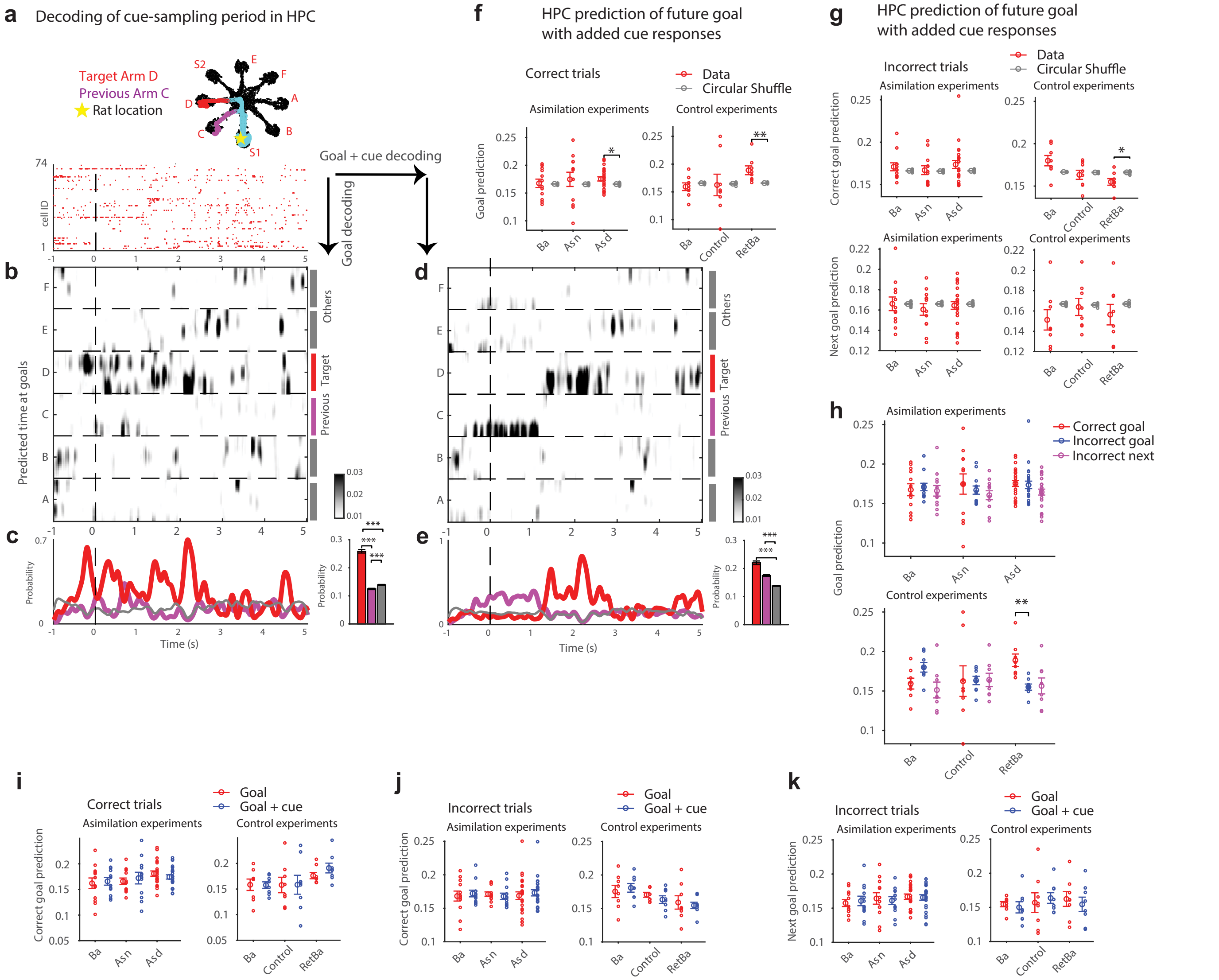

Supplementrary Fig. 12: HPC goal predictive ensembles at start arms did not additionally predict corresponding cues. a Example correct trial during Assimilated session (current rat location at start arm 1, top) and spiking raster plot (bottom) for HPC pyramidal neurons during peri-cue sampling epoch. **b** Decoding of HPC ensemble activity during peri-cue sampling epoch (x axis, 50 ms bins) using the concatenated 6 templates at goal (250 ms, time bins). Resulting posterior probabilities for 6 goals were stacked on y-axis. **c** Sum (left) and averaged sum (right) of decoded probabilities for prediction of previous trial goal (magenta), future target goal (red) and other goals (gray). **d** Same decoding analyses as in (**b**), but using the concatenated 6 templates each with combined goal and cue responses for the corresponding cues (250 ms, time bins). The cue responses from the same block were excluded from the templates. **e** Sum (left) and averaged sum (right) of decoded probabilities for prediction of previous trial goal (magenta), future target goal (red) and other goals (gray) combined with their corresponding cues. **f** Average posterior probabilities of future combined goal + cue predictions for correct trials for each session type of assimilation learning and control days, and their corresponding shuffles (Two-tailed Signed-rank test; N=24 for Assimilated sessions). **g** Same analyses as in (**f**), but for incorrect trials correct goal + cue (top) and incorrect next goal + cue (bottom). **h** Future goal + cue prediction in correct trials compared against correct goal and incorrect next goal predictions combined with their corresponding cues in incorrect trials during assimilation days (Two-tailed Signed-rank test; N=24 for Assimilated sessions). **i-k** Comparison of correct goal prediction in correct trials (**i**), correct goal prediction in incorrect trials (**j**) and incorrect next goal prediction in incorrect trials (**k**) with the corresponding prediction of combined goal and cue, for each session type of assimilation learning and control days (Two-tailed Signed-rank test; N=24 for Assimilated session). Note the absence of additional improvements in prediction of goal with the addition of cue responses in the templates. Data in (**c**, **e**-**k**) are shown as mean±s.e.m. Source data are provided as a Source Data file. Abbreviations, Ba: Baseline, RetBa: return-to-Baseline, Asn: Assimilation, Asd: Assimilated. ***P<0.001, **P<0.01, *P<0.05.

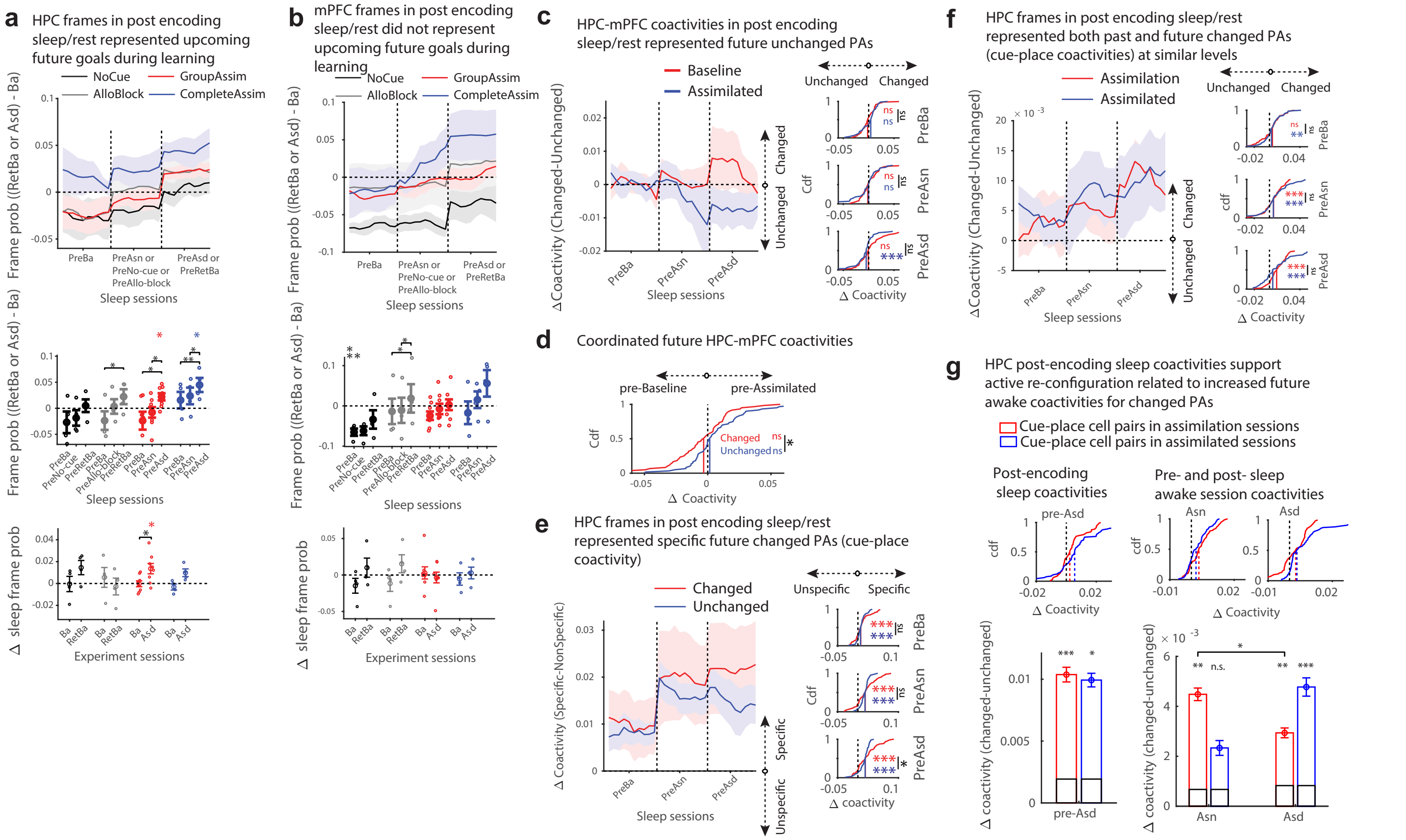

Supplementary Fig. 13: Representations of neural correlates of behavior during sleep/rest (related to Figure 4). a-b Increase in representation of upcoming assimilated-session goals in HPC (**a**) and mPFC (**b**) during pre-assimilated sleep/rest in group and complete assimilation learning days compared to other sleep sessions and control days. Bias toward assimilated or return-to-baseline compared to baseline session goal was computed on their average decoding probability during HPC or mPFC sleep/rest frames across all sleep sessions (top, sleep divided into 10 equal time-bins) and compared (middle) across all experimental days. Changes in frame probabilities from pre-assimilation to pre-assimilated sleep/rest were compared between assimilated and baseline sessions goals (bottom). Corresponding sessions were compared in the corresponding sleep during control days (bottom). Two-tailed one sample t-tests for data vs. 0 and two-tailed paired t-tests for comparison between groups, N=4 for no-cue, allo-block and complete assimilation experiments and N=8 for group assimilation experiment. In HPC frames the increase in the pre-assimilated sleep/rest was higher for assimilated session goals compared to the baseline session goals in group and complete assimilation experiments (**a**, middle), which was similar to the bias observed for the upcoming assimilated session trajectories (see Fig. 4b). **c** Bias toward changed goals in cofiring of mPFC cue and HPC place responsive cells during different sleep sessions are shown (left) and compared (cdfs, right) for the baseline and assimilated session goals in group assimilation days. Stronger bias was observed towards cofiring of unchanged PAs (HPC place and mPFC cue responsive cells, right) for assimlated session in pre-assimilated sleep/rest. **d** Changes in cofiring of HPC place and mPFC cue responsive cells from pre-baseline to pre-assimilated sleep/rest. The increase for the upcoming unchanged PAs was larger than for the changed PAs in the cofiring across brain regions. **e** Bias toward changed PAs in cofiring of HPC cue-place cell pairs increased for cell pairs specific to the PAs compared to the non-specific cell pairs from different PAs. **f** Bias toward cofiring of cue-responsive and place cells in HPC frames during different sleep/rest sessions are shown (left) and compared (cdfs, right) for assimilation and assimilated session PAs in group assimilation days. Note that the cofirings for the encoding session PAs were not greater than the upcoming session PAs in the post-encoding sleep. **g** HPC cue-place cell pair coactivites in the post-encoding sleep (left) and awake HPC frames (right) for changed PAs detected in assimilation and assimilated sessions. Note similar levels of both past and future changed PAs in post-encoding sleep frames (left) followed by emergence of the future session awake coactivities during the post-encoding awake assimilated sessions (chance levels shown in black bars). Top panels show data distribution using cdf plots and corresponding bar graph in the bottom panels show mean±s.e.m. Two-tailed Signed-rank tests for data vs. 0 and between groups in (**c-f**); Two-tailed Signed-rank tests for comparison against baseline session cue-place pair coactivities and between groups (**g**); N=60 for (**c-d**) and 80 for (**e-g**). Data in (**a-c** and **e-g**) are shown as mean±s.e.m. Detailed statistics are in Supplementary Data 4. Source data are provided as a Source Data file. Abbreviations, PAs: Paired associations, Ba: Baseline, RetBa: return-to-Baseline, Asn: Assimilation, Asd: Assimilated, Allo: Allocentric. ns, P>0.05, ***P<0.001, **P<0.01, *P<0.05.

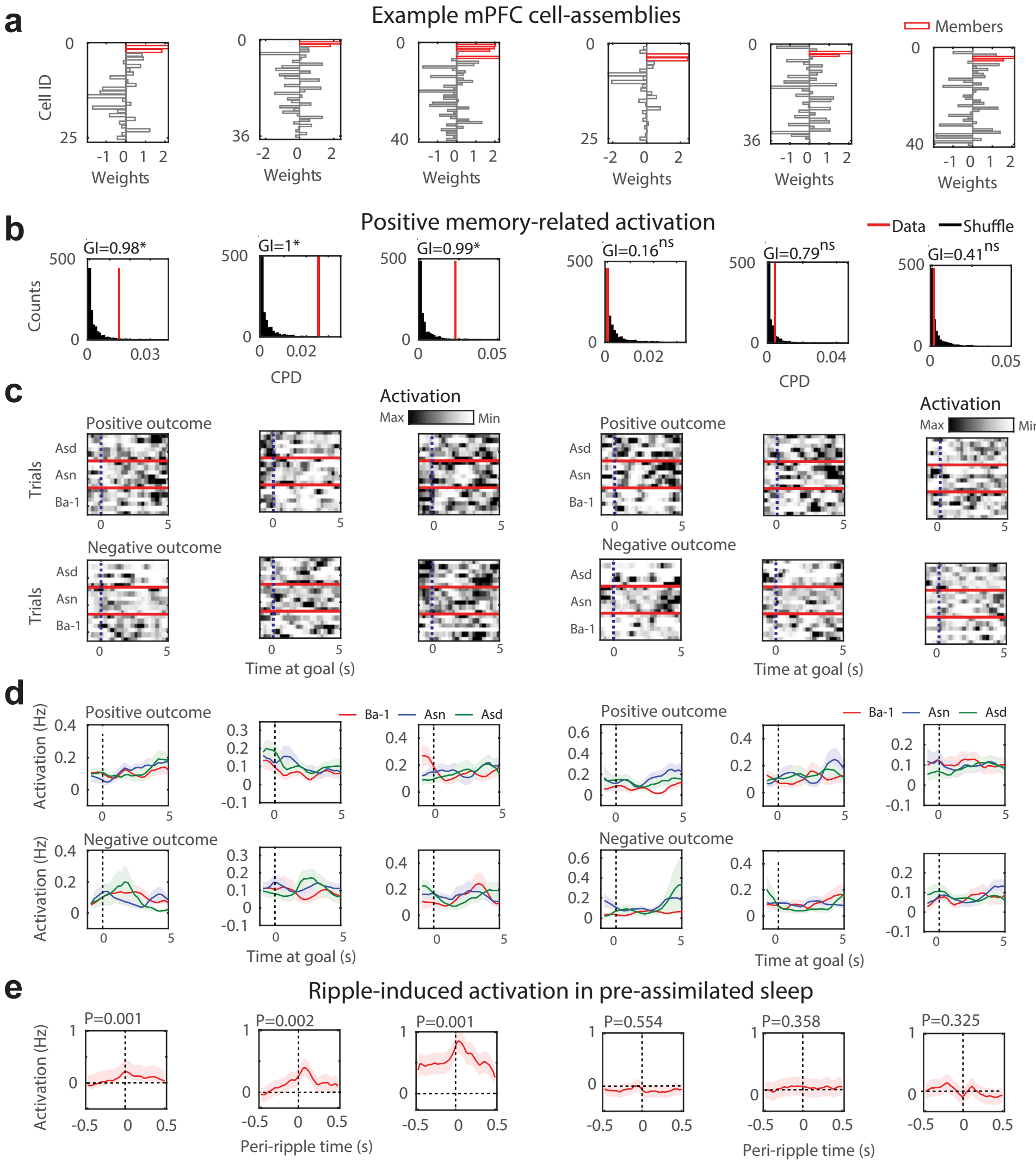

Supplementary Fig. 14: Peri-ripple activation of memory-related mPFC cell-assemblies (related to Figure 5). a Six example cell-assemblies with participating neurons (red, >1.5 z-score) detected in the assimilated session. **b** CPD (coefficient of partial determination, red, see Methods) for each cell-assembly compared against shuffle CPDs in black (GI, positive outcome-memory generalization index >0.95 are considered significant). **c-d** Heatmaps showing activation of corresponding mPFC assemblies (**a**) in different sessions (**c**, goals A-F in rows) and averaged activation (**d**) during positive (top) and negative outcome (bottom). **e** Peri-ripple mPFC assemblies (**a**) activation during pre-assimilated sleep/rest (P-values on top; Two-tailed Rank-sum tests for post- vs. pre-ripple onset, 250ms). Activations were normalized against activations during 1,000 sleep shuffles with randomized ripple times. Note a higher peri-ripple activation for the first 3 cell-assemblies which also had significant positive outcome generalization across sessions during assimilation learning. Data in (**d-e**) are shown as mean±s.e.m. Source data are provided as a Source Data file. Abbreviations, Ba: Baseline, Asn: Assimilation, Asd: Assimilated. ns P>0.05, *P <0.05.

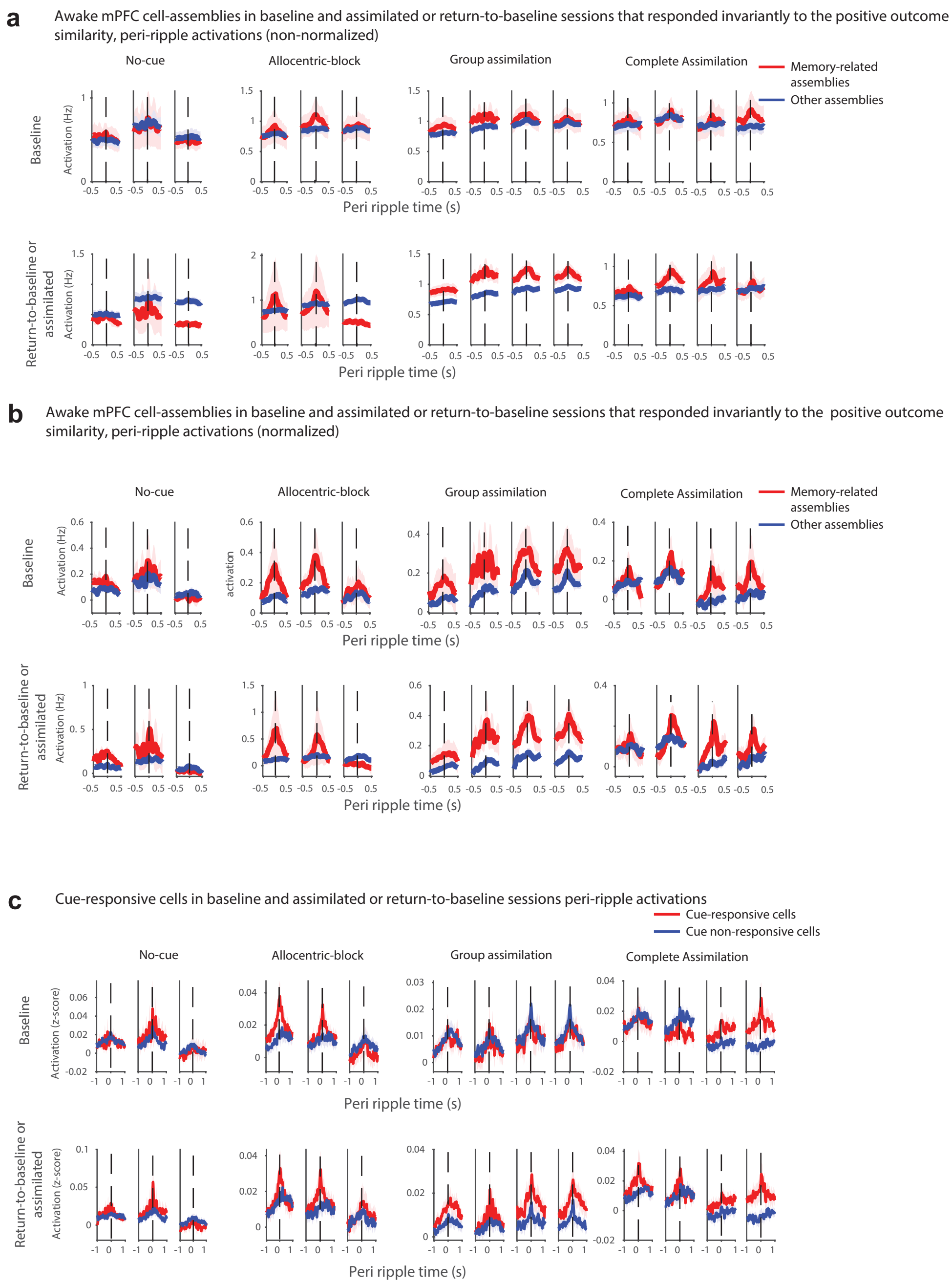

Supplementary Fig. 15: HPC-mPFC coordination in sleep/rest during learning and memory consolidation (related to Figure 5). a Peri-ripple activation of baseline (top row) and return-to-baseline or assimilated (bottom row) session mPFC cell assemblies during no-cue, allocentric-block, group and complete assimilation days shown in pre-baseline, pre-no-cue or pre-allo-block or pre-assimilation, pre-assimilated or pre-return-to-baseline, and final sleep/rest sessions for cell assemblies that generalize positive outcome across sessions (memory-related, red) vs. those that don't (blue). **b** Similar plot as in (**a**), but shuffle normalized activations are plotted. **c** Similar plot as in (**a**), but normalized activations for respective mPFC cue-responsive cells (red) and cue non-responsive cells (blue) are plotted. Source data are provided as a Source Data file.

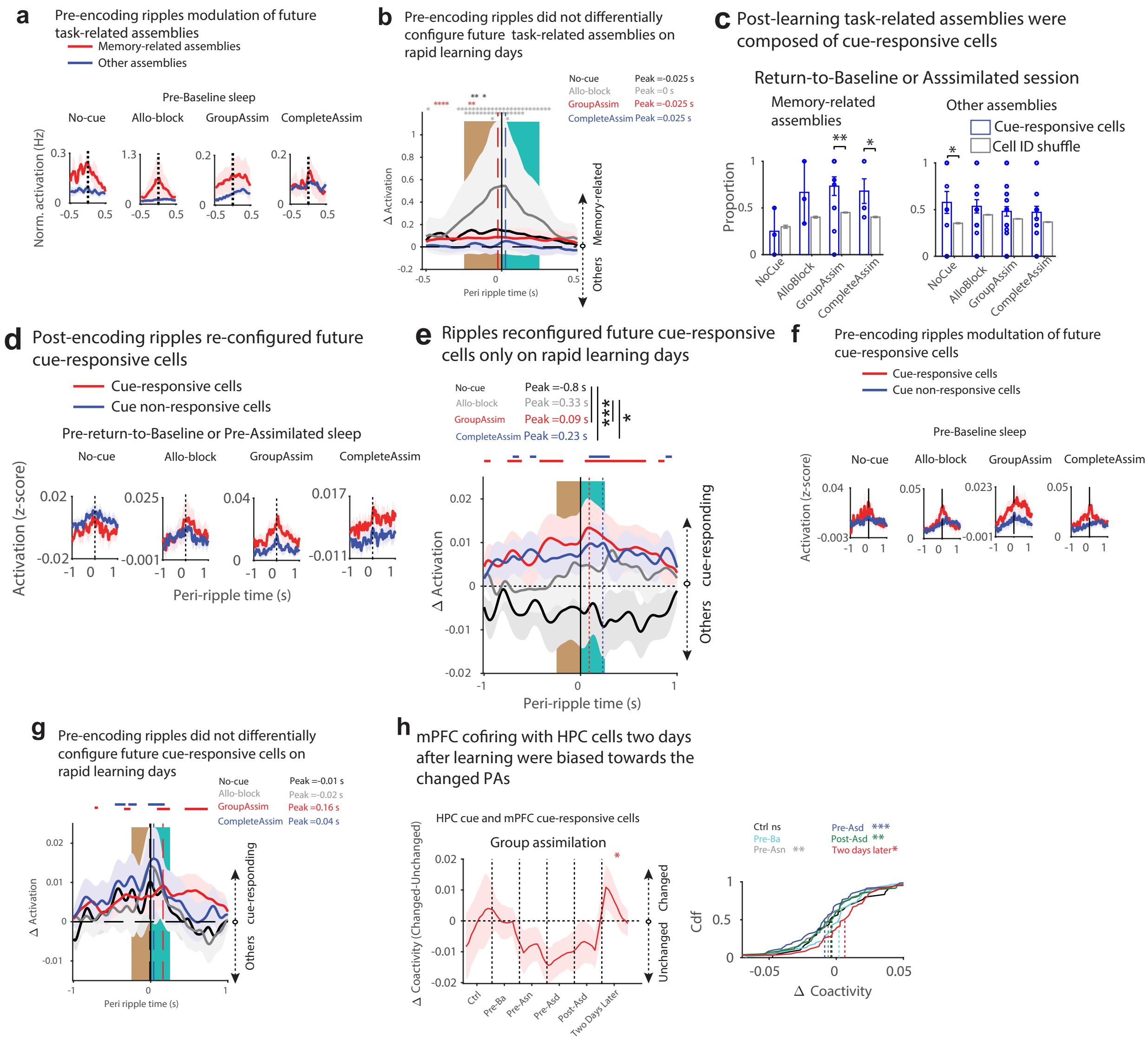

Supplementary Fig. 16: mPFC network predictive reorganization. a Peri-ripple activation of memory-related vs. other, assimilated or return-to-baseline cell assemblies shown in pre-baseline sleep/rest. **b** Ripple-induced difference in peri-ripple activation (memory-related minus other assemblies) of assimilated session assemblies from (**a**). Peak activation times indicated in legend and by vertical dashed lines. Note the absence of significant difference in ripple modulation across experiment days. **c** Proportions of cell members of memory-related (left) and other cell-assemblies (right) from assimilated or return-to-baseline sessions vs. corresponding shuffles participating as cue-responsive cells. **d-e** Same format as (**a-b**), respectively, but for the activation of cue-responsive cells detected in assimilated sessions in pre-return-to-baseline or pre-assimilated sleep/rest sessions. Lines on top represent periods of significant activation (**e**). Note the significantly greater activation in all assimilation learning days compared to the control experiment days. **f-g** Same format as (a-b), respectively, but for the activation of cue responsive cells detected in assimilated sessions in pre-baseline sleep/rest sessions. Lines on top represent periods of significant activation (**g**). Note the absence of significant difference in ripple modulation across experiment days. **h** Bias in the coactivity of mPFC cue and HPC cue responsive cell pairs towards the unchanged associations in the pre-assimilated sleep/rest was significantly reversed (bottom, cdf) in the pre-baseline sleep/rest two days later. Ctrl (top plots, x axis label) shows the non-significant bias for baseline session in group assimilation 1 experiment in the pre-baseline sleep/rest. (**b**, **e** and **g**), Post- minus pre-ripple onset, 250 ms shown in colored patches compared among experiment days; (**h**), Two-tailed Signed-rank tests, N=60 for all sleep/rest sessions except N=30 for 'Ctrl'; (**c**), Two-tailed Student's t-tests for data against shuffles. Errors indicate s.e.m. Detailed statistics in Supplementary Data 5-7. Source data are provided as a Source Data file. Abbreviations, PAs: Paired associations, Ba: Baseline, Asn: Assimilation, Asd: Assimilated, Allo: Allocentric. ns, P>0.05, ***P<0.001, **P<0.01, *P<0.05.

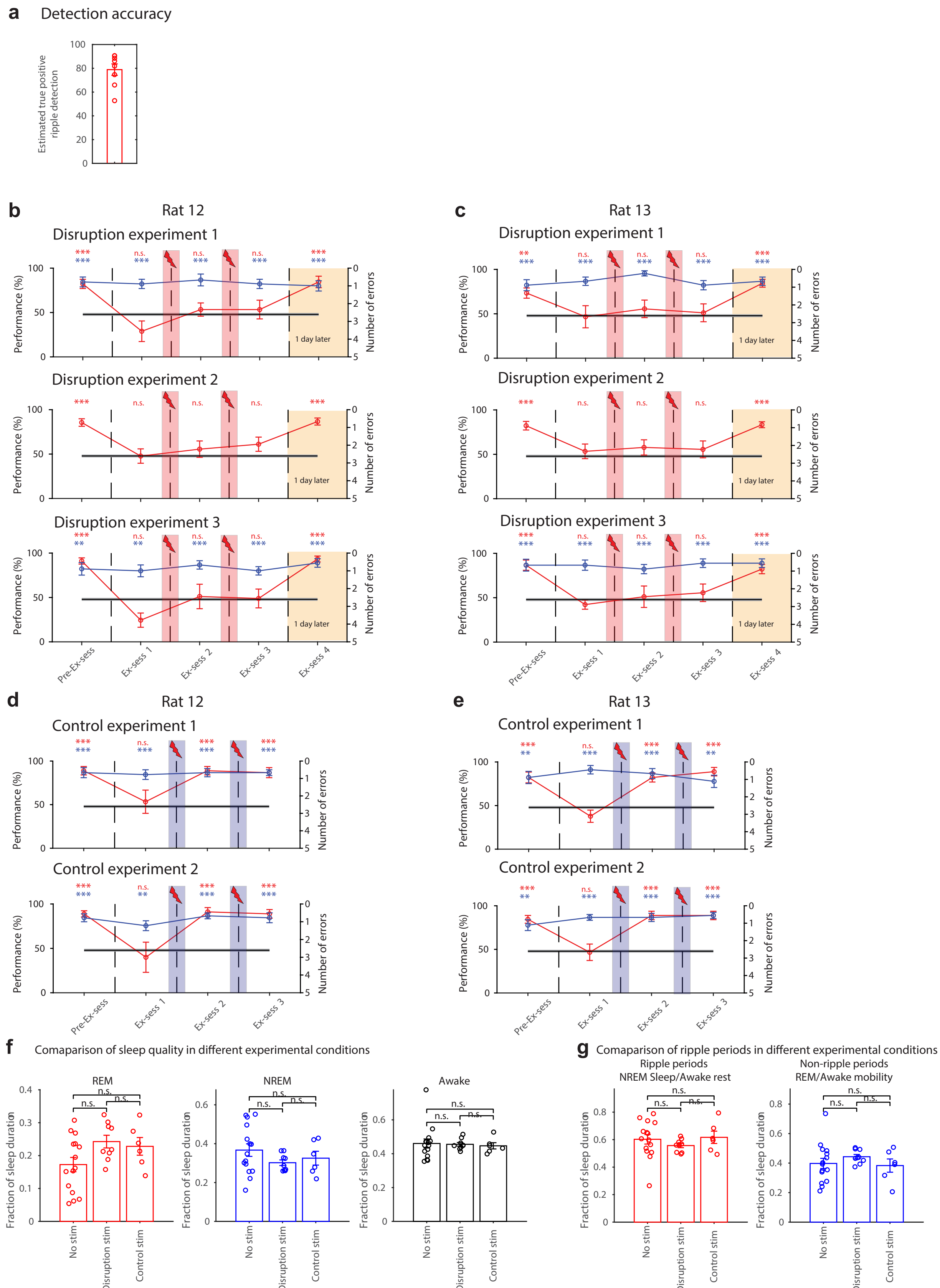

Supplementary Fig. 17: Effects of closed-loop ripple disruption. a Percentage of ripples that were detected by closed-loop ripple disruption system calculated during sleep/rest on control stimulation experiment days (N=7 sessions). To test the necessity of post-encoding SWR events to assimilation learning and task-solving we optimized the detection threshold to maximize the detection of ripple events. **b-e** Performance of Rat 12 (**b**, **d**) and Rat 13 (**c**, **e**) for changed and unchanged PAs during different experiment days (rows) for various pre-exposure and exposure sessions (each session had 3 exposures to each PAs) during ripple disruption (**b**, **c**) and control (**d**, **e**) stimulation experiments. **f** Comparison of sleep quality by categorizing post-encoding sleep into REM, NREM and Awake periods among different experimental conditions. **g** Comparison of HPC sharp wave ripple periods during post-encoding sleep among different experimental conditions. Ripple disruption stimulation did not affect the sleep quality (**f**) and the ripple-detection periods (**g**). Two-tailed one-sample t-tests in (**b-e**). N=15, 9 and 6 for no stimulation, disruption stimulation and control stimulation sessions, respectively in (**f-g**). Two-tailed Rank-sum tests in (**f-g**). Data are shown as mean±s.e.m. Source data are provided as a Source Data file. Abbreviation, Ex: Exposure, Stim: Stimulation. ns P>0.05, ***P<0.001, **P<0.01, *P<0.05.

Supplementary Table 1: Numbers of single units, L-Ratio and isolation distance on different recording days and animals in HPC and mPFC. Single unit numbers in parentheses were not included in analyses.

Rat IDs, Days	HPC units	mPFC units	L-Ratio	Isolation Distance	HPC pyramidal units	mPFC pyramidal units	HPC interneurons	mPFC interneurons
**Rat1, Day1**	75	14	0.29	8.56	62	12	13	2
**Rat2, Day1**	74	(4)	0.18	10.1	63	4	11	0
**Rat3, Day1**	(0)	42	0.47	7.17	0	30	0	12
**Rat4, Day1**	89	34	0.28	8.86	81	22	8	12
**Rat5, Day1**	119	21	0.41	8.14	103	19	16	2
**Rat1, Day2**	69	23	0.29	8.45	58	20	11	3
**Rat2, Day2**	86	(6)	0.2	9.9	74	6	12	0
**Rat3, Day2**	(0)	47	0.48	7.33	0	40	0	7
**Rat4, Day2**	117	55	0.26	8.85	106	45	11	10
**Rat5, Day2**	103	29	0.35	7.96	93	26	10	3
**Rat1, Day3**	108	19	0.22	9.5	85	14	23	5
**Rat2, Day3**	91	(4)	0.2	9.63	83	3	8	1
**Rat3, Day3**	(0)	56	0.63	6.13	0	46	0	10
**Rat4, Day3**	141	61	0.17	10.28	123	48	18	13
**Rat5, Day3**	144	34	0.3	8.64	136	25	8	9
**Rat1, Day4**	72	17	0.26	8.86	60	14	12	3
**Rat2, Day4**	59	(5)	0.23	9.31	55	5	4	0
**Rat3, Day4**	(0)	53	0.75	6.02	0	43	0	10
**Rat4, Day4**	95	44	0.3	8.67	84	34	11	10
**Rat5, Day4**	125	35	0.32	8.35	118	25	7	10
**Rat1, Day5**	84	29	0.29	8.9	67	25	17	4
**Rat2, Day5**	77	(7)	0.25	9.15	72	7	5	0
**Rat3, Day5**	(0)	44	0.96	5.14	0	40	0	4
**Rat4, Day5**	117	46	0.33	8.26	112	36	5	10
**Rat5, Day5**	166	33	0.23	9.32	161	21	5	12
**Overall**	**2011**	**762**	**0.346**	**8.4592**	**1796**	**610**	**215**	**152**
