# Supplementary material for: Offline generative network reconfiguration guides insight-like accelerated learning by assimilation into schema in rats
Source: Nat Commun. 2026 Sep 3;17:9694. doi: 10.1038/s41467-026-77318-1 (PMC13562695; doi:10.1038/s41467-026-77318-1)
Supplement: Supplementary file 2 [file 41467_2026_77318_MOESM2_ESM.pdf]

Description of Additional Supplementary Files

Supplementary Data 1: Performance comparison in experimental sessions.

Supplementary Data 2: Comparison of outcome invariances.

Supplementary Data 3: Cue induced predictions.

Supplementary Data 4: Representation dynamics during sleep.

Supplementary Data 5: Ripple modulation of positive memory related mPFC assemblies in sleep.

Supplementary Data 6: Comparison of cue responsive cell participation in mPFC assemblies and their ripple modulation.

Supplementary Data 7: Comparison of dynamics of changed PAs in mPFC frames as well as co-ordinated mPFC and HPC ensembles.

Supplementary Data 8: Strategy comparison in different sessions.

Supplementary Data 9: Place cell similarity comparison.

Supplementary Data 10: Cue-response similarity comparison.
