# Supplementary material for: Offline generative network reconfiguration guides insight-like accelerated learning by assimilation into schema in rats
Source: Nat Commun. 2026 Sep 3;17:9694. doi: 10.1038/s41467-026-77318-1 (PMC13562695; doi:10.1038/s41467-026-77318-1)
Supplement: Supplementary file 3 [file 41467_2026_77318_MOESM4_ESM.pdf]

nature portfolio

Corresponding author(s): George Dragoi

Last updated by author(s): Aug 7, 2026

Reporting Summary

Nature Portfolio wishes to improve the reproducibility of the work that we publish. This form provides structure for consistency and transparency in reporting. For further information on Nature Portfolio policies, see our Editorial Policies and the Editorial Policy Checklist.

Statistics

For all statistical analyses, confirm that the following items are present in the figure legend, table legend, main text, or Methods section.

n/a	Confirmed	
☐	☒	The exact sample size (*n*) for each experimental group/condition, given as a discrete number and unit of measurement
☐	☒	A statement on whether measurements were taken from distinct samples or whether the same sample was measured repeatedly
☐	☒	The statistical test(s) used AND whether they are one- or two-sided<br>*Only common tests should be described solely by name; describe more complex techniques in the Methods section.*
☐	☒	A description of all covariates tested
☐	☒	A description of any assumptions or corrections, such as tests of normality and adjustment for multiple comparisons
☐	☒	A full description of the statistical parameters including central tendency (e.g. means) or other basic estimates (e.g. regression coefficient) AND variation (e.g. standard deviation) or associated estimates of uncertainty (e.g. confidence intervals)
☐	☒	For null hypothesis testing, the test statistic (e.g. *F*, *t*, *r*) with confidence intervals, effect sizes, degrees of freedom and *P* value noted<br>*Give P values as exact values whenever suitable.*
☐	☒	For Bayesian analysis, information on the choice of priors and Markov chain Monte Carlo settings
☒	☐	For hierarchical and complex designs, identification of the appropriate level for tests and full reporting of outcomes
☐	☒	Estimates of effect sizes (e.g. Cohen's *d*, Pearson's *r*), indicating how they were calculated

Our web collection on statistics for biologists contains articles on many of the points above.

Software and code

Policy information about availability of computer code

Data collection	Electrophysiological and behavioral data were collected by Cheetah (Neuralynx), versions 5.6.3 and 5.7.0
Data analysis	Most of the analysis was performed in MATLAB(R2020b). Spike sorting was performed by Xclust3. Codes used in this study will be made available at: https://github.com/GDYlab/GDYlabcode

For manuscripts utilizing custom algorithms or software that are central to the research but not yet described in published literature, software must be made available to editors and reviewers. We strongly encourage code deposition in a community repository (e.g. GitHub). See the Nature Portfolio guidelines for submitting code & software for further information.

Data

Policy information about availability of data

All manuscripts must include a data availability statement. This statement should provide the following information, where applicable:

- Accession codes, unique identifiers, or web links for publicly available datasets
- A description of any restrictions on data availability
- For clinical datasets or third party data, please ensure that the statement adheres to our policy

All data needed to evaluate or extend the conclusions in the article are presented in the main article, supplementary figures, and tables. Source data are provided with this paper. All data generated in this study have been deposited into the file servers of the Yale University Medical School. The very large size of raw data prohibits their archive on public servers. The data are available under restricted access under a firewall, and access can be obtained from the corresponding author of the study. No clinical datasets or genetics data have been generated or used in this study.

Research involving human participants, their data, or biological material

Policy information about studies with human participants or human data. See also policy information about sex, gender (identity/presentation), and sexual orientation and race, ethnicity and racism.

Reporting on sex and gender	N/A
Reporting on race, ethnicity, or other socially relevant groupings	N/A
Population characteristics	N/A
Recruitment	N/A
Ethics oversight	N/A

Note that full information on the approval of the study protocol must also be provided in the manuscript.

Field-specific reporting

Please select the one below that is the best fit for your research. If you are not sure, read the appropriate sections before making your selection.

[x] Life sciences [ ] Behavioural & social sciences [ ] Ecological, evolutionary & environmental sciences

For a reference copy of the document with all sections, see nature.com/documents/nr-reporting-summary-flat.pdf

Life sciences study design

All studies must disclose on these points even when the disclosure is negative.

Sample size	No statistical methods were used to pre-determine sample sizes but our sample sizes are similar to those reported in previous publications (e.g., Liu, Sibille, Dragoi 2024 Nature Neuroscience)
Data exclusions	The criteria for each analysis were stated in the description of each analysis. One animal was excluded from the analysis of electrophysiology data from the hippocampus and one from medial prefrontal cortex due to limited detected neurons.
Replication	Experiments were performed across eleven animals. Results were replicated across animals within the study.
Randomization	Data were randomized to construct control groups in individual analysis.
Blinding	Data from five animals were used for this study, and there are no grouping or different conditions for these animals, so data collection and analysis were not performed blind to the conditions of the experiments.

Reporting for specific materials, systems and methods

We require information from authors about some types of materials, experimental systems and methods used in many studies. Here, indicate whether each material, system or method listed is relevant to your study. If you are not sure if a list item applies to your research, read the appropriate section before selecting a response.

Materials & experimental systems

n/a	Involved in the study	
[x]	[ ]	Antibodies
[x]	[ ]	Eukaryotic cell lines
[x]	[ ]	Palaeontology and archaeology
[ ]	[x]	Animals and other organisms
[x]	[ ]	Clinical data
[x]	[ ]	Dual use research of concern
[x]	[ ]	Plants

Methods

n/a	Involved in the study	
[x]	[ ]	ChIP-seq
[x]	[ ]	Flow cytometry
[x]	[ ]	MRI-based neuroimaging

Animals and other research organisms

Policy information about studies involving animals; ARRIVE guidelines recommended for reporting animal research, and Sex and Gender in Research

Laboratory animals	Long-Evans adult male rats, 3-6 months old, purchased from Charles River Laboratories USA

Wild animals	This study did not involve wild animals.
Reporting on sex	Due to the relatively high weight of the drives, only adult male rats were used in this study, a standard procedure for rats implanted with electrode drives collecting signal simultaneously from 128 channels using movable electrodes.
Field-collected samples	This study did not involve field-collected samples.
Ethics oversight	Office of Animal Research Support at Yale University

Note that full information on the approval of the study protocol must also be provided in the manuscript.

Plants

Seed stocks	N/A
Novel plant genotypes	N/A
Authentication	N/A
