# Supplementary material for: Offline generative network reconfiguration guides insight-like accelerated learning by assimilation into schema in rats
Source: Nat Commun. 2026 Sep 3;17:9694. doi: 10.1038/s41467-026-77318-1 (PMC13562695; doi:10.1038/s41467-026-77318-1)
Supplement: Supplementary file 4 [file 41467_2026_77318_MOESM5_ESM.pdf]

nature portfolio

Peer Review File

Offline generative network reconfiguration guides rodent insight-like accelerated learning by assimilation into schema

Corresponding Author: Dr George Dragoi

This file contains all reviewer reports in order by version, followed by all author rebuttals in order by version.

Version 0:

Reviewer comments:

Reviewer #1

(Remarks to the Author)
In this manuscript, the authors present an original task designed to assess how the HPC–mPFC circuit regulates the formation of newly learned, related associations that are rapidly acquired and consolidated. They report that novel paired associations entrain hippocampal activity to represent map based features, while mPFC ensembles encode the abstract rule structure. The authors further demonstrate that hippocampal ensemble activity predicts accelerated learning through coordinated interactions with the mPFC during ripples, and that disrupting ripple events abolishes this effect. Overall, the results are clear, and the primary claims are well supported by the data. I have only minor comments:
- The authors state in several places, including the abstract, that “complex de novo associative learning requires numerous encoding exposures.” While this may be broadly accurate, recent evidence suggests that the hippocampus can also form representations from single trial experiences (e.g., Chettih et al., Cell, 2024). Under such conditions, the mechanisms proposed in this manuscript may not apply, and this nuance should be acknowledged.
- Some valuable addition would be an analysis linking the observed effects to physiological metrics that might reflect underlying anatomical organization. For example, do changes in firing rate variance across conditions correlate with baseline firing rates or with burst properties of individual neurons?
- Along the same lines, the authors examine average theta power and ripple responses across conditions, but it would be informative to know whether theta modulation strength correlates with firing rate variance at the single cell level, or with anatomical position (e.g., deep vs. superficial layers)
- Several figures are difficult to read due to their small size. For instance, the panels in Figure 5 are too reduced to appreciate key details.
- There are some minor typos in the text, likely formatting etc., for instance in line 26 in page 21.

(Remarks on code availability)

Reviewer #2

(Remarks to the Author)
In this report Bhattarai and Dragoi test how information about new odor-location paired associates (PAs) become learned in the context of a previous base of related knowledge. Rat were quicker to learn new PAs than an original set. Such savings were observed when rats were given new PAs intermixed with old PA trials, and when given a completely new set. CA1 neurons showed standard mixed-selectivity with conjunctive goal and spatial coding. mPFC neurons also showed the known goal-related activity that was not sensitive to the spatial location of the goal. Blocking allocentric cues reduced performance to chance but did not change the number of CA1 cells with firing modulated by arm and goal. Bayesian decoding of CA1 activity at the odor cue revealed higher latent activation of the paired goal in sessions for which rats just learned new PAs. CA1 replay content was biased towards the newly encoded goal locations after learning. Assemblies of mPFC cells that fired during goals were more likely to be active around ripples on the same days. Closed-loop disruption of post-encoding ripples disrupted rapid acquisition of novel PAs while leaving performance on previously acquired PAs intact.

This manuscript addresses the important and understudied topic of how new information is learned upon a background of previous, related knowledge. The sophisticated behavioral paradigm, dual region recordings, and perturbation experiments

are main strengths. The primary weakness is in the presentation of the data and statements around how each data point can confirm or refute potential theories of memory assimilation. Frankly, in its present form, the manuscript was very difficult to parse with data and theory laden interpretation mixed throughout the results section, complex figures with scant legends, and run-on sentences heavy with newly invented jargon and unnecessary abbreviations.

The authors interpret their findings within three competing hypotheses: 1) a serial associative inference, which is a bit like second order conditioning; 2) map-based associative inference in which the relationships between the PAs are linked by virtue of the cognitive maps, and finally the preferred model 3) compositional inference that "would serially regroup familiar items via generative offline processes." How each model should affect the physiological and behavioral dependent measures is not clear. The serial associative inference model is discounted due to the fact that savings with 6 new PAs was equivalent to those with 3 new and 3 old. Strong assumptions must be made about the nature of how trials bias serial association. Since the 3 versus 6 new conditions only differ in terms of how recently the old were experienced.

The second hypothesis is rejected due to this data point "Similarly, in complete assimilation day, the proportion of cue-responsive cells maintaining cue-responsiveness from baseline to other sessions (i.e., new, different cues) was smaller compared to the proportion that did so from assimilation and assimilated sessions to the other assimilation sessions in HPC (i.e., same cues), and from assimilated session to the other sessions in mPFC (18.03±5.9, 9 50.05±4.9 and 47.3±6.8% of HPC cells and 31.09±5.9, 55±9.6 and 60.48±5.5% of mPFC cells for Ba-1, Asn, and Asd sessions, respectively, Figure S5I)." If my reading is correct, the authors seem to suggest that neurons in CA1 firing more similarly for an unchanged environment as compared to a changed environment means that subjects do not use cognitive maps during retrieval to make novel associations. The point exemplifies my criticism of the manuscript. 1) The theory discriminating analysis is not well motivated, 2) the relationship between the data and theory is buried in a complex, jargon-dense description of a relatively simple phenomenon, and 3) the results are over-stated to confirm the author's preferred model. More broadly, it is unclear what would falsify the claim that a schema is being engaged rather than a well-learned procedural or policy-based strategy.

I provide another example where the data presentation detract from the potential impact of the author's discovery: "The activation of a more general outcome-dependent invariant neural representation in mPFC, however, depended on the outcomes being associated with the cues as well as on the stability of the PAs. This is indicated by the reduced activation of general outcome contingencies at goals during the no-cue and complete assimilation days (Figure 2H)."
I think this sentence is referring to the high percentage of cells with variance explained by outcome in the linear analysis (and low percentage by the interaction), and the fact that this percentage is higher when cues are available and differs according to how many PAs are novel. Note that Figure 2H has 6 panels each with 4 lines. It is nearly impossible to determine which set of stars in these panels motivate the quoted sentence.

Here is another example "We hypothesized that in the map-based associative inference process the transfer of outcome representations from the goal sampling location (GL) to the cue presentation at the start arms might inferentially connect the positive outcome contingency associated with the original cue (OC) to the new cue (NC) and guide new cue-goal associated behavior (NC→GL)"
I think this is trying to say that there is a latent retrieval process at the start arm in which goals are retrieved and that common goals may link paired-associate odors that are functionally equivalent. Again, the heavy use of acronyms, and the invention of novel terms makes it difficult to parse how the model and prediction will map onto physiology.

The authors ran so many analyses, significant findings were bound to arise and the lack of model-based predictions makes it difficult to know whether an observation is spurious or essential. For example, in Figure 3M,N there is modest significance for biased goal reactivation on a subset of sessions when tested at the session level. Why should such an observation only be seen in the sessions immediately after acquisition and not during learning? Are such modest effects enough to base an entire theory? Are they reliable within subject? Supplemental Tables are referenced but not provided.

The authors report strong arm x outcome coding in CA1 during their no-allocentric control condition (Figure 2H), in which behavior is at chance. Simultaneously, they use this percentage of variance explained measure to motivate the neural underpinnings for successful behavior. How can this tension be resolved? It is possible that I have misunderstood the meaning of Ctrl 2 here as control is used elsewhere in the paper to refer to a return to the familiar 6-PA condition.

I strongly recommend that the authors motivate several discriminating analyses, eliminate all invented abbreviations from the manuscript, and focus on the most statistically compelling data points, of which there are several – the most exciting being Figure 6K (most of the Figure 6 can be moved to supplemental).

(Remarks on code availability)
The code does not show analysis, just how the data is loaded and plotted

Reviewer #3

(Remarks to the Author)
This manuscript examines the neuronal mechanisms underlying rapid schema-based associative learning, with a focus on hippocampal (HPC) and medial prefrontal cortex (mPFC) interactions. The authors introduce a novel behavioral paradigm that allows animals to rapidly replace multiple cue–place associations within an established framework. By combining this task with large-scale electrophysiological recordings and ripple-targeted closed-loop disruption, the authors aim to identify which computational strategy best accounts for the observed behavioral and neural dynamics. The study addresses an

important and timely question in systems neuroscience. However, several conceptual ambiguities, as well as methodological limitations in the task design and interpretation of the neural data, currently weaken the strength of the conclusions and warrant substantial clarification and revision.
- The manuscript is highly ambitious but exceptionally dense, and at times it feels unnecessarily difficult to follow. Already in the Introduction, different levels of explanation are mixed together — from cognitive concepts (schema, inference, insight), computational models (such as map-based inference), systems-level interactions (e.g., HPC–mPFC dynamics), and proposed cellular mechanisms (including ripple-related consolidation or assembly reactivation) — without a clear sense of hierarchy or logical progression. As it stands, the layering of concepts makes the manuscript harder to follow than necessary, and the specific hypotheses sometimes get lost in the complexity. Clarifying the key ideas and presenting them in a more structured and concise way would greatly improve readability and highlight the true contribution of the work. More generally, the manuscript repeatedly uses inflated terminology without clear justification.
- The task description is critically important for interpreting the results, yet it is difficult to follow in its current form. The combination of dense wording and highly specific nomenclature (Ba-1, Ba-2, PAs, Gr-Assim, Asn, Asd, first and second group, Ct-Assim, Allo-block, etc.) makes the experimental timeline more complex than necessary. A clearer and more structured presentation of the task phases would substantially improve readability and help highlight the conceptual advances of the study.
- On line 25 (page 6), it is unclear how the concept of pattern completion applies to the current design, given that the changed associations were not previously encoded and therefore cannot be “completed” from stored representations. More generally, this appears to be another example of inflated terminology that makes the manuscript harder to read and may not be necessary for the interpretation of the data.
- In Figure 1 I-J, performance for the unchanged PAs in blue appears to decrease during Asd blocks. How do the authors interpret this reduction? If assimilation operates through integration into a stable schema, one might expect preserved (or even enhanced) performance for unchanged associations rather than transient decrements.
- The characterization of the performance improvement as “insight-like” may be overstated. The data appear to show some progressive within-session learning during Asn (for instance in Figure 1J), followed by further improvement after sleep.
- The authors conclude that the observed changes in cue-responsive neurons discount the hypothesis of inferential replacement through feature-based associative inference. This conclusion appears also overstated. The reported analyses primarily show that cue identity representations change when cues are replaced, which is perhaps not surprising. However, serial inferential mechanism (e.g., NC->OC->GL) may not necessarily require preservation of cue-selective neuronal assemblies across sessions and could depend on more complex network dynamics beyond simple changes in firing rate (e.g., temporal coding or coordinated co-firing patterns).
- On page 9, the authors propose a mechanism termed “map-based associative inference” to explain rapid learning in the task. However, it is not entirely clear in what sense the described process constitutes inference rather than rapid associative binding within a stable spatial environment. In other words, animals may learn the general rule of the task —associating cues with goal locations within a familiar allocentric context — a strategy that naturally relies on stable allocentric cues. It is therefore unclear whether the current data necessitate a novel learning mechanism.
- Related to the previous point, while the analyses in Figures 2D–H demonstrate stable reward–location coding across sessions, it remains unclear how these findings uniquely support the proposed map-based associative inference mechanism. Stable goal–location representations in the hippocampus would be expected in a well-trained spatial task and may reflect preserved spatial and value coding rather than evidence of a distinct inferential process.
- The Bayesian decoding analyses in Figure 3 are particularly interesting and provide compelling evidence for predictive activation of goal-specific representations during cue sampling. However, it remains unclear whether such predictive activity necessarily reflects inferential computation, as opposed to rapid retrieval of newly formed cue–goal associations following consolidation. Also, although this effect is most prominent during the assimilated sessions, the cue–goal contingencies were already experienced during the preceding assimilation session and may have been progressively strengthened during that phase or the subsequent sleep phase.
- The ripple decoding analyses in Figure 4 are also very interesting, and the ripple perturbation experiment in Figure 6 is particularly impressive. However, as noted in previous points, animals were already exposed to the new PAs during the preceding assimilation session. It therefore remains unclear whether these findings require a genuinely generative network reconfiguration during sleep, rather than being parsimoniously explained by replay and consolidation of recently encoded cue–goal contingencies.
Some minor points:
- The authors state that rats first learn three “non-associative task rules” early during training that account for part of the performance improvement by day 10: (1) avoiding the non-cued start arm, (2) avoiding arms already visited within a trial (within-trial working memory), and (3) avoiding the arm rewarded in the previous trial of the block. This is a strong claim that appears somewhat peripheral to the main message of the manuscript and is not fully supported by the data presented. It remains unclear whether these behaviors reflect independently acquired abstract rules, as they overlap conceptually and seem to emerge in parallel with associative learning. The supplementary data (Figure S1A–C) show concurrent improvements but do not directly demonstrate rule acquisition. The authors should clarify this point and temper their claims accordingly.
- What fraction of the trained rats successfully acquired the task?

(Remarks on code availability)

Version 1:

Reviewer comments:

Reviewer #1

(Remarks to the Author)
In this manuscript, the authors design a complex cross-modal task in which rats learn cue-location paired associations over several weeks. This allowed rats to generate a mental schema that allowed to learn within a single day. The main findings are that hippocampal cells generate cue-location relationships to make inferential connections during the task and hippocampal along with PFC cells, reorganize their activity patterns during offline rest/sleep periods. The level of this reorganization in the hippocampus predicted learning, and this information was transferred to the PFC during ripples.

The findings are important and of high significance for the field, as they add to our knowledge of how the hippocampus and PFC coordinate to generate learning schemas.

The conclusions are supported by the data and the manuscript is written in a easy way to follow for the broad audience.

In the revised version of the manuscript, the authors have addressed all of my concerns and together with the points that they added to address all other reviewers concerns, the manuscript is ready for publication as is.

(Remarks on code availability)

Reviewer #2

(Remarks to the Author)
Through revision, the manuscript has improved in its clarity of presentation. The hypotheses are stated more clearly, however, I still feel that many analyses are not clearly grounded in the hypotheses being tested and that conclusions extend beyond the data.

I will provide some examples that illustrate a recurring logical flaw with the following structure: (A) the authors introduce a hypothesized cognitive algorithm, (B) then introduce a hypothesized implementation of that algorithm, (C) then conduct an analysis which is a proxy for that implementation, then based on that analysis, they (D) reject/accept the hypothesized cognitive algorithm.

Example 1:
A) "Our first hypothesis was that an inferential mental substitution of the original cue with the new cue, both associated with the same goal location, could occur via a neural mechanism of direct cue feature binding and transfer"
B) "One of the ways this could work is via the activation of the representation of the original cue during the encoding of the new cue-goal association at the goal location"
C) "In complete assimilation day, the proportion of cue-responsive cells maintaining cue-responsiveness from baseline to other sessions (i.e., new, different cues) was smaller compared to the proportion that did so from assimilation and assimilated sessions to the other assimilation sessions in HPC (i.e., same cues)"
D) "Therefore, when cue identities changed, network cue-responsiveness changed as well46, further discounting our first hypothesis of inferential replacement of PAs through direct cue feature binding and transfer"

The analysis simply shows that when the task demands (and stimuli) change, hippocampal and mPFC neurons fire more differently than when task demands stay the same. To say that neurons should behave identically is a strawman argument. The real comparison is whether the neural firing is more similar during assimilation than during another situation in which task demands and world stimuli are equally different, with the exception that no schema can be used to guide learning.

Example 2
A) "Our second hypothesis was that assimilation of the new PAs happened by activating and associating invariant neuronal representations linked to the task framework, namely a direct association of goal locations with the corresponding correct cues in this task"
B) "We hypothesized that these invariant neuronal representations co-activated and updated the new cue-outcome contingencies (positive, rewarded as well as negative, not rewarded) at goal locations"
C) "we observed that HPC single units that responded to reward at specific goal locations in the baseline session during learning consistently responded to the reward at the same goal location in assimilation and assimilated sessions irrespective of whether the PAs were changed or unchanged at the goal (Fig. 2d and Supplementary Fig. 6b).
D) "Despite the increased variability in outcome-invariant activations, the resulting animal behavior and HPC theta power on both these outcome types were not different between the no-cue session and the assimilation sessions of the learning days. This indicates that only during the rapid learning processes the goal-specific outcome-invariant responses inferentially linked the new cues to the goal-specific outcome, similar to the corresponding original cues."

I am not sure that a priori, one could have had an intuition for what "increased variability in outcome-invariant activations" could say about map-based inference. The analysis still needs to be better grounded. In my reading the authors find place cells (neurons that fire in an arm irrespective of what is cued there) and then make a grand theory based on that standard observation. The author analyze theta power, and then immediately following, conclude that "this indicates ..." What does "this" refer to here? That null is what needs to be clearly rejected.

In summary, I think the results are interesting but the interpretation needs to stay closer to the data and each analysis needs to be very clearly motivated about what it is testing exactly.

(Remarks on code availability)

Reviewer #3

(Remarks to the Author)
The authors have thoroughly addressed my concerns. I have no further comments and support publication.

(Remarks on code availability)

Open Access This Peer Review File is licensed under a Creative Commons Attribution 4.0 International License, which permits use, sharing, adaptation, distribution and reproduction in any medium or format, as long as you give appropriate credit to the original author(s) and the source, provide a link to the Creative Commons license, and indicate if changes were made.
In cases where reviewers are anonymous, credit should be given to 'Anonymous Referee' and the source.
The images or other third party material in this Peer Review File are included in the article's Creative Commons license, unless indicated otherwise in a credit line to the material. If material is not included in the article's Creative Commons license and your intended use is not permitted by statutory regulation or exceeds the permitted use, you will need to obtain permission directly from the copyright holder.
To view a copy of this license, visit https://creativecommons.org/licenses/by/4.0/

REVIEWER COMMENTS

Reviewer #1 (Remarks to the Author):

In this manuscript, the authors present an original task designed to assess how the HPC–mPFC circuit regulates the formation of newly learned, related associations that are rapidly acquired and consolidated. They report that novel paired associations entrain hippocampal activity to represent map based features, while mPFC ensembles encode the abstract rule structure. The authors further demonstrate that hippocampal ensemble activity predicts accelerated learning through coordinated interactions with the mPFC during ripples, and that disrupting ripple events abolishes this effect. Overall, the results are clear, and the primary claims are well supported by the data. I have only minor comments:

We thank the reviewer for appreciating our study and its findings. We think our reviewer's feedback helped us improve the manuscript as we added a set of valuable new results, which are described in detail in our subsequent responses.

The authors state in several places, including the abstract, that "complex de novo associative learning requires numerous encoding exposures." While this may be broadly accurate, recent evidence suggests that the hippocampus can also form representations from single trial experiences (e.g., Chettih et al., Cell, 2024). Under such conditions, the mechanisms proposed in this manuscript may not apply, and this nuance should be acknowledged.

We thank the reviewer for raising this important point, about multiple types of rapid learning likely supported by different neuronal mechanisms. In our task, rats acquired a set of 6 paired associates (PAs) over the span of multiple weeks. We developed this complex 6 PAs association task that required several weeks of training for animals to learn. However, as our reviewer points out, this type of trained association behavior in rats requiring learning of numerous concurrent associations is fundamentally different from some innate behaviors in birds such as food-caching chickadees, where one shot memory retrieval can be achieved after a single-trial experience. In the neuroscience literature, one-shot retrieval of innate memory responses have also been reported in the contextual fear conditioning paradigms in rodents. We believe that one key distinction of our task from the one-shot memories formed and retrieved in the bird as well as in some innate contextual-fear conditioning tasks in rodents is that, aside from being innate behaviors, these are all based on episodic experiences (i.e. what happened where and when). The salience of these individual memories, either innately through their wiring of the brain or through the strong fearful nature of emotional responses these produce, makes them peculiarly memorable and retrievable. In addition, most of these rapid learning processes are tested using single associations, not numerous distinct experiences in the same learning session. However, in our task the complex and concurrent learning of 6 PAs takes many weeks, which forms a schema of these associations' rules. When animals were given new associations in the same context, they retrieved these schema rules and applied them rapidly during the new experiences to learn them much quicker. Therefore, this kind of rapid learning of multiple associations concurrently is more like the day-to-day human learning experiences based on the framework/abstract-rules formed during the lifetime of prior experiences in the similar context. We now discuss this distinction in the Discussion section of the revised manuscript: "Our cross-modal 6-PAs associative schema rules, which required 6-8 weeks of training to acquire and later accelerated learning of multiple new similar associations, is distinct from memory retrieval of single, innate, episodic-like learning and natural behaviors such as food-caching in chickadee birds[1] or context-specific associative fear conditioning in rodents. We believe that one key distinction in our paradigm compared to one-shot memories formed and retrieved in birds and in some innate contextual-fear conditioning tasks in rodents is that, aside from being innate behaviors, these tasks are all based on *episodic* experiences (i.e., what happened where and when). The salience of these individual memories, either innately through their wiring in the brain or through the strong fearful nature of emotional responses these produce, makes them

peculiarly memorable and retrievable. In addition, most of these rapid learning processes are tested using single associations, not numerous distinct experiences in the same learning session. However, in our paradigm, the complex and concurrent learning of 6 PAs takes many weeks, which forms a schema of these *semantic* associations. Learning of the new PAs would then be accelerated using the rules based on this schema."

Some valuable addition would be an analysis linking the observed effects to physiological metrics that might reflect underlying anatomical organization. For example, do changes in firing rate variance across conditions correlate with baseline firing rates or with burst properties of individual neurons?

We appreciate our reviewer's suggestion to analyze the physiological-level single unit firing rate properties in relation to the task-related firing rate variances observed in our results. According to the specific suggestions by the reviewer, we conducted several analyses to better understand how neuronal baseline firing rates and burst properties changed during different sessions. We have now added Supplementary Fig. 7 showing these new results as follows:

1. We compared firing rates in different sessions and did not observe systematic changes across sessions (Supplementary Fig. 7a).
2. We calculated the neuronal burst indices, and they were not different across sessions (Supplementary Fig. 7b).
3. We calculated correlations between firing rates in different learning sessions with the baseline firing rates. The correlations were high but reduced with the passage of time between sessions and were not systematically different between learning and control experiment conditions (Supplementary Fig. 7e).
4. We calculated the correlations of single unit firing rates with the explained task-related variance (outcome/outcome at specific goals) in both HPC and mPFC. The similarity values were largely distributed but mean firing rates did not significantly relate to the task-related variances observed in our results (Supplementary Fig. 7f).
5. We calculated the correlations of the burst indices of single units with the explained task related variance (outcome/outcome at specific goals) in both HPC and mPFC. We found that mPFC single units had positive relationship between burst indices and task-related firing rate variances across multiple session types (Supplementary Fig. 7g, j).

Along the same lines, the authors examine average theta power and ripple responses across conditions, but it would be informative to know whether theta modulation strength correlates with firing rate variance at the single cell level, or with anatomical position (e.g., deep vs. superficial layers)

We appreciate the suggestions and agree with the reviewer that studying basic single unit properties such as their anatomical position and their theta modulation strengths during rapid-learning experiment sessions would be interesting. However, we regret to inform the reviewer that we are limited in our ability to accurately identify the anatomical position of the single units because we recorded the current dataset using multiple tetrodes in HPC and mPFC regions with limited and uncertain access to their discrete sublayer organization. Despite not being able to address the interesting question of whether there were differences in deep vs. superficial single units, we conducted the following new analyses studying the theta modulation of single units, and added them as panels in the revised Supplementary Fig. 7:

1. We calculated the theta modulation indices and fraction of theta modulated single units in HPC and mPFC, which were not different between sessions (Supplementary Fig. 7c, d).
2. We calculated the correlations of the theta modulation strengths with the explained task related variance (outcome/outcome at specific goals) in both HPC and mPFC. We found

that HPC single units displayed negative correlations between theta phase locking and task-related firing rate variances across multiple session types (Supplementary Fig. 7h). This was also true for the task-related firing rate variances between different goals (arm IDs) at goal locations and for the outcome and arm IDs in the inbound movement trajectories (Supplementary Fig. 7i, k). In summary, the more the neurons were phase locked to theta, the less likely they were to display task-related firing rate variances both at goal and during inbound movement trajectories.

In the revised manuscript we added sentences describing these results: "Next, we studied the relationship of the basic properties of single units with these task-related variances. We found that mPFC single unit burst indices were positively correlated, and HPC single unit theta phase modulations were negatively correlated, with the task-related variances in single unit firing rates during learning (Supplementary Fig. 7)."

Several figures are difficult to read due to their small size. For instance, the panels in Figure 5 are too reduced to appreciate key details.

We apologize for this inconvenience. We agree with the reviewer regarding the importance of figure legibility for our readers. In addressing the specific comment made by reviewer, we increased the size of all panels and fonts in Fig. 5 to make them more legible. In doing so, in this revision, we moved several panels from Fig. 5 to Supplementary Fig. 16, which we believe reduced their crowdedness and distraction from the more important findings in Fig. 5, that we want the readers to focus on. We also tried improving other figure panels (e.g., Fig. 2) for legibility. As an example, in the revised manuscript, we have divided the crowded panels of original Fig. 2h into two panels (revised Fig. 2g and Fig. 2h) showing the effects of learning on temporal and spatial representations separately. We have also moved Fig. 2d from the previous version of our manuscript to Supplementary Fig. 6d to focus readers on the more important findings we reported in Fig. 2.

Additionally, in response to the similar suggestions made by the other two reviewers on how best to improve the readability, we removed unnecessary abbreviations in the main, text such as Gr-Assim, Ctrl 1, Ctrl 2 and Ct-Assim. While doing that, we also refrained from interpreting the results in broad terminologies that were inherited from literature such as sensory binding, pattern completion and path integration. Similarly, to avoid confusion and make it easier to parse we removed other abbreviations such as NC, OC and GL and the equations that we used in relation to the description of the hypotheses, such as NC→GL and GL→NC→GL.

There are some minor typos in the text, likely formatting etc., for instance in line 26 in page 21.

We have now taken care of the typos wherever we found them, including the specific instance mentioned by the reviewer.

Reviewer #2 (Remarks to the Author):

In this report Bhattarai and Dragoi test how information about new odor-location paired associates (PAs) become learned in the context of a previous base of related knowledge. Rat were quicker to learn new PAs than an original set. Such savings were observed when rats were given new PAs intermixed with old PA trials, and when given a completely new set. CA1 neurons showed standard mixed-selectivity with conjunctive goal and spatial coding. mPFC neurons also showed the known goal-related activity that was not sensitive to the spatial location of the goal. Blocking allocentric cues reduced performance to chance but did not change the number of CA1 cells with firing modulated by arm and goal. Bayesian decoding of CA1 activity at the odor cue revealed higher latent activation of the paired goal in sessions for which rats just learned new PAs. CA1 replay content was biased towards the newly encoded goal locations after learning. Assemblies of mPFC cells that fired during goals were more likely to be active around ripples

on the same days. Closed-loop disruption of post-encoding ripples disrupted rapid acquisition of novel PAs while leaving performance on previously acquired PAs intact.

We thank the reviewer for providing invaluable feedback. We are encouraged by the reviewer's comments that have been constructive and helped us improve the manuscript. We also appreciate that the reviewer has provided a comprehensive summary of our main findings.

We agree that the blocking of the allocentric cues reduced performance to chance and did not reduce invariance of HPC neural activity (similarity with the baseline sessions before and after) explained by outcome at goal. However, blocking allocentric cues reduced the invariance of HPC neural activity (similarity with the baseline sessions before and after) explained by the outcome in the spatially binned firing rates during the inbound movement trajectory, i.e. when rats moved from goals to the center (original and revised figure Fig. 2h). We believe the rats activated the known outcome code (from baseline) in their temporal goal representations at the goal during allocentric cue blocking session, once they correctly found the goal. But, due to the alterations in the allocentric cues, this representational generalization/invariance for associated outcome was impaired in the spatial representations when rats took the inbound movement trajectory after sampling the goal, as shown in Fig. 2h. On both the no-cue and allocentric-blocking sessions, the levels of spatial invariance were similar, systematically lower than observed during all assimilation learning experiments. This result is also presented in the result section: "The goal-specific invariance explained by outcome was also reduced during spatial representations during inbound movement in allocentric-block control days, suggesting its dependence on the stability of allocentric-context/map (Fig. 2h)."

This manuscript addresses the important and understudied topic of how new information is learned upon a background of previous, related knowledge. The sophisticated behavioral paradigm, dual region recordings, and perturbation experiments are main strengths.

We appreciate the reviewer's highlighting of the major strengths of our study.

The primary weakness is in the presentation of the data and statements around how each data point can confirm or refute potential theories of memory assimilation. Frankly, in its present form, the manuscript was very difficult to parse with data and theory laden interpretation mixed throughout the results section, complex figures with scant legends, and run-on sentences heavy with newly invented jargon and unnecessary abbreviations.

We appreciate the reviewer's highlighting of the major weaknesses of our study. To improve the manuscript by incorporating the reviewer's suggestions, we made the following changes in the revised manuscript:

1. We removed the unnecessary abbreviations in the main text such as Gr-Assim, Ctrl 1, Ctrl 2 and Ct-Assim. We now use abbreviations only when space is constrained in the figures along with the brief elaboration of what they mean in the figure captions.
2. We also removed some poorly formed and broadly interpreted hypotheses such as *feature-based associative inference* and now only use narrower and better supported hypothesis i.e. *direct cue feature binding and transfer* which is more directly supported by the results.
3. In the same spirit, we removed the interpretation of our result as refuting the pattern completion hypothesis. In the revision we removed the sentence: "This discounts our *first hypothetical mechanism* that some original PAs acted as the framework of relational declarative facts and were necessary to facilitate assimilation of changed PAs through *pattern completion*".
4. While doing that, we also refrained from interpreting the results using broad terminologies that were inherited from literature such as sensory binding, pattern completion and path integration.

5. Similarly, to avoid confusion and make it easier to parse, we removed other abbreviations such as NC, OC and GL and the equations that we used in relation to the description of the hypotheses, such as NC→GL and GL→NC→GL.

The authors interpret their findings within three competing hypotheses: 1) a serial associative inference, which is a bit like second order conditioning; 2) map-based associative inference in which the relationships between the PAs are linked by virtue of the cognitive maps, and finally the preferred model 3) compositional inference that "would serially regroup familiar items via generative offline processes." How each model should affect the physiological and behavioral dependent measures is not clear.

We thank the reviewer for correctly pointing out the hypotheses that we introduced in the Introduction section: 1) a serial associative inference or *direct cue feature binding and transfer,* 2) map-based associative inference in which the relationships between the PAs are linked by virtue of the cognitive maps, and 3) compositional inference that would serially regroup familiar items via generative offline processes.

However, at some point in the original Results section, we added one more relatively broad interpretation, namely "the pattern completion" also called 'the first hypothesis', which affected the overall numbering of our 3 original hypothesis and possibly created confusion among which hypothesis from the Introduction section we were referring during our interpretation of the Results. We have now removed the "pattern completion" hypothesis and corrected the numbering of the hypotheses that we tested for or against the observed evidence in the result section to agree with our reviewer's list.

In the revised manuscript, the hypotheses in the Results section are aligned with the ones we introduced earlier in the main text. We again thank the reviewer for providing us with an important criticism to substantially improve the readability of the manuscript.

The serial associative inference model is discounted due to the fact that savings with 6 new PAs was equivalent to those with 3 new and 3 old. Strong assumptions must be made about the nature of how trials bias serial association. Since the 3 versus 6 new conditions only differ in terms of how recently the old were experienced.

We thank the reviewer for this criticism. This was primarily due to an oversight on our part. The reviewer correctly noted that we showed "*that savings with 6 new PAs was equivalent to those with 3 new and 3 old",* which we used to refute the pattern completion hypothesis that we incorrectly termed as 'the first hypothesis'. Based on this result, if none of the original PAs were needed for the rapid assimilation of newer PAs, we believed rats might not be relying on the remaining original associations to complete the relational pattern and learn the new ones. However, we incorrectly referred to this hypothesis as 'the first hypothesis' (meaning the pattern completion hypothesis), which was also how we referred to "the serial associative inference" in the Introduction section.

In the revised manuscript we have removed the pattern completion hypothesis altogether from interpretation of this result. Similarly, in the revised manuscript, we refer to the serial associative inference in its relatively specific interpretation as *cue feature binding and transfer* based on the results (also discussed in the reviewer's subsequent comment) that support refuting the simple repurposing of the original cue coding cells during coding of the newly learned cues.

The second hypothesis is rejected due to this data point "Similarly, in complete assimilation day, the proportion of cue-responsive cells maintaining cue-responsiveness from baseline to other sessions (i.e., new, different cues) was smaller compared to the proportion that did so from assimilation and assimilated sessions to the other assimilation sessions in HPC (i.e., same cues), and from assimilated session to the other sessions in mPFC (18.03±5.9, 9 50.05±4.9 and 47.3±6.8% of HPC cells and 31.09±5.9, 55±9.6 and 60.48±5.5% of mPFC cells for Ba-1, Asn, and Asd sessions, respectively, Figure S5I)." If my reading is correct, the authors seem to suggest that neurons in CA1 firing

more similarly for an unchanged environment as compared to a changed environment means that subjects do not use cognitive maps during retrieval to make novel associations.

We again thank the reviewer for the valid criticism that we believe resulted from our misnumbering the hypotheses from Introduction to the Results sections. The second hypothesis in the Introduction section referred to *the map based associative inference*. However, in this section we erroneously referred to *serial associative inference* model as 'the second hypothesis', which we attempted to dispel using this data.

Thanks to the reviewer, in the revised manuscript, we corrected that this data point correctly now refers to the first hypothesis of serial associative inference model and also restricted the interpretation to only the *cue feature binding and transfer,* while leaving open the possibility of other forms of serial associative inference that could take place while networks code for the features in this task.

The reviewer is correct in the interpretation that this data shows that subjects do not use the same cue coding network framework in representing the corresponding new cues associated to the same goal and this does not disprove the use of cognitive maps in the rapid learning process.

In the revised manuscript, we present data and results supporting the use of cognitive maps with invariant spatial as well as temporal outcome generalizing properties claiming that the *map-based associative inference*, our second hypothesis, is the most likely HPC mechanism at play during this rapid learning process.

In the revised Discussion section we state, "Therefore, our results support a *map-based associative inference* model[2,3] and failed to detect evidence for *direct cue feature binding and transfer* [4,5] in guiding this insight-like accelerated learning of 3-6 new PAs simultaneously after one encoding and one offline sleep/rest session." This is also now stated throughout the Results section.

The point exemplifies my criticism of the manuscript. 1) The theory discriminating analysis is not well motivated, 2) the relationship between the data and theory is buried in a complex, jargon-dense description of a relatively simple phenomenon, and 3) the results are over-stated to confirm the author's preferred model.

In response to the three points mentioned by our reviewer, in the revised manuscript:

1) We have now corrected the motivation for the proper theories discriminated against by the proper analyses.
2) Removed the broader interpretations and stated the essential and directly linked interpretations.
3) Better aligned the hypothesis with the results of the tests.

More broadly, it is unclear what would falsify the claim that a schema is being engaged rather than a well-learned procedural or policy-based strategy.

We appreciate the reviewer for providing us with an opportunity to clarify the main findings of this study by asking what makes this rapid learning a schema-based process rather than a well learned procedural or policy-based strategy. In response, we present the following points specific to this task and how these were defined as essential characteristics of the testable mental schema task:

1) The schema in this task refers to the multiple parallel associations of cue-outcome contingencies (positive and negative outcomes related to different associated as well as non-associated cues to the specific locations) at multiple locations, which can be replaced

in parallel while keeping the same contingencies at those locations. Besides defining the schema, we have also observed the other essential features of schema as posited in the schema literature[6-9], which we explain further in our point no. 2) below.

2) This framework was i) flexible and dynamic, enabling repeated assimilation of group and complete sets of new PAs across days enabling successful problem solving, ii) stable in absence of new learning over several days (i.e., rats performed well after days in the baseline sessions using newly learned PAs), iii) dependent on the allocentric-context in which it was originally established (i.e., rules were only applied in a specific context), iv) non-episodic and without serial position effect (i.e., rules were used for all cues irrespective of their identities and in parallel for all 3-6 PAs; Supplementary Fig. 4), and v) not active when rats employed non-associative strategies (i.e., procedural rules such as reward history memory during no-cue and allocentric block control experiments).
3) The rats were unable to achieve optimal performance using alternative policy-based strategies that were structurally present in the task. Rats learned early in the training policy-based strategies such as not entering the start arms, remembering the recent PAs already rewarded in the block (trials with the same PAs were not repeated within a 6-trial block) and selecting the goals that were unrewarded in the block (i.e., reward history memory, Supplementary Fig. 1h-k). Although we observed that our rats applied these policy strategies when cues were absent in the no-cue sessions and when context was altered in the allocentric blocking experiment sessions, they could not achieve the optimal/significant performance when using only these rules. We think that a framework of multiple 6 associations that we refer to as schema could not be simply interpreted as a policy. However, our definition of schema does not exclude a policy that allows rats to remember goal associated rules, i.e., one correct cue leads to reward at only one goal location, while multiple incorrect cues lead to no reward at the same goal location, rule which was applied to form 6 outcome contingencies in parallel.
4) There were also controls for procedural/habitual strategies that we built into the task. For example, the natural behaviors in rats could allow them to remember egocentric movement sequences and retrieve correct rewards independent of correctly associating the PAs to retrieve rewards at correct goals. In our task paradigm, the start arms alternated in every subsequent block (after 6 trials), which restricted animals from remembering egocentric movement sequences in retrieving reward from the correct goals, because animals started from a different start arm and movement sequences for the goals were completely different.

We believe that through repeated experiments using 3 and 6 new PAs during learning experiments and controlling for the non-associative aspects of the task in the control experiment sessions we were able to establish that fundamental aspects of this task better align with the schema based mental frameworks as defined in the literature[6,9] and implemented in the seminal papers[7,8].

I provide another example where the data presentation detract from the potential impact of the author's discovery: "The activation of a more general outcome-dependent invariant neural representation in mPFC, however, depended on the outcomes being associated with the cues as well as on the stability of the PAs. This is indicated by the reduced activation of general outcome contingencies at goals during the no-cue and complete assimilation days (Figure 2H)."
I think this sentence is referring to the high percentage of cells with variance explained by outcome in the linear analysis (and low percentage by the interaction), and the fact that this percentage is higher when cues are available and differs according to how many PAs are novel.

Yes, the reviewer is correct in the interpretation that larger percentage of variance in mPFC neuronal ensemble were explained by the outcome irrespective of the goal during learning and

depends on how many PAs were changed. In the revised manuscript, we clarified this statement.

Note that Figure 2H has 6 panels each with 4 lines. It is nearly impossible to determine which set of stars in these panels motivate the quoted sentence.

We apologize to the reviewer for the larger number of panels included in Figure 2H. We agree with the reviewer that there were 6 panels in Figure 2H, which had multiple comparisons. To better organize these results, during revision, we have broken up the original Figure 2H into two Figure panels 2g and 2h, now showing outcome generalization/invariance results in the temporal and spatial firing domains, respectively. In the revised Fig. 2g, the right sub-panel now shows the comparison of the variances in ensemble similarities among different sessions on different experiment days explained by outcome irrespective of the goals. In the revised manuscript, we also now use improved figure legends, referring to the experiment days by their respective names instead of using abbreviations like Ctrl 1, Ctrl 2 etc. While revising Fig. 2, we have also moved original Figure 2D to revised Supplementary Fig. 6d because we realized that within session analyses of different task related variables in HPC and mPFC are not directly conducive to the important observations made in Fig. 2.

Here is another example "We hypothesized that in the map-based associative inference process the transfer of outcome representations from the goal sampling location (GL) to the cue presentation at the start arms might inferentially connect the positive outcome contingency associated with the original cue (OC) to the new cue (NC) and guide new cue-goal associated behavior (NC→GL)"
I think this is trying to say that there is a latent retrieval process at the start arm in which goals are retrieved and that common goals may link paired-associate odors that are functionally equivalent. Again, the heavy use of acronyms, and the invention of novel terms makes it difficult to parse how the model and prediction will map onto physiology.

We thank our reviewer for making the correct interpretation of this hypothesis and suggesting improvements in presentation of the results. In line with the review's suggestions, in the revised manuscript, we have removed the abbreviations and adjusted to the reviewer's view to explain this hypothesis. Now the revised Results section related to this hypothesis reads "We hypothesized that in the map-based associative inference process the activation of correct goal representations during the cue presentation at the start arms might inferentially guide the new cue-goal associated behavior. In this scenario, we would expect to observe activation of the associated outcome-specific goal representations when the rats sampled the correct cues at the start arms.".

The authors ran so many analyses, significant findings were bound to arise and the lack of model-based predictions makes it difficult to know whether an observation is spurious or essential. For example, in Figure 3M,N there is modest significance for biased goal reactivation on a subset of sessions when tested at the session level. Why should such an observation only be seen in the sessions immediately after acquisition and not during learning? Are such modest effects enough to base an entire theory? Are they reliable within subject? Supplemental Tables are referenced but not provided.

To address our reviewer's concern that these analyses did not adhere to the model, we reiterate that there were two predictions that we tested in relation to the map-based inference model (the second hypothesis).

1. There would be invariant/generalized representation of outcome at specific goals.
2. The activation of latent goal specific representations at cue presentation might lead to correct new cue-goal associated behavior.

Therefore, these results and the effect sizes observed in these analyses (Fig. 3m, n) would agree with both predictions of this hypothesis (Figs. 2, 3).

We tested this hypothesis (second prediction from above) in different datasets as these sessions were categorized into different stages of learning or the control experiment sessions. Because these tests were conducted on different datasets against their own respective chance levels (obtained by shuffling the decoded probabilities), we did not see a need for multiple comparisons or corrections in the model.

To address the concern regarding the modest effect/significance, the p-value for the signed-rank test for the session level effect was $P=6.0 \times 10^{-4}$. To further address the reviewer's concern that these results might only be modest and appear only in the session level dataset, we conducted prediction analyses for each trial, conducted signed-rank test for the comparison against corresponding shuffles and found the results significant for assimilated session with p-value of $P=5.8 \times 10^{-4}$. Also, we found that these predictions were significantly greater than in the baseline session by conducting rank-sum test with p-value of 9.4×10^{-4}. We included these results in the revised Supplementary Fig. 11g. The observation that the predictions at start arm during cue presentation were significant immediately after learning and not during can be interpreted as an inferential activation of the latent causes associated with the positive action-toward-goal, which likely guided the correct behavior during rapid inferential learning. The fact that we see significant predictions in correct trials (zero error) but not during incorrect trials in the assimilated sessions suggests these are possibly linked to correct association-related behavior, i.e., inference after new learning. These significant predictions also coincided with the significant improvement in animals' performance after new learning. Meanwhile, during the assimilation session when we did not observe neural correlates for these predictions, the correct cue-goal associated behavior was not significantly above chance levels. Thus, improvement in animals' performance after new learning was linked to these predictions in the correct trials. In addition, we did not observe the predictions of next goal choices (possible planned visit) during incorrect trials even in the assimilated sessions, suggesting these were not primarily representing upcoming choice or plan.

To address the reviewer's question of reliability within subjects, we calculated trial by trial predictions and corresponding shuffles for each animal separately. We found that the predictions were higher than shuffles in 3 out of 4 animals that had HPC single unit recordings. In two animals, p-values were significantly greater than in shuffles with $P=8.2 \times 10^{-3}$ and 0.02. In another animal p-value was $P=0.08$, which was tending toward significance, and was significant in comparison to the baseline predictions ($P=0.027$). We included these results in the revised manuscript in Supplementary Fig. 11e, f. We have added the following text in the revised Results section to include these results: "The predictive activation of future correct goals was higher than shuffles in trial-wise comparison overall and within subjects (3 out of 4 animals, Supplementary Fig. 11e-g)."

We apologize to the reviewer for omitting to include the Supplementary tables that we mentioned in the captions. In the revision, we have uploaded the revised supplementary statistics with the Supplementary table file.

The authors report strong arm x outcome coding in CA1 during their no-allocentric control condition (Figure 2H), in which behavior is at chance. Simultaneously, they use this percentage of variance explained measure to motivate the neural underpinnings for successful behavior. How can this tension be resolved? It is possible that I have misunderstood the meaning of Ctrl 2 here as control is used elsewhere in the paper to refer to a return to the familiar 6-PA condition.

Our reviewer is correct in pointing out there is stronger arm x outcome coding invariances during Ctrl 2 (Allocentric cues blocking) experiment. However, the comment made by reviewer is true only at goal sampling periods, in which we analyzed the temporal goal related representations. We have now split Fig. 2H into two different panels Fig 2g and 2h, which we

believe will also address the concern from the reviewer. In revised Fig. 2h left and middle panels, we show that the invariances related to outcome as well as outcome at specific goals were diminished in the spatial representations during the inbound movement trajectory (goal to the center) during the Allocentric cue blocking day. These are at the same levels as no-cue control test experiment days and are significantly smaller than the assimilation learning days. We think successful behavior would require these neural underpinnings both at the goal and in the spatial representations following the goal. This feature (percentage of variance) measures the similarity of outcome representations of the allocentric cue blocking session with the outcome representations in baseline and return-to-baseline sessions (familiar 6 PA conditions) that animals also performed on the same experiment day. Even if successful inferential associations could be made at goal, without the generalization of outcome in the spatial firing rate map (observed during all assimilation learning days), rats were unable to perform successfully. Hence, this deficit might interfere with the accurate outcome updates in relation to the allocentric cognitive map. This result is also presented in the Results section: “The goal-specific invariance explained by outcome was also reduced during spatial representations during inbound movement in allocentric-block control days, suggesting its dependence on the stability of allocentric context/map (Fig. 2h).”

I strongly recommend that the authors motivate several discriminating analyses, eliminate all invented abbreviations from the manuscript, and focus on the most statistically compelling data points, of which there are several – the most exciting being Figure 6K (most of the Figure 6 can be moved to supplemental).

We wholeheartedly appreciate the reviewer’s suggestions. We believe we were able to address most of the concerns that the reviewer raised. In addressing the reviewer’s and the other reviewers’ comments, we believe in the revised manuscript we avoided the previously distracting claims, hypotheses, and interpretation of our results. We consider this revision has improved the paper significantly and highlighted our important findings. We also appreciate the reviewer appreciating the ripple disruption experiments in Fig. 6. However, we respectfully hesitate to move the results of Fig. 6 to a supplementary figure, because we believe the panels could be informative to the broader group of readers who might implement such procedures to disrupt ripple related activities in the rodent brain.

Reviewer #2 (Remarks on code availability):

The code does not show analysis, just how the data is loaded and plotted

We have included a helper folder with the necessary codes and functions for calculating the data (.mat files) that were used for loading and plotting the figures.

Reviewer #3 (Remarks to the Author):

This manuscript examines the neuronal mechanisms underlying rapid schema-based associative learning, with a focus on hippocampal (HPC) and medial prefrontal cortex (mPFC) interactions. The authors introduce a novel behavioral paradigm that allows animals to rapidly replace multiple cue–place associations within an established framework. By combining this task with large-scale electrophysiological recordings and ripple-targeted closed-loop disruption, the authors aim to identify which computational strategy best accounts for the observed behavioral and neural dynamics. The study addresses an important and timely question in systems neuroscience. However, several conceptual ambiguities, as well as methodological limitations in the task design and interpretation of the neural data, currently weaken the strength of the conclusions and warrant substantial clarification and revision.

We thank the reviewer for providing constructive feedback. We appreciate that our reviewer finds the questions we addressed in the manuscript timely and important. Similarly, we also

appreciate our reviewer for pointing out the weaknesses in the manuscript, which we have attempted to address in the following sections as well as in the revised manuscript.

The manuscript is highly ambitious but exceptionally dense, and at times it feels unnecessarily difficult to follow. Already in the Introduction, different levels of explanation are mixed together — from cognitive concepts (schema, inference, insight), computational models (such as map-based inference), systems-level interactions (e.g., HPC–mPFC dynamics), and proposed cellular mechanisms (including ripple-related consolidation or assembly reactivation) — without a clear sense of hierarchy or logical progression. As it stands, the layering of concepts makes the manuscript harder to follow than necessary, and the specific hypotheses sometimes get lost in the complexity. Clarifying the key ideas and presenting them in a more structured and concise way would greatly improve readability and highlight the true contribution of the work. More generally, the manuscript repeatedly uses inflated terminology without clear justification.

We agree with the reviewer's comments that the level of complexity that we attempted to parse through, while keeping the focus on the core hypotheses of rapid associative learning throughout the paper, could be overwhelming. However, with the help of reviewer's suggestions, during this revision, we have made substantial changes in the way we put forward the hypotheses and how we tested and interpreted the results. In the Introduction, we first defined and discussed the accelerated learning in the schema framework. We then introduced three hypotheses that could explain the rapid associative learning in this framework.

1) Serial associative inference. We tested this hypothesis in our task by testing one of the predictions that same cue-coding representations repurposed to code for the newly introduced equivalent cues. The narrow prediction of this hypothesis is now named *cue feature binding and transfer.* To make this strictly adhere to what we tested, we removed the previous, broader hypothesis terminology of *feature based associative inference.*
2) Map-based associative inference. We have tested and interpreted this hypothesis by investigating two different predictions as shown in the Results section. First, whether and how the inferential representations activate at specific goals during learning and second, whether after learning we see inferential activations of goal predictive representations guiding correct cue-goal behavior.
3) Compositional inference. We primarily tested this hypothesis through cross-modality (cue-location) coactivations in HPC during offline states following new learning. There were several facets to this hypothesis. First, we tested whether new cue-location pairings would be activated in the HPC. Second, how these compositional co-activities affected the network in mPFC and the interaction between HPC-mPFC. Third, whether we would observe the systems-level changes in coactivations two days after learning, besides the rapid learning-related network reconfigurations. Fourth, whether these ripple-related reconfigurations were causally linked to the rapid associative learning.

Now in the revised manuscript, our study focuses on these three core hypotheses. We have removed several other vague hypotheses such as *pattern completion.* In doing so, we also numbered our hypotheses (the three that we introduced above) in the Results section to match the ones we described earlier in the Introduction section. Similarly, we removed vague terminologies adapted from literature such as sensory binding, and path integration in the interpretation of the results. To avoid confusion and improve readability of our manuscript, we removed other abbreviations such as NC, OC and GL and the equations that we used to describe the hypotheses, such as NC→GL and GL→NC→GL. Similarly, we removed abbreviations in the Main text such as Gr-Assim, Ctrl 1, Ctrl 2 and Ct-Assim. We now use them only when space is constrained in the figures along with a brief description in the captions.

The task description is critically important for interpreting the results, yet it is difficult to follow in its current form. The combination of dense wording and highly specific nomenclature (Ba-1, Ba-2, PAs, Gr-Assim, Asn, Asd, first and

second group, Ct-Assim, Allo-block, etc.) makes the experimental timeline more complex than necessary. A clearer and more structured presentation of the task phases would substantially improve readability and help highlight the conceptual advances of the study.

We again thank the reviewer for this constructive feedback. We removed most of the abbreviations mentioned by the reviewer from the main text, except in the figures due to space constraints. However, we kept paired-associations abbreviated (PAs) as this is ubiquitous in schema and associative learning literature and appears in many locations within the manuscript. For the description of task phases, we chose to compress similar experiments with diverging session types in the experiment only when they deviated from the main timeline of the task (Fig. 1e, f), which we think was the most structured way to present our results.

On line 25 (page 6), it is unclear how the concept of pattern completion applies to the current design, given that the changed associations were not previously encoded and therefore cannot be "completed" from stored representations. More generally, this appears to be another example of inflated terminology that makes the manuscript harder to read and may not be necessary for the interpretation of the data.

We completely agree with the reviewer and now removed the pattern separation interpretation from the manuscript. In the revised manuscript, we removed "This discounts our first hypothetical mechanism that some original PAs acted as the framework of relational declarative facts and were necessary to facilitate assimilation of changed PAs through pattern completion". In doing so, we also numbered our hypotheses (the three that we introduced) in the Results section to match the ones we described earlier in the Introduction section. We believe this has substantially improved organization and readability of the manuscript, as now the Results section aligns to the core hypotheses from the Introduction section.

In Figure 1 I-J, performance for the unchanged PAs in blue appears to decrease during Asd blocks. How do the authors interpret this reduction? If assimilation operates through integration into a stable schema, one might expect preserved (or even enhanced) performance for unchanged associations rather than transient decrements.

We thank our reviewer for raising this point. Indeed, the blue lines representing unchanged PAs in Fig. 1i-j appear to decrease during assimilated blocks on Day 1, Group assimilation 1. To examine this more carefully, we conducted sets of analyses where we quantified the significance of animals' performances for unchanged PAs during assimilated sessions in group assimilation experiments and compared it against changed PAs in the same session. Performances for unchanged PAs were always significantly stronger than chance (i.e., no-cue session performances) and compared to the changed PAs during assimilation sessions and were similar to those for the changed PAs in the baseline and assimilated sessions across group assimilation 1, group assimilation 2, combined group assimilation sessions as well as in the combined group assimilation sessions from original and control stimulation batch of animals (Supplementary Fig. 2i-k). This indicates there was no significant change in performance compared to chance levels and compared to the changed PAs in the same session.

We found a significant reduction in performance for the unchanged PAs in the assimilated sessions when compared to unchanged PAs in the baseline sessions but not when compared to unchanged PAs in the assimilation sessions, only in group assimilation 1 Day 1 experiment. This reduction was not observed when we tested group assimilation 2 Day 2 experiment and when we combined data from the two days of group assimilation experiments. The performances for unchanged PAs reduced only in the assimilation session of the first group assimilation 1 Day 1 experiment when we introduced new PAs to trained animals for the first time in their life. We believe the first-time removal of 3/6 of the 5-week familiar PAs and the strong salience associated with the new cues (flavors that rats do not encounter naturally such as caramel and almond) may have caused an emotional response that might have interfered with their attention to the familiar cues reducing their performance. Additionally, for the first time,

our animals were making larger number of errors in the known paradigm for the changed PAs, which might have intermittently reduced their certainty/attention for familiar PAs as well. This reduction was not observed in any of the subsequent exposures to the novel PAs in the other group assimilation experiments as well as in the overall group assimilation experiments data. After their first group assimilation day, once animals were comfortable with the fact that some cues can change and were not innately harmful, we believe they were not affected by the PAs changes in the subsequent days. We have added these comparison results in the supplementary Fig. 2i, j and k.

The characterization of the performance improvement as "insight-like" may be overstated. The data appear to show some progressive within-session learning during Asn (for instance in Figure 1J), followed by further improvement after sleep.

We appreciate our reviewer's comment that there could be hints of within-session learning during the assimilation sessions, which we studied in further detail in the revised manuscript. Indeed, the line connecting the assimilation session performances for changed PAs trends positive during assimilation sessions in Fig. 1j. However, rats' performance reached significance only on block 2 of first group assimilation experiment before it dipped to chance levels in the subsequent, third block. The performances in the other assimilation days namely group assimilation 2 (Fig. 1j middle panel) and complete assimilation days (Fig. 1j right panel) for the changed PAs (in red) dropped to chance levels in all three blocks of the assimilation session in both of these experiment days – they were not significantly better than chance in any of these experiment days in any block of these assimilation sessions. Furthermore, we again combined data from group assimilation 1 and 2 experiments and observed no significant performance in the assimilation session in any block (revised Supplementary Fig. 2k) suggesting the effect observed in block 2 of Day 1 was critically contributed by the reduced sample size. The performances for changed PAs started off worse and did not improve beyond the chance levels in any of the three blocks of the combined Days 1-2 assimilation sessions. We also included our data from the control stimulation experiments (group assimilation) and found the rats' performance stayed at chance levels and did not improve beyond chance in any assimilation block of those experiments (revised Supplementary Fig. 2k). Therefore, we interpret that improved performance in block 2 on Day1 alone might have been contributed by the relative easiness of task (given 3 of the original PAs were still present) and by the use of other non-associative learning approaches, both of which were significantly less at play in the next days.

Intriguingly, despite only performing at chance for the changed PAs in the assimilation session blocks, after the post-assimilation/encoding sleep/rest, animals' performance was significantly better than chance for all 6 PAs. This improvement happened in concert for all 6 PAs, including during the complete assimilation Day when all 6 PAs were new, with no effects of serial position, primacy or recency of the new PAs (Supplementary Fig. 4). After analyzing and observing datasets in multiple separate experiments and in different batches of animals as well as specifically in the complete assimilation experiment, the concerted rise in animals' performance for *all 6 new PAs* after the post-encoding sleep is unlike any other forms of learning previously reported in rats. In most previous cases, learning is either incremental, typically involving a small number of associations, or quite rapid, involving innate natural behaviors (i.e. food caching memories in birds, context-associated fear memory in rodents etc.). The disruption of HPC ripples in the post-encoding sleep/rest impaired the concerted improvement in animals' performances for all new PAs in the next block after sleep but allowed uncoordinated within session improvement in some PAs in the post-disruption sessions. This lends additional support to the insight-like processes during the post-encoding sleep/rest ripples in this schema-based assimilation task.

The authors conclude that the observed changes in cue-responsive neurons discount the hypothesis of inferential replacement through feature-based associative inference. This conclusion appears also overstated. The reported analyses primarily show that cue identity representations change when cues are replaced, which is perhaps not surprising. However, serial inferential mechanism (e.g., NC->OC->GL) may not necessarily require preservation of cue-selective neuronal assemblies across sessions and could depend on more complex network dynamics beyond simple changes in firing rate (e.g., temporal coding or coordinated co-firing patterns).

We believe our reviewer is correct in pointing out that our finding of original cue-responsive neurons not repurposing to code for the newly changed equivalent cues do not eliminate the possibility of contribution from a feature-based associative inference mechanism. We agree that the feature-based associative inference could involve broader sets of known hippocampal coding mechanisms and could mechanistically involve different types of coding properties than tested here. Therefore, we removed the feature based associative inference from the interpretation of our findings in the revised manuscript. Instead, in the revised manuscript, we interpret this result as not supporting the learning of new PAs through *cue feature binding and transfer,* which we believe represents our results more appropriately.

On page 9, the authors propose a mechanism termed "map-based associative inference" to explain rapid learning in the task. However, it is not entirely clear in what sense the described process constitutes inference rather than rapid associative binding within a stable spatial environment. In other words, animals may learn the general rule of the task —associating cues with goal locations within a familiar allocentric context — a strategy that naturally relies on stable allocentric cues. It is therefore unclear whether the current data necessitate a novel learning mechanism.

Related to the previous point, while the analyses in Figures 2D–H demonstrate stable reward–location coding across sessions, it remains unclear how these findings uniquely support the proposed map-based associative inference mechanism. Stable goal–location representations in the hippocampus would be expected in a well-trained spatial task and may reflect preserved spatial and value coding rather than evidence of a distinct inferential process.

We appreciate our reviewer's comment, that gives us an opportunity to clarify our interpretation. We believe the reviewer asked us to contrast "map-based associative inference" and "rapid associative binding within a stable spatial environment". We agree with the reviewer that our finding aligns well with associative binding within a stable spatial environment if we only consider within-session dynamics of each learning session. However, there were 3 additional important observations that compelled us to interpret the results in terms of the map-based inference:

1. Results presented in Fig. 2d-h showed there was a generalization/invariance in the activation of representations of associative binding (both positive and negative associative code) from the baseline sessions to the assimilation and the assimilated sessions during rapid associative learning. In other words, our results indicated that i) there was a goal-specific outcome coding in HPC in all sessions if we look at within-session data (Supplementary Fig. 6d) and ii) when we analyzed similarity in these representations across sessions, the HPC ensemble that activated for positive outcomes at specific goals during baseline sessions activated similarly during rapid learning both in assimilation and assimilated sessions. Moreover, the ensembles that activated during negative outcomes were similar in their activation during baseline, assimilation, and assimilated sessions. This was true not only in the temporal representations at goal sampling, but also in the spatial firing properties of HPC neuronal ensemble.
2. We also observed outcome/reward related codes (outcome coding at goals) in the control experiment sessions (no-cue and allocentric-cue blocking) when we analyzed data within these control sessions (Supplementary Fig. 6d). However, we observed that the generalization/invariance of this code from baseline to the control sessions were impaired (Figs. 2e, g-h). In the control experiment days, in the no-cue session one key component of association was absent and in the allocentric-blocking, the allocentric

context was altered. The generalization/invariance of outcome specific coding from baseline to these control sessions was impaired during no-cue experiment days both at goal and in the spatial firing of place cell ensembles. Similarly, this generalization of outcome specific code from baseline to control sessions was poor in the spatial firing rates of the ensembles during allocentric blocking experiment days. To address the comment that "*Stable goal–location representations in the hippocampus would be expected in a well-trained spatial task and may reflect preserved spatial and value coding rather than evidence of a distinct inferential process.",* during the no-cue control day, our results showed significant reductions in these invariances/generalization during goal sampling both in the spatial and temporal domains, while having robust and stable place fields (Supplementary Fig. 5a-d). This is consistent with a scenario in which activation of these representations was primarily driven by the inferred cue-associated outcome (specific reward value associated with cue driven actions) rather than reward value alone, since similarly flavored rewards were delivered at the end of the trials in no-cue session as well. To reflect this point, we have added following statements in the Results section "This reduction occurred despite the no-cue session being similarly rewarded and rats behaving similarly compared to the assimilation sessions (Supplementary Fig. 9a-c). This indicates the invariant neural representation during rapid learning depended on the outcome being associated with the corresponding cues rather than the reward value."

3. The association of new cues with goals (goal location representations linked to correct associated cues) in assimilation sessions during and after goal sampling was followed by predictive association of cues to the goals at start arms (cue predictive of goal locations) guiding correct behavior in the assimilated sessions. We showed that predictive symmetrical representations (i.e., goal location association with cue identity at goal locations resulting in cue at start arm predicting the goal location) do not appear right away in assimilation sessions. This is because animals must visit multiple goal sites following presentation of 3 to 6 different PAs (disconnected at this point) to figure out which were the correct and incorrect cues and link these cues to the outcome associated goal representations (positive and negative) at each goal. The pre-existing schema knowledge, i.e., that one cue leads to positive reward at one goal and other cues lead to negative reward at the same goal (applied to multiple goal sites) and the neuronal activation of this knowledge, likely enabled these rapid predictive inferential activations. We posit these rapid predictive activities would not have occurred and animals would take several days to learn these new associations, closer to the original schema acquisition timeframe, in absence of this schema, which is accelerated compared to a simple rapid associative binding within a stable spatial environment. We have included this argument in the revised Result section as: "The across session invariant activation of distinct outcome codes is consistent with the activation of pre-existing rules of the schema, i.e., that one cue leads to positive reward at one goal and other cues lead to negative reward at the same goal (applied to multiple goal sites), enabling rapid inferential assimilation learning."

Thus, we think rapid generalization/invariance of these association-bindings throughout the learning sessions (that were mapped onto the goal locations) could be inferential, because they were generalized to other learning sessions and activated rapidly in the first block in the assimilation sessions (see Supplementary Fig. 10). These activations link specific goals to the specific cues during three exposures for each PA in the assimilation sessions. Moreover, the observation of generalization of goal location-based (mapping) of the outcome codes from baseline to other sessions during rapid associative learning 1) is similar to what was predicted in the literature[4,7,8,10-12] and 2) aligns well with the associative inference idea based on relationally

mapping rules of association in HPC network. Our results provide evidence that these relationally mapped rules can be activated to learn new functionally equivalent associations in the unchanged map (allocentric context). Although we might have introduced the term map-based associative inference here for the first time, the idea is not entirely new and has been previously used in the literature[6-9] in relation to the role of HPC in associative learning and schema formation.

In the revised manuscript, we split the original 6-panel Figure 2H that demonstrated the comparison of the invariances/generalization between different experiment days, into two panels Fig. 2g and Fig. 2h, one for comparison of invariances in temporal firing properties and another for comparison of invariances in spatial firing properties. Thanks partly to the reviewer's comment, we believe this change will make the results more readable.

The Bayesian decoding analyses in Figure 3 are particularly interesting and provide compelling evidence for predictive activation of goal-specific representations during cue sampling. However, it remains unclear whether such predictive activity necessarily reflects inferential computation, as opposed to rapid retrieval of newly formed cue–goal associations following consolidation. Also, although this effect is most prominent during the assimilated sessions, the cue–goal contingencies were already experienced during the preceding assimilation session and may have been progressively strengthened during that phase or the subsequent sleep phase.

We thank the reviewer for finding the results in Fig. 3 interesting. The reviewer is asking how we interpreted this as an inferential computation as opposed to rapid retrieval of cue-goal associations following consolidation. We believe the reviewer is correct in raising the possibility that this process could be memory/learning-based prediction of future goals at the time of cue presentation after new learning. We provide the following reasons for the interpretation of this result as inferential:

1. The prediction is most likely aided by the memory of a rule rather than an episodic memory. The reason we think that it might be an abstract rule rather than an episodic memory of the goal, is that there were 6 correct associations over 18 distinct trials and multiple incorrect choices (i.e., multiple episodes) and the standard what-where-when feature of episodic memory would not be sufficient to solve the task after only 3 correct trials/association. To prove that, the original learning of 6 such associations took around 5 weeks of training. However, schema formation and abstract rule learning (i.e., the knowledge of positive and negative associations, that one cue, but no other cues, leads to one specific goal per cue) is what helped rats link/infer which association needed to be activated at the cue presentation. An episodic activation without this abstract knowledge of rules likely would not be so rapid and would require longer periods or counts of exposures to the associations. Rather than remembering 6 episodes of correct choices out of the multiple correct and incorrect choices taken during an assimilation session, we posit the rats learned to apply the schema rule to the 3-6 new associations via an inference process.
2. We did not think the cue-induced predictive activation of goal representations were associated with general planning or decision because we did not observe it during any other session types (baseline, control sessions and assimilation session). In addition, we did not observe the predictions of next goal choices (possible planned visit) during incorrect trials even in the assimilated sessions.
3. We thought these activations were primarily predictive and/or inferential because only zero-error correct trials showed this predictive activation of specific goal representations when corresponding cues were provided at start arms at the beginning of trials. The important finding that the correct goal representations did not activate in the start arms at cue presentations when rats made one or more errors during incorrect trials, points at this predictive activation containing correct goal predictive/inferential computation, which

activated only during correct trials. If these were linked to rapid retrieval of newly formed cue–goal associations following consolidation and not to the inferential/predictive computation, we would expect to observe these goal activations during the incorrect trials in the assimilated sessions, but that did not happen.

4. The emergence of performance better than chance after new learning and detection of the goal predictive activation at the start of the correct trials convinced us that these were correlated. In other words, goal predictive representations seem to have guided animals toward correct goal-associated behavior exactly when new-cue goal inferences (new PAs were inferred based on previously known association/rules) appeared, in concert for all the PAs (Supplementary Fig. 4).

The above arguments are now included in the revised Results section as: "The lack of prediction in all the other sessions except the *assimilated* session and in the incorrect trials implies an inferential activation of the latent causes associated with the positive action-toward-goal resulting in the positive outcome at goals, which likely guided the correct behavior during rapid inferential learning."

The ripple decoding analyses in Figure 4 are also very interesting, and the ripple perturbation experiment in Figure 6 is particularly impressive. However, as noted in previous points, animals were already exposed to the new PAs during the preceding assimilation session. It therefore remains unclear whether these findings require a genuinely generative network reconfiguration during sleep, rather than being parsimoniously explained by replay and consolidation of recently encoded cue–goal contingencies.

We appreciate that our reviewer found the results in Figures 4 and 6 interesting. We have the following arguments to support the idea that our results require generative reconfiguration and cannot be solely described by the replay and consolidation of recently experienced cue-goal contingencies:

1. Traditionally, replay has been described as a serial/sequential pattern of unimodal activations that were related to the experiences on the maze. However, offline HPC generative reconfiguration during post-encoding sleep/rest likely linked numerous (3-6) new associations across two modalities (flavor and place) simultaneously, according to a previously learned abstract rule via orderless neuronal co-activity. In particular, cue-responsive neurons and place cells on changed PAs goal arms have not fired together during the HPC frames occurring in the encoding phase but co-activated only during the sleep/rest session post-encoding via an offline generative reconfiguration process rather than a traditional replay of previously similarly occurring temporally-coordinated activity (Supplementary Fig. 13g). We have added a plot/figure (Supplementary Fig. 13g) showing that changed PAs cue-place cell coactivation corresponding to future/assimilated, in addition to those corresponding to past/assimilation, predictively occurred during the post-encoding sleep/rest session.
2. Neuronal representation of memory consolidation in the neuroscience literature predominantly presents evidence for the reactivation or coactivation of novel or recently encoded memories. However, our results showed that mPFC-HPC ensemble co-activity predictively activated the unchanged PAs. The HPC activation of the changed (novel) associated cue-goal location pairs in isolation within frames of sleep/rest ripples and of the unchanged (familiar) during cofiring with the mPFC single units together geared toward future distinct assimilated session neural correlates. This suggests a network reconfiguration, allowing for the rapid evolution of both the novel and familiar memories within mPFC and HPC networks. Integration of novel and preservation of stable familiar associations are integral to rapid learning in schema-based framework. We also detected signature coordination demonstrating slow integration of novel association

memory within HPC-mPFC co-activities two days later, termed in literature as systems consolidation, which is distinct from the rapid network reconfiguration that we described during rapid learning.

3. The mPFC outcome memory-generalizing cell-assemblies and the cue-responsive cells active in future *assimilated* sessions had higher synchronization with HPC ripples during post-encoding sleep/rest in all assimilation learning days. This was not the case during the pre-return-to-baseline sleep/rest sessions in control days (Figs. 5h, i and Supplementary Fig. 16d, e), suggesting this is not simply a reactivation/replay but rather a re-configuration of the mPFC network in coordination with HPC ripples during new learning.
4. This sleep/rest related generative network reconfiguration in HPC preceded the improved behavioral performance and was more likely to support an *active* role of sleep/rest in insight-like learning and abstract problem-solving compared to a traditional replay of previously experienced serial patterns of activity. We demonstrated impaired rapid association learning, but preservation of slower traditional learning, when HPC ripples in this post-encoding sleep/rest were disrupted. This implies the offline ripple related reconfiguration might be essential for the inferential/insight-like learning but not for the slower forms of learning based only on consolidation of new experiences.
5. We did not emphasize this result before, but in all of the analyses in which we tested the offline sleep/rest activities, the results were similar and significant also in the pre-encoding sleep sessions (Fig. 4c-j; compare pre-assimilation and pre-assimilated sleep/rest results) *before* we introduced new cue-place association pairs. This points towards the possibility that the associative learning framework (representation and coactivities) in offline sleep/rest states dynamically and predictively (re)configures in anticipation of newer learning based on pre-existing associative framework.
6. Furthermore, we have conducted new analyses to compute the new cue-goal location coactivations in HPC during post-encoding sleep/rest for cell pairs detected in assimilation session compared to pairs detected in the assimilated sessions. We included this information in our revised manuscript as Supplementary Fig. 13f, g. We found that both the cell pairs (cue-location) in assimilation and assimilated sessions were significantly biased toward changed compared to unchanged PAs in the post-encoding sleep/rest (left panel in Supplementary Fig. 13g). However, we observed that in all the sleep sessions, these coactivities were not stronger for the cue-location pairs from assimilation session (i.e., *past*) compared to the *future* assimilated session. This suggests that the coactivities of new cue-location cell pairs representing assimilation experience (i.e., past) were at similar levels when compared to the predictive *future* cue-location cell pairs representing predictions/reconfigurations. This is in contrast to the expectations from the traditional view of sleep/rest replaying the past but not the distinct future neuronal ensemble activity.
7. Finally, we calculated the coactivities of the cue-location cell pairs associated with the changed PAs, which were found elevated in the post-encoding sleep, now computed during waking rest epochs frames on the 8-arm maze (multiunit activations during awake immobility periods on the maze). We found that the *assimilation* session waking rest cue-location cell pairs coactivities for changed PAs were elevated both during assimilation and assimilated sessions (as well as in the post-encoding sleep/rest), indicating a likely consolidation of recently experienced new PAs (right panel in Supplementary Fig. 13g). This matches what the reviewer suggested and what has been previously reported in the literature. However, we also found that the *assimilated* session cue-location cell pairs coactivities for changed PAs were elevated in the assimilated, *but not in the assimilation* waking rest learning sessions indicating a predictive

reconfiguration of these future correlates in the intermingled post-encoding sleep (right panel in Supplementary Fig. 13g). These coactivities of reconfigured cue-location cell pairs were elevated during post-encoding sleep (i.e., not present during encoding of new experiences/assimilation) at similar levels as those of the consolidation-related cue-location cell pairs. This suggests that there exists a predictive reconfiguration in addition to the consolidation of newly experienced cue-location coding cell pairs.

We made the following changes in the manuscript in the result section to incorporate and address the reviewers rightful concern, "We observed that the changed cue-location coding cell pairs in the *assimilation* session elevated their coactivities during post-encoding sleep/rest consistent with their consolidation during post-encoding sleep/rest (Supplementary Fig. 13f, g) and previous reports[13-15]. In addition, we found that coactivities of the changed PAs in the *assimilated* session were not elevated during the *assimilation* session and only appeared elevated during the post-encoding sleep/rest frames followed by their strengthening in the *assimilated* session (Supplementary Fig. 13g). The increases in encoding-related coactivity reactivation during post-encoding sleep/rest were not greater than those related to the reconfiguration (Supplementary Fig. 13f, g). This suggests that there exists a predictive reconfiguration in addition to the consolidation of newly experienced cue-location coding cell pairs during post-encoding sleep/rest."

Some minor points:

The authors state that rats first learn three "non-associative task rules" early during training that account for part of the performance improvement by day 10: (1) avoiding the non-cued start arm, (2) avoiding arms already visited within a trial (within-trial working memory), and (3) avoiding the arm rewarded in the previous trial of the block. This is a strong claim that appears somewhat peripheral to the main message of the manuscript and is not fully supported by the data presented. It remains unclear whether these behaviors reflect independently acquired abstract rules, as they overlap conceptually and seem to emerge in parallel with associative learning. The supplementary data (Figure S1A–C) show concurrent improvements but do not directly demonstrate rule acquisition. The authors should clarify this point and temper their claims accordingly. We agree with the reviewer that these claims appear somewhat peripheral to the main message of the manuscript. We have removed these claims in the revised manuscript and only acknowledge these improvements in early learning as "The rats first improved their performance from 17.62±5.6% (4.1±0.3 errors/trial) on the 1st day to 56.34±5.4% (2.2±0.3 errors/trial) on the 10th day by not visiting 1) the start arm not cued during the block, 2) the arms already visited during the trial, and 3) the arm rewarded in the previous trial of the block (Supplementary Fig. 1a-c)." Overall, we think that learning to utilize these strategies helped rats reach the chance levels (observed on no-cue test session) early during the learning, but we refrained from claiming the exclusive utilization of these strategies, as the reviewer suggested.

What fraction of the trained rats successfully acquired the task?

We have revised the method section to address this question. Revised method section now states "Eighteen Long Evans male rats (10-12 weeks old at the beginning of experiment) were handled and habituated in the maze for 5 days. Twelve of these rats were selected for further training due to their improved behavior during maze habituation phase. Eleven of them were successfully trained over 6-8 weeks and acquired a complex mental schema of 6 flavor-place associations task. One of the twelve rats was dropped out in the middle of the training due to lack of significant improvement in behavioral performance." These include all the rats that participated in the experiments including the stimulation experiments.

References

1 Chettih, S. N., Mackevicius, E. L., Hale, S. & Aronov, D. Barcoding of episodic memories in the hippocampus of a food-caching bird. *Cell* **187**, 1922-1935 e1920, doi:10.1016/j.cell.2024.02.032 (2024).

2 Eichenbaum, H. & Cohen, N. J. Can we reconcile the declarative memory and spatial navigation views on hippocampal function? *Neuron* **83**, 764-770, doi:10.1016/j.neuron.2014.07.032 (2014).

3 Whittington, J. C. R. *et al.* The Tolman-Eichenbaum Machine: Unifying Space and Relational Memory through Generalization in the Hippocampal Formation. *Cell* **183**, 1249-1263 e1223, doi:10.1016/j.cell.2020.10.024 (2020).

4 Zeithamova, D., Dominick, A. L. & Preston, A. R. Hippocampal and ventral medial prefrontal activation during retrieval-mediated learning supports novel inference. *Neuron* **75**, 168-179, doi:10.1016/j.neuron.2012.05.010 (2012).

5 Schlichting, M. L., Mumford, J. A. & Preston, A. R. Learning-related representational changes reveal dissociable integration and separation signatures in the hippocampus and prefrontal cortex. *Nat Commun* **6**, 8151, doi:10.1038/ncomms9151 (2015).

6 Ghosh, V. E. & Gilboa, A. What is a memory schema? A historical perspective on current neuroscience literature. *Neuropsychologia* **53**, 104-114, doi:10.1016/j.neuropsychologia.2013.11.010 (2014).

7 Tse, D. *et al.* Schemas and memory consolidation. *Science* **316**, 76-82, doi:10.1126/science.1135935 (2007).

8 Tse, D. *et al.* Schema-dependent gene activation and memory encoding in neocortex. *Science* **333**, 891-895, doi:10.1126/science.1205274 (2011).

9 Alonso, A., van der Meij, J., Tse, D. & Genzel, L. Naive to expert: Considering the role of previous knowledge in memory. *Brain Neurosci Adv* **4**, 2398212820948686, doi:10.1177/2398212820948686 (2020).

10 Yang, W. *et al.* Selection of experience for memory by hippocampal sharp wave ripples. *Science* **383**, 1478-1483, doi:10.1126/science.adk8261 (2024).

11 Dusek, J. A. & Eichenbaum, H. The hippocampus and memory for orderly stimulus relations. *Proc Natl Acad Sci U S A* **94**, 7109-7114, doi:10.1073/pnas.94.13.7109 (1997).

12 Bunsey, M. & Eichenbaum, H. Conservation of hippocampal memory function in rats and humans. *Nature* **379**, 255-257, doi:10.1038/379255a0 (1996).

13 Buzsaki, G. Two-stage model of memory trace formation: a role for "noisy" brain states. *Neuroscience* **31**, 551-570, doi:10.1016/0306-4522(89)90423-5 (1989).

14 Girardeau, G., Benchenane, K., Wiener, S. I., Buzsaki, G. & Zugaro, M. B. Selective suppression of hippocampal ripples impairs spatial memory. *Nat Neurosci* **12**, 1222-1223, doi:10.1038/nn.2384 (2009).

15 Fernandez-Ruiz, A. *et al.* Long-duration hippocampal sharp wave ripples improve memory. *Science* **364**, 1082-1086, doi:10.1126/science.aax0758 (2019).

Reviewer #1 (Remarks to the Author):

In this manuscript, the authors design a complex cross-modal task in which rats learn cue-location paired associations over several weeks. This allowed rats to generate a mental schema that allowed to learn within a single day. The main findings are that hippocampal cells generate cue-location relationships to make inferential connections during the task and hippocampal along with PFC cells, reorganize their activity patterns during offline rest/sleep periods. The level of this reorganization in the hippocampus predicted learning, and this information was transferred to the PFC during ripples.

The findings are important and of high significance for the field, as they add to our knowledge of how the hippocampus and PFC coordinate to generate learning schemas.

The conclusions are supported by the data and the manuscript is written in a easy way to follow for the broad audience.

In the revised version of the manuscript, the authors have addressed all of my concerns and together with the points that they added to address all other reviewers concerns, the manuscript is ready for publication as is.

We thank the reviewer for their constructive suggestions that helped us revise and improve our manuscript.

Reviewer #2 (Remarks to the Author):

Through revision, the manuscript has improved in its clarity of presentation. The hypotheses are stated more clearly, however, I still feel that many analyses are not clearly grounded in the hypotheses being tested and that conclusions extend beyond the data.

I will provide some examples that illustrate a recurring logical flaw with the following structure: (A) the authors introduce a hypothesized cognitive algorithm, (B) then introduce a hypothesized implementation of that algorithm, (C) then conduct an analysis which is a proxy for that implementation, then based on that analysis, they (D) reject/accept the hypothesized cognitive algorithm.

We thank the reviewer for providing us with an opportunity to clarify our logical process. Indeed, we first emitted hypotheses. We then generated testable and falsifiable predictions based on our hypotheses. We next tested our predictions. Finally, we concluded about the probability of hypotheses being true or false based on our testing of the predictions derived from our hypotheses. Not uncommonly for the field of neuroscience, not all possible predictions based on the hypotheses could be tested or falsified.

Example 1:
A) "Our first hypothesis was that an inferential mental substitution of the original cue with the new cue, both associated with the same goal location, could occur via a neural mechanism of direct cue feature binding and transfer"
B) "One of the ways this could work is via the activation of the representation of the original cue during the encoding of the new cue-goal association at the goal location"
C) "In complete assimilation day, the proportion of cue-responsive cells maintaining cue-responsiveness from baseline to other sessions (i.e., new, different cues) was smaller compared to the proportion that did so from assimilation and assimilated sessions to the other assimilation sessions in HPC (i.e., same cues)"
D) "Therefore, when cue identities changed, network cue-responsiveness changed as well46, further discounting our first hypothesis of inferential replacement of PAs through direct cue feature binding and transfer"

The analysis simply shows that when the task demands (and stimuli) change, hippocampal and mPFC neurons fire more differently than when task demands stay the same. To say that neurons should behave identically is a strawman argument. The real comparison is whether the neural firing is more similar during assimilation than during another situation in which task demands and world stimuli are equally different, with the exception that no schema can be used to guide learning.

The hypothesis of a neural mechanism of direct cue feature binding and transfer during assimilation into schema has a basis in known hippocampal literature which showed that generalized feature representation (cues and locations) during inferential learning aids in the *simpler forms* of inferential learning, which are usually not based on cognitive maps [Zeithamova 2012; Schlichting et al 2015; Mckenzie 2013]. The literature suggests that there exists a possibility of generalized representation of

replaced cue and/or place when the cues were changed during inferential learning. In other words, reactivation of memories of old cue when the new cue substitutes it in the same location is a distinct possibility and not merely a strawman argument. For instance, in a study conducted by Mckenzie et al. 2013, animals inferentially learned new goal location through a generalized activation of original goal location features. In our task there were multiple associations linking specific cues with distinct locations. So, inference through generalization of cue, location or the associated outcomes, were all plausible mechanisms. In other task design or demands, similar types of generalization about other task features might contribute to learning. In our design, we did not change cues or PAs while conducting other task demands because these were outside the scope of our experiments, and, consequently, we are not in a position to directly fulfill our reviewer's request. However, we compared the neuronal tuning at the changed cue locations with tuning at the unchanged cue locations during the same task conditions and in the same sessions (Supplementary Fig. 5f, g and i). If the direct new-old cue binding at the same goal was sufficient to explain assimilation into schema learning, we believe there should be more similarity between the changed and unchanged cue-responses, comparable to that observed for the unchanged cues. Therefore, we feel that in addressing point C), in addition to what was mentioned by our reviewer, we should also include: "Both HPC and mPFC ensembles of cells responsive to the unchanged cues maintained a significantly greater similarity in their cue-activation between baseline and assimilation sessions (i.e., correlation 0.29±0.02 for HPC, 0.32±0.03 for mPFC ensembles) compared to cells responsive to the changed cues (i.e., 0.10±0.03 for HPC, 0.21±0.03 for mPFC ensembles; Supplementary Fig. 5f, g). Moreover, we found a reduced overlap in cue-responsive HPC cells (Supplementary Fig. 5h) that maintained corresponding cue responsiveness from baseline to other sessions for the changed (15.60±3.4%) compared to unchanged cues (35.94±4.6%) (P=7.3x10-3; Supplementary Fig. 5i)." In addition, to address our reviewer's concern, we further qualified our experimental findings in the revised manuscript: "We could not directly compare the overlap in the network representation of changed cues with the original baseline cues during schema-based learning to the ones in non-schema learning conditions due to the different focus of our experiment design. Therefore, when cue identities changed, network cue-responsiveness changed as well[46] compared to the cue-responsiveness to the unchanged cues, suggesting that the inferential learning in this schema based task might not have happened solely through the representational generalization of cue features, i.e., *our first hypothesis* of *direct cue feature binding and transfer."*

The cue responding neurons were originally quantified in comparison to the trial-ID shuffles. In the no-cue test session, the task demand was altered (non-associative) and reward cues were not changed while in the allocentric-block session cues were not changed and the task demand was still the same. Interestingly, in the no-cue session (Supplementary Fig 5i) we see similar neuronal responses to no-cue (i.e., chance level) compared to the changed cues and in the allocentric-block session we see similar results to the unchanged cues compared to the learning sessions. This suggests that 1) the responses to the original vs. the changed cues are not more similar than chance (i.e., responses to cue vs. no cue presentation) in our schema-based learning experimental design, and 2) a neuronal cue response maintained at the level of an unchanged cue, as in the allocentric block session, is not sufficient to warrant successful behavioral performance or new learning. We added this paragraph to the revised manuscript.

Example 2
A) "Our second hypothesis was that assimilation of the new PAs happened by activating and associating invariant neuronal representations linked to the task framework, namely a direct association of goal locations with the corresponding correct cues in this task"
B) "We hypothesized that these invariant neuronal representations co-activated and updated the new cue-outcome contingencies (positive, rewarded as well as negative, not rewarded) at goal locations"

C) "we observed that HPC single units that responded to reward at specific goal locations in the baseline session during learning consistently responded to the reward at the same goal location in assimilation and assimilated sessions irrespective of whether the PAs were changed or unchanged at the goal (Fig. 2d and Supplementary Fig. 6b).
D) "Despite the increased variability in outcome-invariant activations, the resulting animal behavior and HPC theta power on both these outcome types were not different between the no-cue session and the assimilation sessions of the learning days. This indicates that only during the rapid learning processes the goal-specific outcome-invariant responses inferentially linked the new cues to the goal-specific outcome, similar to the corresponding original cues."

I am not sure that a priori, one could have had an intuition for what "increased variability in outcome-invariant activations" could say about map-based inference. The analysis still needs to be better grounded. In my reading the authors find place cells (neurons that fire in an arm irrespective of what is cued there) and then make a grand theory based on that standard observation. The author analyze theta power, and then immediately following, conclude that "this indicates ..." What does "this" refer to here? That null is what needs to be clearly rejected.

We thank our reviewer for suggesting we improve the clarity in testing and reporting on the map-based hypothesis. In the section related to this hypothesis, we started with the observation that the neural representations (i.e., population vector 'PV' correlations during goal sampling) in the assimilation sessions were more similar at each goal, both during positive outcomes and negative outcomes, compared to that the PV correlations during the baseline and assimilated sessions, as indicated in the RSM matrix diagonals (Fig. 2e). These PV correlations (diagonals in RSM) were, however, reduced and much more variable in the no-cue control experiments (as indicated by the RSM diagonals; Fig. 2e top-right panel) despite animals getting identical rewards and no-reward during positive and negative outcome visits, respectively, during the goal sampling periods. This was an indication that rapid generalization of outcome at each goal during the assimilation session through reactivation of the outcome-specific neural representation from the baseline session could contribute to the rapid learning process. In order to rule out other factors that could result in this difference in the outcome-invariant neural activation between assimilation learning and no-cue control experiment sessions, we tested and found no difference in the movement velocity as well as in the theta power in these periods between these experiment conditions. Therefore, the factor that was likely to produce this difference was rapid assimilation learning. This was merely an indication from these observations of the RSMs, which we statistically tested in the following paragraphs to further establish this mechanism as the most likely neural mechanism that animal used in solving this task. Therefore, 'this' refers to the results in which we did not find any other factors contributing to the elevated across-session outcome-invariant responses (RSM matrix diagonals Fig. 2e) in the assimilation learning experiments, when compared to the reduced activation during no-cue control experiment.

The hypothesis tested here has two null predictions in this section 1) The increase in across-session PV correlations (diagonally shown in figure Fig. 2e) was due to the outcome itself and would be equally elevated in the learning and non-learning control experiment sessions, and 2) Behavioral factors (movement velocity, deviation on track, theta state etc.) might be different in between these experimental conditions which might contribute to the difference in across-session PV correlations. Our findings in this section rejected both of these null predictions and indicated that the increased across-session PV correlations (diagonally shown in figure Fig. 2e) was likely related to the schema-based learning and not on these other behavioral factors. In the subsequent paragraphs, we directly compared the percentage variance in the neuronal similarities explained by these across session outcome-invariant activations among different experimental conditions to further test the indication of the above hypothesis before drawing our conclusion.

We agree with our reviewer that the way we presented the arguments could be made simpler for the general readers. Consequently, we made the following changes to this section, which we believe have made it easier to read: "These outcome-specific invariant responses reduced and became more variable across sessions in the no-cue control experiment day (Fig. 2e and Supplementary Fig. 8).

Despite the reduction in across-session outcome-invariant activations, the animal behavior and HPC theta power on both these outcome types were not different between the no-cue sessions and the *assimilation* sessions of the learning days (Supplementary Fig. 9a-c). This indicates that the increase in outcome-invariant responses across-sessions during schema-based learning compared to the control day experiment was not due to the differences in reward, behavioral or theta oscillation state of the animals. Therefore, these results suggest that the cross-session goal-specific outcome-invariant responses likely linked the new cues to the goal-specific outcome, similar to the corresponding original cues, contributing to the rapid assimilation process."

In summary, I think the results are interesting but the interpretation needs to stay closer to the data and each analysis needs to be very clearly motivated about what it is testing exactly.

We thank the reviewer for helping us improve the manuscript substantially. We believe, with this revision, we have brought better clarity to the motivations of the hypotheses, the predictions based on the hypotheses, the evidence-based tests for those predictions, and the subsequent conclusions.

Reviewer #3 (Remarks to the Author):

The authors have thoroughly addressed my concerns. I have no further comments and support publication.

We thank our reviewer for helping us to substantially improve the manuscript through the constructive feedback and suggestions.
